# Aminothiolation of alkenes with azoles and Bunte salts

**DOI:** 10.3389/fchem.2023.1328441

**Published:** 2024-01-22

**Authors:** Bingqing Ouyang, Xing Chai, Zhe Li, Chunling Zhang, Xingmei Liu

**Affiliations:** ^1^ Department of Pharmacy, The Second Norman Bethune Hospital of Jilin University, Changchun, China; ^2^ Outpatient Department, The Second Norman Bethune Hospital of Jilin University, Changchun, China

**Keywords:** aminothiolation, Bunte salts, alkenes, metal-free, multi-component cascade reactions

## Abstract

We have developed an intermolecular aminothiolation of simple olefins using Bunte salt as a thiolating agent. This protocol produces thiyl free radicals under PIDA oxidation conditions, eliminating the need for transition-metal catalysts. The method has a wide range of substrate applicability and is suitable for large-scale preparation and late-stage modification of drug molecules.

## Introduction

**SCHEME 1:**
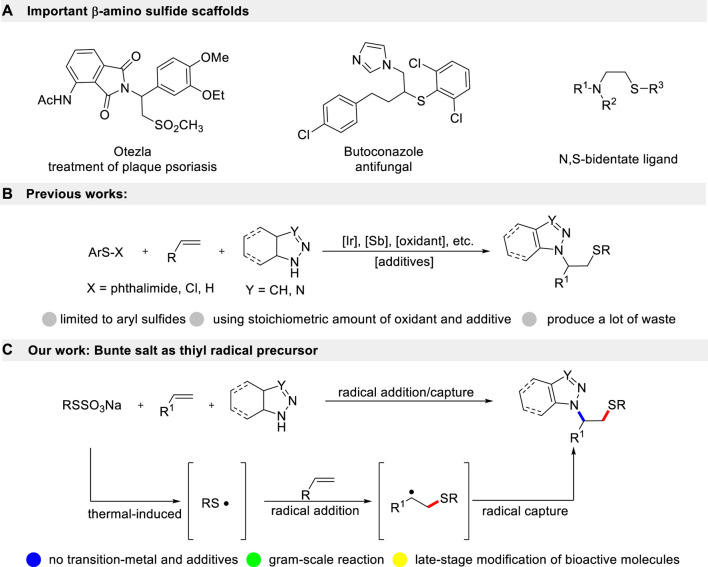
Aminothiolation of alkenes.

The simultaneous introduction of sulfur and nitrogen moieties into small molecules is a classical approach for designing new drugs. β-Amino sulfide is an important and widely present scaffold in natural products, best-selling drugs, and small molecular catalysts (Scheme 1A) (Cecchetti et al., 2003; Hosseini-Zare et al., 2011). For a long time, synthetic chemists have devoted considerable effort to develop novel and efficient strategies for the preparation of these significant frameworks (De Paolis et al., 2002; Jiang et al., 2016; Cui et al., 2019). Olefin is a readily available industrial raw material. Direct aminothiolation of olefin, achieved by selecting suitable amination and thiolation reagents, is one of the simplest and most effective strategies for constructing β-amino sulfide compounds (Scheme 1B). This method offers the advantages of high economic feasibility and simplicity of steps. Traditionally, the Ritter-type aminothiolation of alkenes utilizes diaryl disulfides and aryl thiols as thiolation reagents. These reactions are typically carried out in acetonitrile as the solvent, with nucleophile properties, and require stoichiometric amounts of transition-metal or strong acid conditions (Luo et al., 2015; Cui et al., 2016). These reactions proceed first undergo the formation of thiiranium ion intermediates, followed by a subsequent ring-opening reaction with acetonitrile. Soon afterward, Prof. Sun accomplished a breakthrough in I_2_-promoted aminothiolation of alkenes using azoles as nucleophiles (Sun et al., 2017). Recently, *N*-phenyl-sulfenyl phthalimide and ArSCl have also been employed as electrophilic thiolation reagents in the aminothiolation of alkenes [9,10]. Given the significance of β-amino sulfides derivatives, the groups of Zhu and Nishihara demonstrated a copper-catalyzed radical thioamidation of alkenes with NFSI and thiols (Li et al., 2017; Iwasaki et al., 2019). In 2018, Prof. Lei described an electrochemical oxidative aminosulfenylation of alkenes (Yuan et al., 2018). Very recently, Deng’s group disclosed that NIS could be used as both a radical initiator and an N-nucleophile, enabling a metal-free amidosulfenylation of alkenes with disulfides (Yao et al., 2023). However, there are common problems associated with these methods. They often require more expensive and toxic transition-metal catalysts. Moreover, they necessitate a large excess amount of Lewis acids, which often leads to the presence of expensive heavy metal residues. Additionally, these methods require complex post-treatment processes and can result in environmental pollution. At the same time, trace metal ions have a tendency to coordinate with β-amino sulfide compounds, which can affect the purity of the target product. This often necessitates additional filtration steps to remove the metal ions, resulting in reduced economic efficiency and hindering their potential application in the pharmaceutical industry. Therefore, the development of an environmentally friendly and practical protocol for the multi-component aminothiolation of alkenes without the use of catalysts is still a desirable and challenging task.

In recent years, Bunte salts have emerged as alternative reagents for thiolation in the formation of new C-S bonds. They have gained popularity due to their ease of preparation and handling. Traditionally, it is frequently used in transition-metal catalyzed cross-coupling reactions (Qiao et al., 2015; Liu and Yi, 2018; Wang et al., 2023; Wu et al., 2023). However, these methods require a toxic transition-metal catalyst, which not only limits the range of substrates but also generates a significant amount of environmentally unfriendly by-products. Herein, we describe an environmentally friendly and practical method for aminothiolation of alkenes using Bunte salts and azoles under metal-free reaction conditions (Scheme 1C). As per our design, Bunte salts would generate thiyl radicals through a single-electron oxidation. Then, the resultant thiyl radical undergoes region-selective addition with alkenes to produce the alkyl radical intermediate, which is captured by azoles to give the correspondingβ-amino sulfides. Therefore, through this radical addition/radical capture process, the use of transition-metal could be completely avoided.

## Results and discussion

Initially, we prepared a 100 mmol scale of CH_3_SSO_3_Na through nucleophilic substitution of iodomethane with sodium thiosulfate. We selected styrene (**1a**), benzotriazole (**2a**) and CH_3_SSO_3_Na (**3a**) as model substrates to verify the optimal reaction conditions (Table 1). Owing to their high efficiency, broad spectrum, and inward-absorbent properties, benzotriazole compounds are used to prevent epiphyte growth. Therefore, it is particularly important to develop green and efficient methods for constructing benzotriazole derivatives and functionalizing their core skeletons. According to previous literature research, various copper catalysts were preferentially selected. As a result, the desired reaction proceeded smoothly, yielding **4a** in 12% yield (entry 4). Inspired by this result, the influences of different oxidants were further examined (entries 1–6). TBHP, K_2_S_2_O_8_, DTBP were not efficient oxidants and could not provide the target product. To our degliht, the yield of **4a** increased to 81% using PIDA as an oxidant and CH_3_CN as a solvent at 80 °C under an O_2_ atmosphere for 24 h.

**TABLE 1:** Reaction optimization^a^.

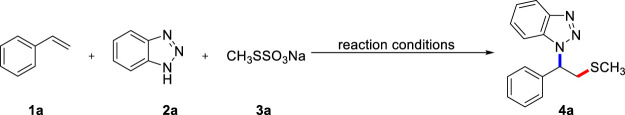				
Entry	Oxidant	Solvent	Temp (°C)	Yield (%)^b^
1	TBHP	CH_3_CN	80	0
2	K_2_S_2_O_8_	CH_3_CN	80	0
3	DTBP	CH_3_CN	80	0
4	CuBr_2_	CH_3_CN	80	12
5	PIDA	CH_3_CN	80	81
6	NIS	CH_3_CN	80	29
7	PIDA	toluene	80	0
8	PIDA	DCE	80	0
9	PIDA	DMF	80	trace
10	PIDA	THF	80	trace
11	PIDA	MeOH	80	0
12^c^	PIDA	CH_3_CN	80	43
13^d^	PIDA	CH_3_CN	70	77
14^e^	PIDA	CH_3_CN	90	72

^a^ Reaction conditions: **1a** (0.2 mmol), **2a** (0.4 mmol), **3a** (0.4 mmol), oxidant (0.4 mmol), solvent (2.0 mL) under O_2_, heated at 80 °C for 24 h.

^b^ Isolated yield.

^c^ Under N_2_ atmosphere.

^d^ At 90 °C.

^e^ At 70 °C.

Next, we screened different reaction solvents and found that CH_3_CN was the best choice for this transformation. No more satisfactory results were obtained when the reaction was performed with other polar, non-polar, and weak coordination solvents (entries 7–11). It is worth mentioning that the reaction can be carried out in a nitrogen atmosphere, but the yield is reduced (entry 12). Both increasing and decreasing the reaction temperature affect the conversion of the starting material and the yield of the corresponding product (entries 13, 14). Importantly, the hydroamination of alkene with azole is not observed during the process of optimizing reaction conditions.

**SCHEME 2:**
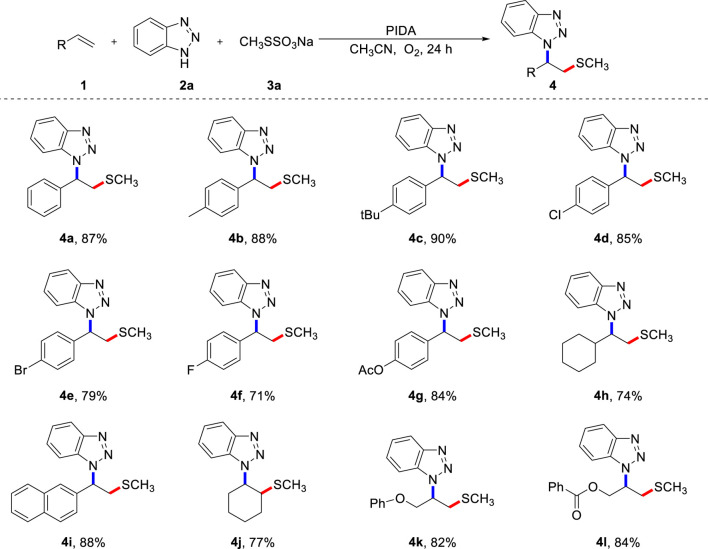
Scope of alkenes **(A)**. **(A)** Reaction conditions: **1** (0.2 mmol), **2** (0.4 mmol), **3a** (0.4 mmol), PIDA (0.4 mmol), CH_3_CN (2.0 mL) under O_2_, heated at 80 °C for 24 h, isolated yield.

Next, we examined the generality of this three-component cascade reaction with various alkenes using the optimal reaction conditions (Scheme 2). Methylthio is an essential functional group found in numerous anti-tumor active molecules, candidate drug molecules, and natural products. Therefore, the rapid introduction of methylthio functional groups into small organic molecules is significant for the development and discovery of sulfide drugs. Generally speaking, styrenes bearing both electron-donating and electron-withdrawing functional groups had little impact on the conversion outcome and all resulted in the corresponding products with high yields. Besides the good compatibility of aryl alkenes, alkyl alkenes, such as cyclohexane, also worked well in this transformation (**4h**). 2-Vinylnaphthalene was subjected to a multi-component cascade reaction, and the target product (**4i**) was isolated with an 88% yield. Further tests showed that an internal alkene, such as cyclohexene (**4j**) was a feasible substrate, highlighting the broad applicability of the current strategy. Chained unactivated alkenes, such as allyl phenyl ether and allyl benzoate, were suitable substrates, all producing the desired products (**4k**, **4L**) in high yields.

**SCHEME 3:**
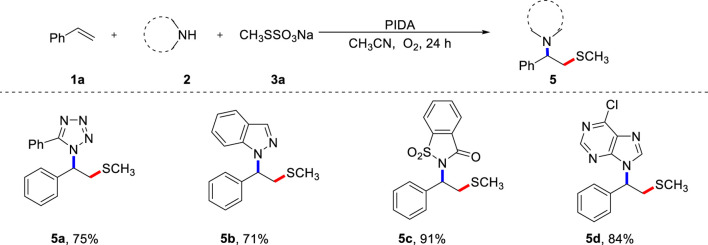
Scope of nitrogen nucleophiles **(A)**. **(A)** Reaction conditions: **1a** (0.2 mmol), **2** (0.4 mmol), **3a** (0.4 mmol), PIDA (0.4 mmol), CH_3_CN (2.0 mL) under O_2_, heated at 80 °C for 24 h, isolated yield.

To further investigate the synthetic application of the current protocol, various azaheterocycles were evaluated, and the results are shown in Scheme 3. Due to their prominent bioelectronic isosteric nature, tetrazoles are particularly significant in the development of pharmaceutical candidates. Therefore, it is important to further investigate this multi-component reaction strategy to diversify and functionalize tetrazoles. To our delight, 5-phenyltetrazole proved to be a feasible azolating reagent, resulting in the desired product **5a**. Importantly, 1H-indazole was effective in the current reaction system and gave the good result (**5b**). Saccharin was also compatible with this procedure, resulting in the anticipated product **5c** in excellent yield. 6-Chloropurine could also be tolerated, providing a concise pathway for *β*-thiomethylated N-alkyl purine **5d**.

**SCHEME 4:**
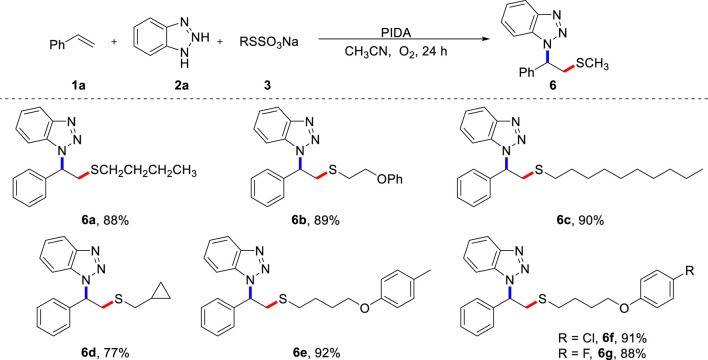
Scope of Bunte salts **(A)**. **(A)** Reaction conditions: **1a** (0.2 mmol), **2a** (0.4 mmol), **3** (0.4 mmol), PIDA (0.4 mmol), CH_3_CN (2.0 mL) under O_2_, heated at 80 °C for 24 h, isolated yield.

To demonstrate the potential application of this protocol, we prepared and examined various alkyl Bunte salts as thiolation reagents in the metal-free aminothiolation of alkenes (Scheme 4). Significant functional groups, such as alkyl (**6a**, **6c**), ether (**6b**), cyclopropyl (**6d**), methyl (**6e**), and halogen (**6f**, **6g**) were well accommodated, providing a good platform for additional diversification through classical synthetic transformations. Importantly, butanethiol and dimethyl disulfide did not participate in this transformation, which indicates the powerful reactivity of alkyl Bunte salts. It should be emphasized that, in contrast to the use of smelly and unstable dialkyl disulfides for the construction of alkyl sulfides, Bunte salts are odorless reagents that are bench-stable and easy to prepare. This is beneficial for diverse synthesis.

**SCHEME 5:**
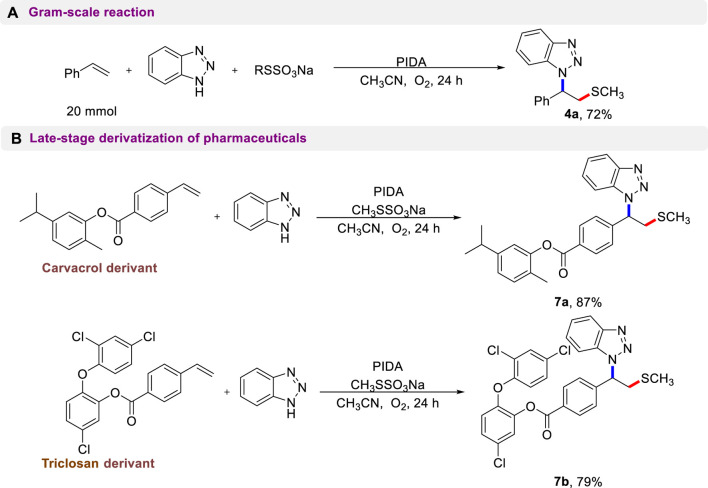
Synthetic application.

To further demonstrate the potential applicability of this protocol, a scale-up preparation of **4a** and late-stage functionalization of complex compounds were conducted. As shown in Scheme 5A, there was no discernible effect on the 20 mmol-scale reaction, and compound **4a** was still isolated in a 72% yield. Complex alkene molecules could undergo a smooth aminothiolation reaction under standard reaction conditions (Scheme 5B). Remarkably, pharmaceutical derivatives such as carvacrol and triclosan were found to be accommodating and provided the anticipated products (**7a**, **7b**) in excellent yields. This result demonstrates the powerful compatibility of this metal-free reaction system and its potential for use in modifying the structure of various bioactive molecules.

**SCHEME 6:**
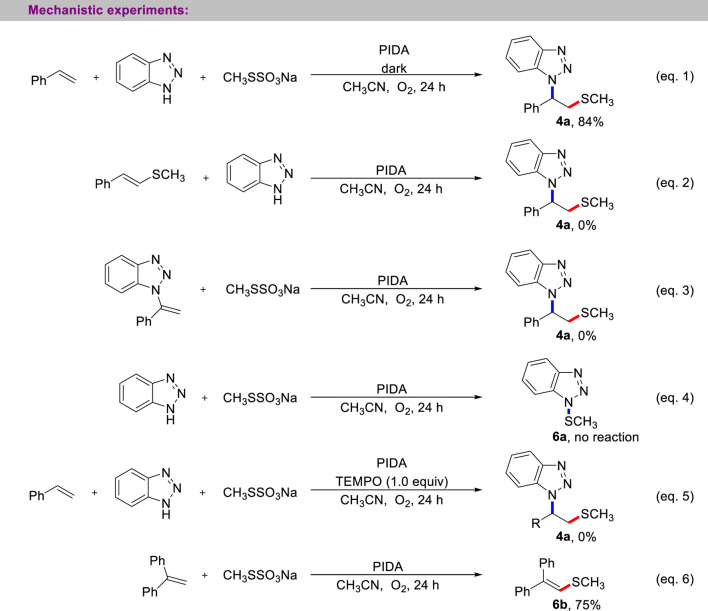
Mechanistic experiments.

Several carefully designed control experiments were performed to gain a detailed insight into the reaction mechanism (Scheme 6). First, the light shielding experiment showed that the current multi-component cascade reaction only requires the thermodynamic excitation process (entry 1). Next, to verify the possible competent intermediate in the current three-component reaction, we prepared the vinylmethyl sulfide and *N*-vinyl benzotriazole, whether the reaction between vinylmethyl sulfide and benzotriazole or the reaction between *N*-vinyl benzotriazole with CH_3_SSO_3_Na, the desired product 4a was not observed (entries 2, 3). Subsequently, the treatment of benzotriazole with CH_3_SSO_3_Na did not provide the *N*-SCH_3_ benzotriazole (entry 4). These experimental results suggest that the Michael addition and electrophilic aminothiolation of alkenes with sulfenamides were not involved in current aminothiolation process. Finally, the possible radical mechanism of the reaction was studied. When 1.0 equivalent of TEMPO was added to the reaction system, the reaction was completely suppressed (entry 5). Wheras 1,1-diphenylethylene was used as a free radical trapping agent, the product of methyl thiogroup addition was successfully captured (entry 6).

**SCHEME 7:**
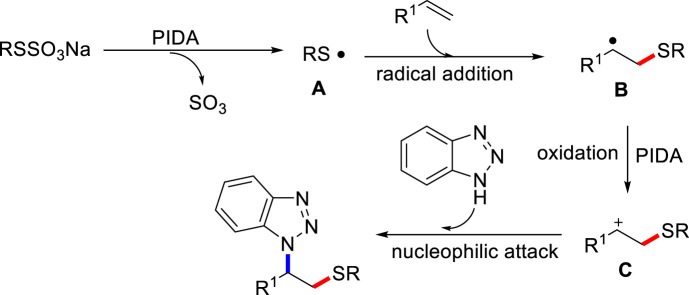
Proposed mechanism.

Based on the aforementioned experiments and related literature reports, we proposed a possible mechanism for this aminothiolation of alkenes process in Scheme 7. Firstly, Bunte salt is activated to discharge sulfur trioxide gas to produce alkyl sulfur radical in the presence of PIDA (Prabhakar et al., 2022). Then, electrophilic alkyl sulfur free radicals add to double bonds of olefin to obtain alkyl free radical intermediate **B**.

The resulted radical intermediate **B** could be oxidized to carbon cation **C** in the presence of PIDA. Finally, the active alkyl cation **C** undergoes nucleophilic attack with aza-heterocycle to give the final product.

## Conclusion

In summary, we have developed a heat-induced three-component coupling of alkenes, aza-heterocycles and Bunte salts for the preparation of β-amino sulfides. The outstanding advantage of this strategy is that it avoids the use of expensive transition metals and additional additives. In addition, a simple reaction system enables readily scaled-up reactions and late-stage modification of drug molecules, which provides an efficient and practical method for the development of thiomethylation drugs. Remarkably, this metal-free aminothiolation of alkenes makes use of readily available and cheap starting materials and shows uniform regioselectivity in all cases.

## Data Availability

The original contributions presented in the study are included in the article/Supplementary Material, further inquiries can be directed to the corresponding author.
