# Supplementary material for: Aminothiolation of alkenes with azoles and Bunte salts
Source: Front Chem. 2024 Jan 22;11:1328441. doi: 10.3389/fchem.2023.1328441 (PMC10845345; doi:10.3389/fchem.2023.1328441)
Supplement: Supplementary file 1 [file DataSheet1.pdf]

Aminothiolation of alkenes with azoles and Bunte salts

Bingqing Ouyang,[1†] Xing Chai,[2†] Zhe Li,[1] Chunling Zhang,[1] Xingmei Liu[1]*

[1]Department of Pharmacy, The Second Norman Bethune Hospital of Jilin University, Changchun130000, China

[2]Outpatient Department, The Second Norman Bethune Hospital of Jilin University, Changchun130000, China

***Correspondence:** Xingmei Liu, 13844973027@163.com

Table of Contents

(1) General considerations, experimental data……….S2-S16

(2) ^{1}H, ^{13}C and ^{19}F NMR spectra of products……….S17-S42

(3) HRMS spectra of products……………………….S43-S55

General Information

All other reagents were purchased from TCI, Sigma-Aldrich, Alfa Aesar, Acros, and Meryer and used without further purification. ^{1}H NMR (500 MHz), ^{13}C NMR (125 MHz) and ^{19}F NMR (470 MHz) spectra were recorded in $CDCl_3$ and DMSO-D6 solutions using a Burker AVANCE 500 spectrometer. High-resolution mass spectra were recorded on an ESI-Q-TOF mass spectrometer. Analysis of crude reaction mixture was done on the Varian 4000 GC/MS and 1200 LC. All reactions were conducted using standard Schlenk techniques. Column chromatography was performed using EM silica gel 60 (300−400 m).

General Experimental Procedures

General Procedure of aminothiolation of alkenes with azoles and Bunte salts:

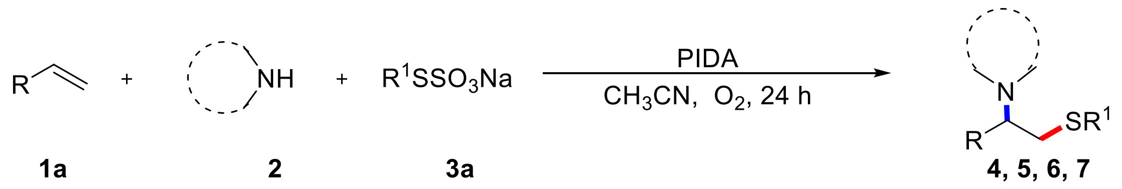

A 25 mL Schlenk tube equipped with a stir bar was charged with substituted alkenes (0.2 mmol), azoles (0.4 mmol), Bunte salts (0.4 mmol), PIDA (0.4 mmol) and CH_3CN (2.0 mL). The tube was fitted with a rubber septum, and then it was evacuated and refilled with O_2 three times. The reaction mixture was stirred at 80 °C for 24 h. After cooling down, the reaction mixture was diluted with 10 mL of ethyl ether and filtered with silica-gel powder, and concentrated under reduced pressure. The residue was then purified by flash chromatography on silica gel to provide the corresponding product.

Large-scale reaction:

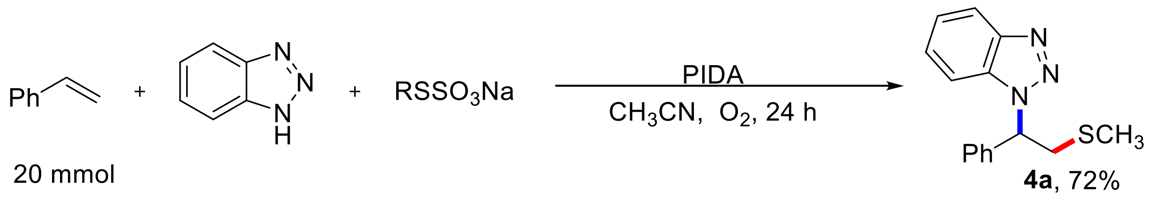

A 500 mL Schlenk tube equipped with a stir bar was charged with styrene (20 mmol), 1H-benzotriazole (40 mmol), CH_3SSO_3Na (40 mmol), PIDA (40 mmol) and CH_3CN (200 mL). The tube was fitted with a rubber septum, and then it was evacuated and refilled with O_2 three times. The reaction mixture was stirred at 80 °C for 24 h. After cooling down, the reaction mixture was diluted with 10 mL of ethyl ether and filtered with silica-gel powder, and concentrated under reduced pressure. The residue was then purified by flash chromatography on silica gel to provide the corresponding product.

Mechanistic Studies

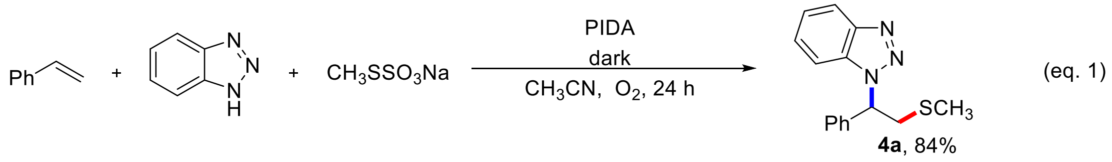

A 25 mL Schlenk tube equipped with a stir bar was charged with styrene (0.2 mmol), 1H-benzotriazole (0.4 mmol), CH_3SSO_3Na (0.4 mmol), PIDA (0.4 mmol) and 2.0 mL CH_3CN. The tube was fitted with a rubber septum, and then it was evacuated and refilled with O_2 three times, and packed with foil. The reaction mixture was stirred at 80 ℃ for 24 h. After the reaction mixture was cooled to room temperature and the reaction was filtered through a pad of Celite and diluted with ethyl acetate (10 mL), and the yield of **4a** was isolated in 84%.

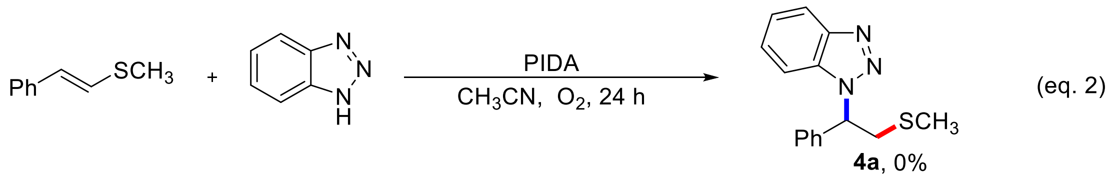

A 25 mL Schlenk tube equipped with a stir bar was charged with (E)-methyl(styryl)sulfane (0.2 mmol), 1H-benzotriazole (0.4 mmol), PIDA (0.4 mmol) and 2.0 mL CH_3CN. The tube was fitted with a rubber septum, and then it was evacuated and refilled with O_2 three times, and packed with foil. The reaction mixture was stirred at 80 ℃ for 24 h. After the reaction mixture was cooled to room temperature and the reaction was filtered through a pad of Celite and diluted with ethyl acetate (10 mL), and none of **4a** was detected by GC-MS.

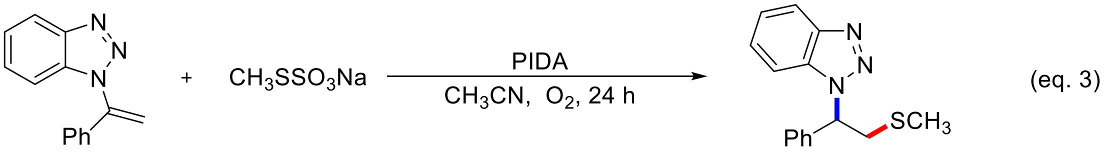

4a, 0%

A 25 mL Schlenk tube equipped with a stir bar was charged with 1-(1-phenylvinyl)-1H-benzo[d][1,2,3]triazole (0.2 mmol), CH_3SSO_3Na (0.4 mmol), PIDA (0.4 mmol) and 2.0 mL CH_3CN. The tube was fitted with a rubber septum, and then it was evacuated and refilled with O_2 three times, and packed with foil. The reaction mixture was stirred at 80 ℃ for 24 h. After the reaction mixture was cooled to room temperature and the reaction was filtered through a pad of Celite and diluted with ethyl acetate (10 mL), and none of **4a** was detected by GC-MS.

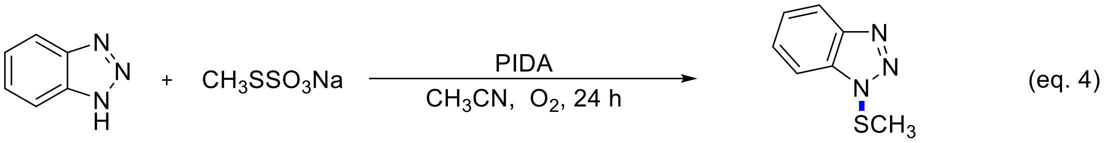

6a, no reaction

A 25 mL Schlenk tube equipped with a stir bar was charged with styrene (0.2 mmol), 1H-benzotriazole (0.4 mmol), CH_3SSO_3Na (0.4 mmol), PIDA (0.4 mmol) and 2.0 mL CH_3CN. The tube was fitted with a rubber septum, and then it was evacuated and refilled with O_2 three times, and packed with foil. The reaction mixture was stirred at 80 ℃ for 24 h. After the reaction mixture was cooled to room temperature and the reaction was filtered through a pad of Celite and diluted with ethyl acetate (10 mL), and none of **6a** was detected by GC-MS.

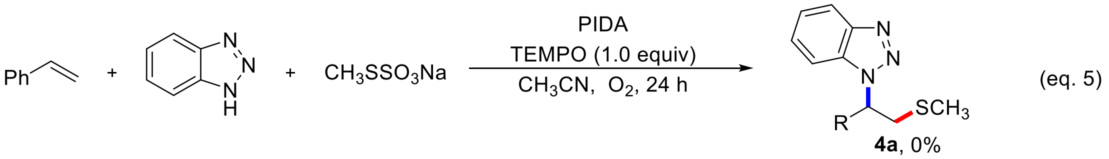

4a, 0%

A 25 mL Schlenk tube equipped with a stir bar was charged with styrene (0.2 mmol), 1H-benzotriazole (0.4 mmol), CH_3SSO_3Na (0.4 mmol), TEMPO (0.2 mmol), PIDA (0.4 mmol) and 2.0 mL CH_3CN. The tube was fitted with a rubber septum, and then it was evacuated and refilled with O_2 three times, and packed with foil. The reaction mixture was stirred at 80 ℃ for 24 h. After the reaction mixture was cooled to room temperature and the reaction was filtered through a pad of Celite and diluted with ethyl acetate (10 mL), and none of **4a** was observed on the TLC.

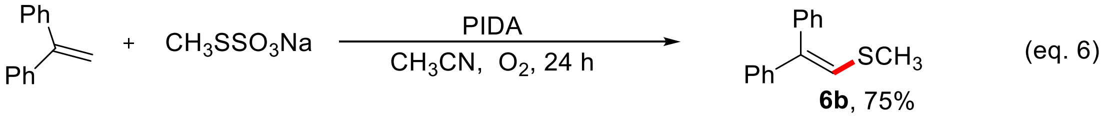

6b, 75%

A 25 mL Schlenk tube equipped with a stir bar was charged with 1,1-diphenylethylene (0.2 mmol), CH_3SSO_3Na (0.4 mmol), PIDA (0.4 mmol) and 2.0 mL CH_3CN. The tube was fitted with a rubber septum, and then it was evacuated and refilled with O_2 three times, and packed with foil. The reaction mixture was stirred at 80 ℃ for 24 h. After the reaction mixture was cooled to room temperature and the reaction was filtered through a pad of Celite and diluted with ethyl acetate (10 mL), and the corresponding product **6b** was isolated in 75%.
The structure of **6b** was in agreement with the literature report: Gao, X., Pan, X., Gao, J., Jiang, H., Yuan, G., and Li, Y. *Org. Lett.* 2015, *17*, 1038-1041.

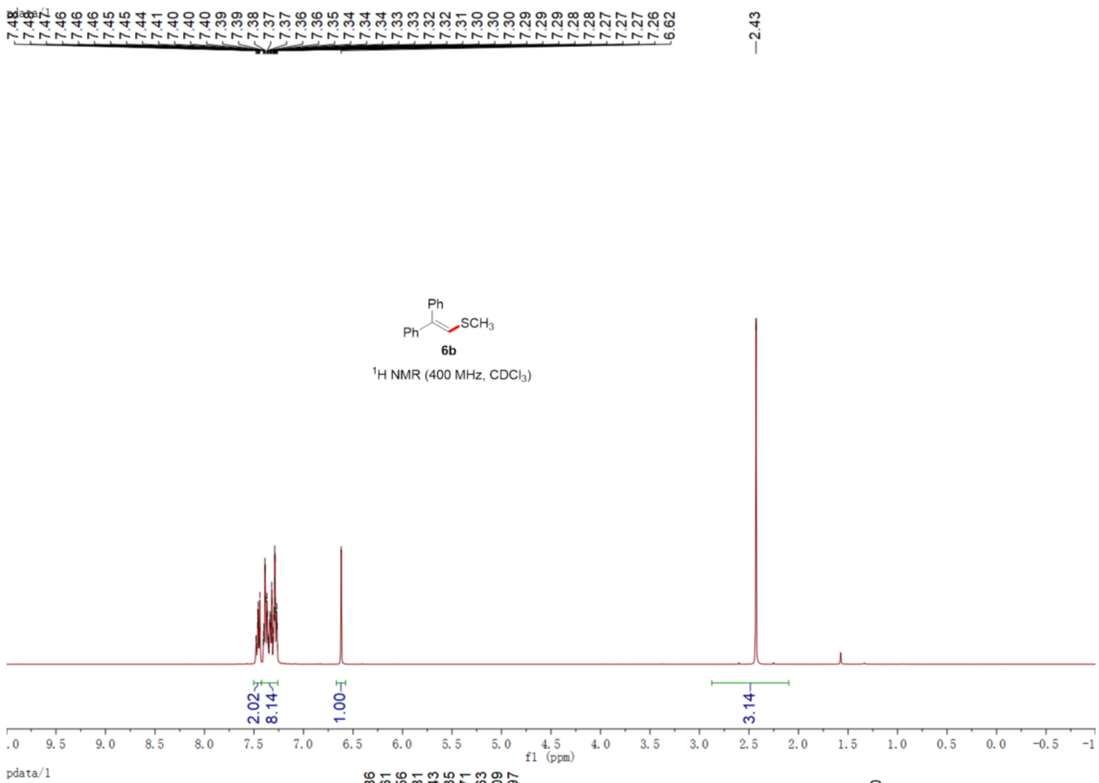
pdata/1
7.48 7.47 7.47 7.46 7.46 7.45 7.45 7.44 7.41 7.40 7.40 7.40 7.39 7.39 7.38 7.37 7.37 7.36 7.36 7.35 7.34 7.34 7.34 7.33 7.33 7.32 7.32 7.31 7.30 7.30 7.30 7.29 7.29 7.29 7.28 7.28 7.27 7.27 7.27 7.26 6.62
2.43
Ph
Ph
SCH3
6b
1H NMR (400 MHz, CDCl3)
2.02
8.14
1.00
3.14
f1 (ppm)

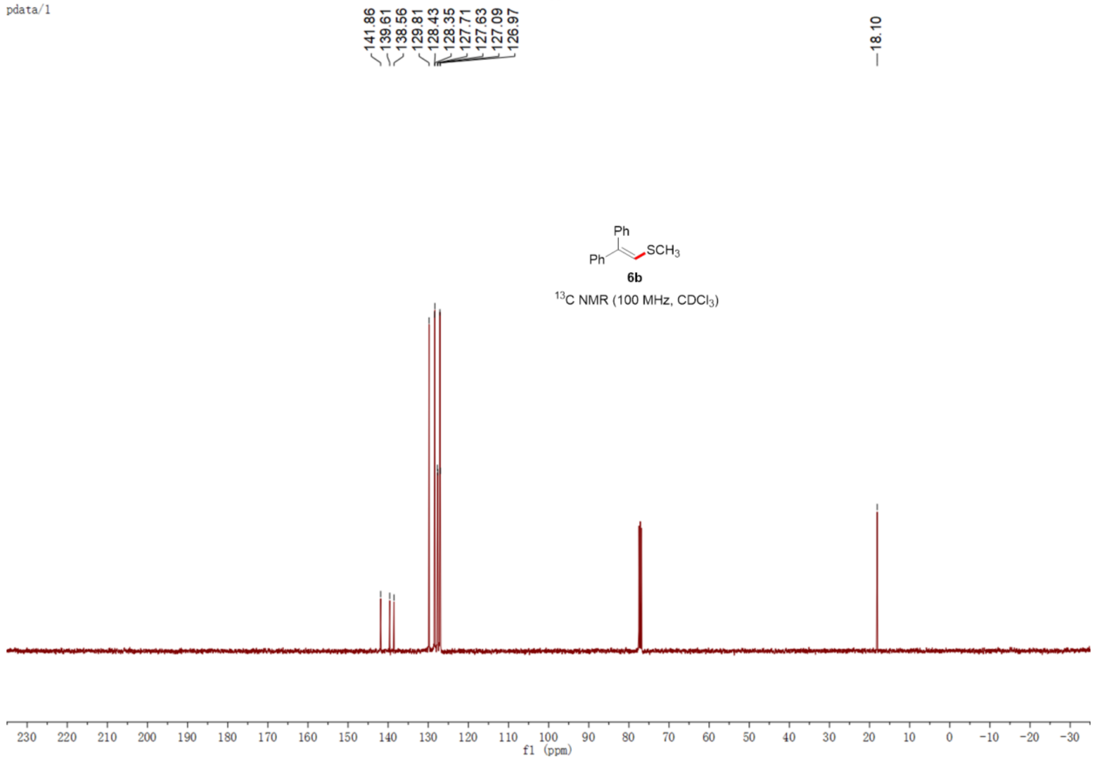
pdata/1
141.86 139.61 138.56 129.81 128.43 128.35 127.71 127.63 127.09 126.97
18.10
Ph
Ph
SCH3
6b
13C NMR (100 MHz, CDCl3)
f1 (ppm)

Characterization of Products in Details :

1-(2-(methylthio)-1-phenylethyl)-1H-benzo[d][1,2,3]triazole

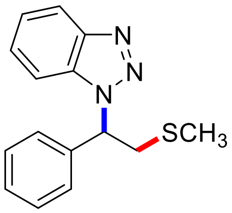

4a

Following the general procedure, using (petroleum ether : EtOAc = 9 : 1) as the eluant afforded a yellow solid (46.8 mg, 87% yield), Mp = 81-82℃. **^{1}H NMR** (400 MHz, $CDCl_3$): δ 8.09 (d, *J* = 8.3 Hz, 1H), 7.46-7.40 (m, 4H), 7.38-7.29 (m, 4H), 5.95 (dd, *J* = 8.7, 6.3 Hz, 1H), 3.93 (dd, *J* = 13.9, 8.7 Hz, 1H), 3.57 (dd, *J* = 13.9, 6.3 Hz, 1H), 2.02 (s, 3H). **^{13}C NMR** (100 MHz, $CDCl_3$): δ 138.16, 133.23, 129.06, 128.81, 127.45, 127.09, 124.10, 120.11, 109.71, 63.82, 39.34, 16.42. **HRMS** (ESI): calcd for $C_{15}H_{15}N_3Na$ S $[M + Na]^+$ 292.0884, found 292.0893.

1-(2-(methylthio)-1-(p-tolyl)ethyl)-1H-benzo[d][1,2,3]triazole

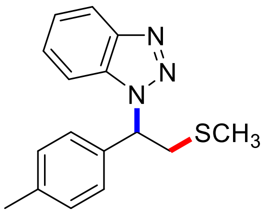

4b

Following the general procedure, using (petroleum ether : EtOAc = 9 : 1) as the eluant afforded a yellow liquid (49.8 mg, 88% yield). **^{1}H NMR** (400 MHz, $CDCl_3$): δ 8.07 (d, *J* = 8.3 Hz, 1H), 7.47-7.40 (m, 2H), 7.36-7.32 (m, 3H), 7.16 (d, *J* = 7.9 Hz, 2H), 5.94 (dd, *J* = 8.7, 6.3 Hz, 1H), 3.91 (dd, *J* = 13.9, 8.7 Hz, 1H), 3.56 (dd, *J* = 13.9, 6.3 Hz, 1H), 2.32 (s, 3H), 2.03 (s, 3H). **^{13}C NMR** (100 MHz, $CDCl_3$): δ 146.02, 138.72, 135.10, 133.14, 129.73, 127.48, 127.02, 124.20, 119.96, 109.88, 63.54, 39.34, 21.18, 16.47. **HRMS** (ESI): calcd for $C_{16}H_{17}N_3NaS$ $[M + Na]^+$ 306.1041, found 306.1051.

1-(1-(4-(tert-butyl)phenyl)-2-(methylthio)ethyl)-1H-benzo[d][1,2,3]triazole

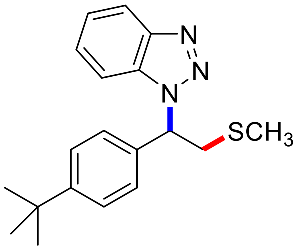

4c

Following the general procedure, using (petroleum ether : EtOAc = 9 : 1) as the eluant afforded a white solid (58.5 mg, 90% yield), Mp = 121-122℃. **^{1}H NMR** (400 MHz, $CDCl_3$): δ 8.09 (d, *J* = 8.3 Hz, 1H), 7.49-7.42(m, 2H), 7.40-7.34 (m, 5H), 5.94 (dd, *J* = 9.0, 6.0 Hz, 1H), 3.93 (dd, *J* = 14.0, 9.0 Hz, 1H), 3.55 (dd, *J* = 14.1, 6.1 Hz, 1H), 2.03 (s, 3H), 1.30 (s, 9H). **^{13}C NMR** (100 MHz, $CDCl_3$): δ 151.86, 146.14, 135.11, 133.21, 127.39, 126.77, 125.97, 124.08, 120.09, 109.77, 63.48, 39.35, 34.65, 31.29, 16.42. **HRMS** (ESI): calcd for $C_{19}H_{23}N_3NaS$ $[M + Na]^+$ 348.1510, found 348.1517.

1-(1-(4-chlorophenyl)-2-(methylthio)ethyl)-1H-benzo[d][1,2,3]triazole

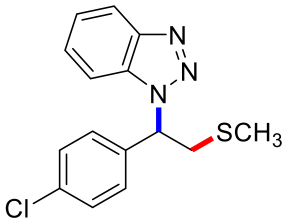

4d

Following the general procedure, using (petroleum ether : EtOAc = 9 : 1) as the eluant afforded a yellow solid (51.5 mg, 85% yield), Mp = 70-71℃. **^{1}H NMR** (400 MHz, $CDCl_3$): δ 8.05 (d, *J* = 8.3 Hz, 1H), 7.44-7.27 (m, 7H), 5.92 (dd, *J* = 8.5, 6.5 Hz, 1H), 3.85 (dd, *J* = 13.9, 8.5 Hz, 1H), 3.51 (dd, *J* = 13.9, 6.5 Hz, 1H), 1.99 (s, 3H). **^{13}C NMR** (100 MHz, $CDCl_3$): δ 146.12, 136.66, 134.70, 133.08, 129.22, 128.57, 127.66, 124.29, 120.11, 109.59, 62.92, 39.30, 16.43. **HRMS** (ESI): calcd for $C_{15}H_{14}N_3NaSCl$ $[M + Na]^+$ 326.0495, found 326.0503.

1-(1-(4-bromophenyl)-2-(methylthio)ethyl)-1H-benzo[d][1,2,3]triazole

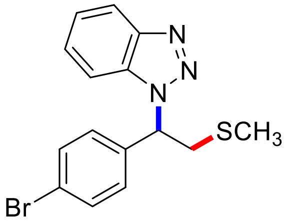

4e

Following the general procedure, using (petroleum ether : EtOAc = 9 : 1) as the eluant afforded a yellow liquid (54.8 mg, 79% yield). **^{1}H NMR** (400 MHz, $CDCl_3$): δ 8.09 (d, *J* = 8.3 Hz, 1H), 7.50-7.42 (m, 4H), 7.39-7.35 (m, 1H), 7.34-7.30 (m, 2H), 5.91 (dd, *J* = 8.5, 6.5 Hz, 1H), 3.86 (d, *J* = 8.5 Hz, 1H), 3.54 (dd, *J* = 13.9, 6.5 Hz, 1H), 2.03 (s, 3H). **^{13}C NMR** (100 MHz, $CDCl_3$): δ 146.14, 137.06, 133.13, 132.24, 128.83, 127.74, 124.38, 122.99, 120.17, 109.55, 63.05, 39.38, 16.66. **HRMS** (ESI): calcd for $C_{15}H_{14}N_3NaSBr$ $[M + Na]^+$ 369.9990, found 370.0000.

1-(1-(4-fluorophenyl)-2-(methylthio)ethyl)-1H-benzo[d][1,2,3]triazole

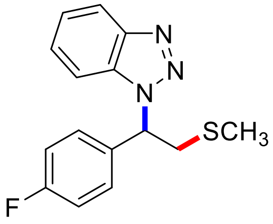

4f

Following the general procedure, using (petroleum ether : EtOAc = 9 : 1) as the eluant afforded a yellow solid (40.7 mg, 71% yield), Mp = 71-72℃. **^{1}H NMR** (400 MHz, $CDCl_3$): δ 8.08 (d, *J* = 8.3 Hz, 1H), 7.46-7.42 (m, 4H), 7.38-7.34 (m, 1H), 7.06-7.01 (m, 2H), 5.93 (dd, *J* = 8.5, 6.5 Hz, 1H), 3.88 (dd, *J* = 13.9, 8.5 Hz, 1H), 3.54 (dd, *J* = 13.9, 6.5 Hz, 1H), 2.01 (s, 3H). **^{13}C NMR** (100 MHz, $CDCl_3$): δ 162.77 (d, *J* = 248.2 Hz), 146.10, 133.98 (d, *J* = 3.3 Hz), 133.05, 129.00 (d, *J* = 8.4 Hz), 127.63, 124.28, 120.13, 116.03 (d, *J* = 21.8 Hz), 109.59, 62.94, 39.50, 16.48. **^{19}F NMR** (375 MHz, $CDCl_3$) δ -112.54 (1F); **HRMS** (ESI): calcd for $C_{15}H_{14}N_3NaSF$ $[M + Na]^+$ 310.0790, found 310.0791.

4-(1-(1H-benzo[d][1,2,3]triazol-1-yl)-2-(methylthio)ethyl)phenyl acetate

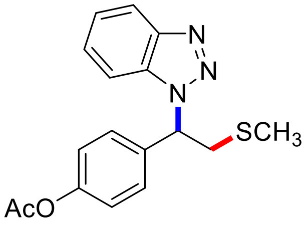

4g

Following the general procedure, using (petroleum ether : EtOAc = 9 : 1) as the eluant afforded a yellow liquid (54.9 mg, 84% yield). **^{1}H NMR** (400 MHz, $CDCl_3$): δ 8.11 (d, *J* = 8.3 Hz, 1H), 7.49-7.43(m, 4H), 7.41-7.37 (m, 1H), 7.11 (d, *J* = 8.6 Hz, 2H), 5.93 (dd, *J* = 8.9, 6.1 Hz, 1H), 3.92 (dd, *J* = 14.0, 8.9 Hz, 1H), 3.54 (dd, *J* = 14.0, 6.1 Hz, 1H), 2.31 (s, 3H), 2.02 (s, 3H). **^{13}C NMR** (100 MHz, $CDCl_3$): δ 169.22, 150.89, 146.17, 135.68, 133.13, 128.24, 127.57, 124.19, 122.21, 120.21, 109.52, 63.24, 39.49, 21.14, 16.44. **HRMS** (ESI): calcd for $C_{17}H_{17}N_3O_2NaS$ $[M + Na]^+$ 350.0939, found 350.0947.

1-(1-cyclohexyl-2-(methylthio)ethyl)-1H-benzo[d][1,2,3]triazole

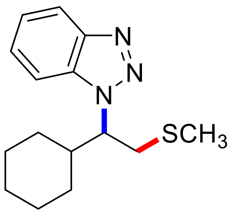

4h

Following the general procedure, using (petroleum ether : EtOAc = 9 : 1) as the eluant afforded a yellow solid (40.7 mg, 74% yield), Mp = 67-68℃. **^{1}H NMR** (400 MHz, $CDCl_3$): δ 8.09 (d, J = 8.4 Hz, 1H), 7.60 (d, J = 8.3 Hz, 1H), 7.53 (t, J = 7.5 Hz, 1H), 7.40 (t, J = 7.6 Hz, 1H), 4.91 (dd, J = 14.3, 6.9 Hz, 1H), 4.72 (dd, J = 14.3, 8.0 Hz, 1H), 3.12 (td, J = 7.5, 3.3 Hz, 1H), 1.97-1.94 (m, 1H), 1.89 (s, 3H), 1.84-1.76 (m, 2H), 1.72-1.68 (m, 2H), 1.56-1.48 (m, 2H), 1.28-1.18 (m, 4H). **^{13}C NMR** (100 MHz, $CDCl_3$): δ 146.03, 133.69, 127.43, 123.96, 120.05, 109.55, 54.97, 50.74, 40.20, 30.69, 28.62, 26.30, 26.27, 26.24, 16.01. **HRMS** (ESI): calcd for $C_{15}H_{21}N_3NaS$ $[M + Na]^+$ 298.1354, found 298.1362.

1-(2-(methylthio)-1-(naphthalen-2-yl)ethyl)-1H-benzo[d][1,2,3]triazole

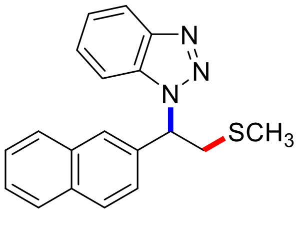

4i

Following the general procedure, using (petroleum ether : EtOAc = 9 : 1) as the eluant afforded a yellow liquid (56.1 mg, 88% yield). **^{1}H NMR** (400 MHz, $CDCl_3$): δ 8.12 (d, J = 8.0 Hz, 1H), 7.94 (s, 1H), 7.87-7.81 (m, 3H), 7.55-7.47 (m, 4H), 7.41 (t, J = 7.5 Hz, 1H), 7.36 (t, J = 7.5 Hz, 1H), 6.14 (dd, J = 8.6, 6.3 Hz, 1H), 4.05 (dd, J = 13.9, 8.6 Hz, 1H), 3.70 (dd, J = 13.9, 6.3 Hz, 1H), 2.08 (s, 3H). **^{13}C NMR** (100 MHz, $CDCl_3$): δ 146.19, 135.37, 133.29, 133.20, 133.14, 129.20, 128.19, 127.82, 127.58, 126.80, 126.78, 126.38, 124.36, 124.27, 120.10, 109.79, 63.89, 39.44, 16.75. **HRMS** (ESI): calcd for $C_{19}H_{17}N_3NaS$ $[M + Na]^+$ 342.1041, found 342.1050.

1-(2-(methylthio)cyclohexyl)-1H-benzo[d][1,2,3]triazole

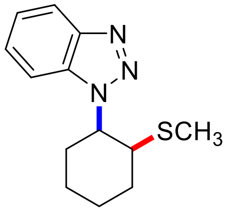

4j

Following the general procedure, using (petroleum ether : EtOAc = 9 : 1) as the eluant afforded a yellow liquid (38.0 mg, 77% yield). **^{1}H NMR** (400 MHz, $CDCl_3$): δ 8.08 (d, J = 8.0 Hz, 1H), 7.62 (d, J = 8.3 Hz, 1H), 7.50 (t, J = 7.6 Hz, 1H), 7.38 (t, J = 7.0 Hz, 1H), 4.53 (td, J = 11.5, 4.1 Hz, 1H), 3.33 (td, J = 11.4, 4.0 Hz, 1H), 2.40-2.36 (m, 2H), 2.21-2.16 (m, 1H), 2.01-1.93 (m, 2H), 1.61-1.53 (m, 6H). **^{13}C NMR** (100 MHz, $CDCl_3$): δ 145.91, 133.51, 127.24, 124.04, 120.09, 109.94, 63.80, 50.50, 33.71, 26.18, 25.55, 14.02. **HRMS** (ESI): calcd for $C_{13}H_{17}N_3NaS$ $[M + Na]^+$ 270.1041, found 270.1052.

1-(1-(methylthio)-3-phenoxypropan-2-yl)-1H-benzo[d][1,2,3]triazole

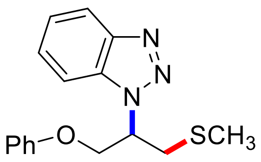

4k

Following the general procedure, using (petroleum ether : EtOAc = 9 : 1) as the eluant afforded a yellow liquid (49.0 mg, 82% yield). **^{1}H NMR** (400 MHz, $CDCl_3$): δ 8.08 (d, *J* = 8.3 Hz, 1H), 7.58 (d, *J* = 8.3 Hz, 1H), 7.45 (t, *J* = 7.5 Hz, 1H), 7.37 (t, *J* = 7.5 Hz, 1H), 7.30 (t, *J* = 8.0 Hz, 2H), 7.00 (t, *J* = 7.4 Hz, 1H), 6.90 (d, *J* = 8.0 Hz, 2H), 5.13 (dd, *J* = 14.4, 6.7 Hz, 1H), 4.92 (dd, *J* = 14.4, 7.0 Hz, 1H), 4.18-4.10 (m, 2H), 3.61 (qd, *J* = 6.7, 4.5 Hz, 1H), 2.15 (s, 3H). **^{13}C NMR** (100 MHz, $CDCl_3$): δ 158.10, 145.83, 133.66, 129.67, 127.61, 124.08, 121.56, 120.03, 114.67, 109.57, 68.16, 48.96, 46.53, 14.86. **HRMS** (ESI): calcd for $C_{16}H_{17}N_3ONaS$ $[M + Na]^+$ 322.0990, found 322.0999.

2-(1H-benzo[d][1,2,3]triazol-1-yl)-3-(methylthio)propyl benzoate

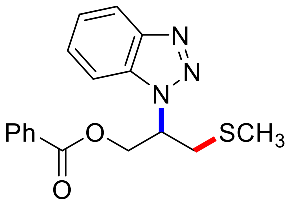

4l

Following the general procedure, using (petroleum ether : EtOAc = 9 : 1) as the eluant afforded a yellow liquid (54.9 mg, 84% yield). **^{1}H NMR** (400 MHz, $CDCl_3$): δ 8.06 (d, *J* = 8.3 Hz, 1H), 7.97 (d, *J* = 7.5 Hz, 2H), 7.59 (t, *J* = 7.3 Hz, 2H), 7.50-7.44 (m, 3H), 7.37 (t, *J* = 7.6 Hz, 1H), 5.02 (dd, *J* = 14.5, 6.9 Hz, 1H), 4.89 (dd, *J* = 14.5, 7.1 Hz, 1H), 4.58 (dd, *J* = 11.7, 4.6 Hz, 1H), 4.48 (dd, *J* = 11.7, 7.1 Hz, 1H), 3.68 (qd, *J* = 7.1, 4.5 Hz, 1H), 2.16 (s, 3H). **^{13}C NMR** (100 MHz, $CDCl_3$): δ 166.11, 133.41, 129.79, 129.69, 129.46, 128.57, 128.47, 127.73, 124.13, 120.16, 109.36, 64.53, 49.44, 45.95, 14.63. **HRMS** (ESI): calcd for $C_{17}H_{17}N_3O_2NaS$ $[M + Na]^+$ 350.0939, found 350.0949.

1-(2-(methylthio)-1-phenylethyl)-5-phenyl-1H-tetrazole

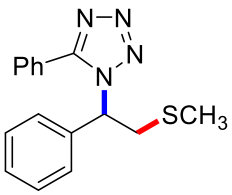

5a

Following the general procedure, using (petroleum ether : EtOAc = 9 : 1) as the eluant afforded a yellow solid (44.4

mg, 75% yield), Mp = 75-76℃. ^{1}**H NMR** (400 MHz, $CDCl_3$): δ 8.24 (dd, *J* = 7.9, 1.8 Hz, 2H), 7.57-7.46 (m, 5H), 7.44-7.36 (m, 3H), 6.15 (dd, *J* = 9.5, 5.9 Hz, 1H), 3.76 (dd, *J* = 14.2, 9.5 Hz, 1H), 3.42 (dd, *J* = 14.2, 5.9 Hz, 1H), 2.09 (s, 3H). ^{13}**C NMR** (100 MHz, $CDCl_3$): δ 165.19, 136.80, 130.45, 129.28, 129.14, 128.97, 127.56, 127.38, 127.03, 67.98, 39.33, 16.04. **HRMS** (ESI): calcd for $C_{16}H_{16}N_4NaS$ $[M + Na]^+$ 319.0993, found 319.1002.

1-(2-(methylthio)-1-phenylethyl)-1H-indazole

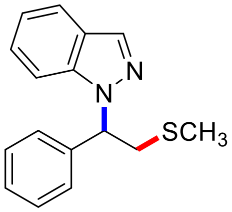

5b

Following the general procedure, using (petroleum ether : EtOAc = 9 : 1) as the eluant afforded a yellow liquid (38.1 mg, 71% yield). ^{1}**H NMR** (400 MHz, $CDCl_3$): δ δ 8.17 (s, 1H), 7.79 (d, *J* = 8.1 Hz, 1H), 7.49-7.29 (m, 7H), 7.19 (t, *J* = 7.4 Hz, 1H), 5.81 (dd, *J* = 9.2, 5.6 Hz, 1H), 3.90 (dd, *J* = 13.7, 9.2 Hz, 1H), 3.45 (dd, *J* = 13.7, 5.6 Hz, 1H), 2.04 (s, 3H). ^{13}**C NMR** (100 MHz, $CDCl_3$): δ 140.07, 140.02, 133.54, 128.81, 128.19, 126.97, 126.47, 124.37, 121.24, 120.96, 109.19, 62.86, 39.60, 16.40. **HRMS** (ESI): calcd for $C_{16}H_{16}N_2NaS$ $[M + Na]^+$ 291.0932, found 291.0942.

2-(2-(methylthio)-1-phenylethyl)benzo[d]isothiazol-3(2H)-one 1,1-dioxide

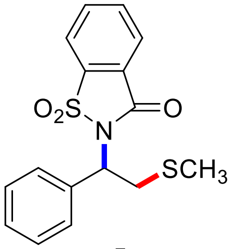

5c

Following the general procedure, using (petroleum ether : EtOAc = 9 : 1) as the eluant afforded a yellow solid (60.6 mg, 91% yield), Mp = 108-109℃. ^{1}**H NMR** (400 MHz, $CDCl_3$): δ 8.05 (d, *J* = 6.8 Hz, 1H), 7.92-7.80 (m, 3H), 7.67-7.63 (m, 2H), 7.44-7.36 (m, 3H), 5.40 (dd, *J* = 8.9, 7.1 Hz, 1H), 3.79 (dd, *J* = 13.9, 8.9 Hz, 1H), 3.46 (dd, *J* = 13.9, 7.1 Hz, 1H), 2.21 (s, 3H). ^{13}**C NMR** (100 MHz, $CDCl_3$): δ 158.93, 137.47, 136.62, 134.80, 134.37, 128.84, 128.62, 127.26, 125.26, 120.89, 56.38, 35.77, 15.79. **HRMS** (ESI): calcd for $C_{16}H_{15}NO_3NaS_2$ $[M + Na]^+$ 356.0391, found 356.0399.

6-chloro-9-(2-(methylthio)-1-phenylethyl)-9H-purine

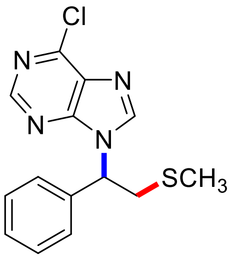

5d

Following the general procedure, using (petroleum ether : EtOAc = 9 : 1) as the eluant afforded a yellow liquid (51.1 mg, 84% yield). **^{1}H NMR** (400 MHz, $CDCl_3$): δ 8.79 (s, 1H), 8.23 (s, 1H), 7.46-7.36 (m, 5H), 5.95 (dd, *J* = 9.5, 5.9 Hz, 1H), 3.71 (dd, *J* = 14.0, 9.5 Hz, 1H), 3.44 (dd, *J* = 14.1, 5.9 Hz, 1H), 2.11 (s, 3H). **^{13}C NMR** (100 MHz, $CDCl_3$): δ 152.02, 151.22, 144.15, 137.27, 131.77, 129.37, 129.23, 127.15, 59.17, 38.12, 16.06. **HRMS** (ESI): calcd for $C_{14}H_{13}N_4NaSCl$ $[M + Na]^+$ 327.0447, found 327.0451.

1-(2-(butylthio)-1-phenylethyl)-1H-benzo[d][1,2,3]triazole

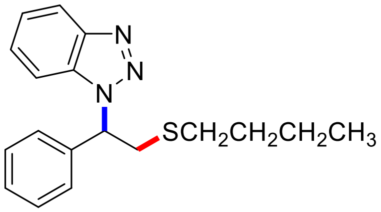

6a

Following the general procedure, using (petroleum ether : EtOAc = 9 : 1) as the eluant afforded a yellow liquid (54.7 mg, 88% yield). **^{1}H NMR** (400 MHz, $CDCl_3$): δ 8.08 (d, *J* = 8.2 Hz, 1H), 7.45-7.40 (m, 4H), 7.37-7.30 (m, 4H), 5.93 (dd, *J* = 8.6, 6.3 Hz, 1H), 3.93 (dd, *J* = 13.8, 8.7 Hz, 1H), 3.59 (dd, *J* = 13.8, 6.3 Hz, 1H), 2.41 (td, *J* = 7.3, 4.3 Hz, 2H), 1,54-1.46 (m, 2H), 1.36-1.27 (m, 2H), 0.86 (t, *J* = 7.3 Hz, 3H). **^{13}C NMR** (100 MHz, $CDCl_3$): δ 146.17, 138.25, 133.19, 129.02, 128.76, 127.40, 127.10, 124.07, 120.06, 109.77, 64.23, 37.21, 32.70, 31.68, 21.83, 13.65. **HRMS** (ESI): calcd for $C_{18}H_{21}N_3NaS$ $[M + Na]^+$ 334.1354, found 334.1359.

1-(2-((2-phenoxyethyl)thio)-1-phenylethyl)-1H-benzo[d][1,2,3]triazole

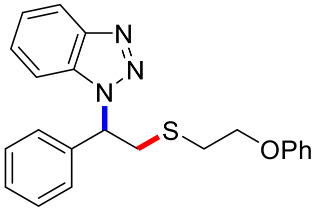

6b

Following the general procedure, using (petroleum ether : EtOAc = 9 : 1) as the eluant afforded a yellow liquid (66.8 mg, 89% yield). **^{1}H NMR** (400 MHz, $CDCl_3$): δ 8.11 (d, *J* = 8.1 Hz, 1H), 7.45-7.29 (m, 11H), 7.00 (t, *J* = 7.3

Hz, 1H), 6.89 (d, *J* = 7.7 Hz, 2H), 6.01 (dd, *J* = 8.9, 6.1 Hz, 1H), 4.14-4.07 (m, 3H), 3.76 (dd, *J* = 13.9, 6.1 Hz, 1H), 2.87 (t, *J* = 6.2 Hz, 2H). ^{13}**C NMR** (100 MHz, $CDCl_3$): δ 158.40, 146.21, 138.13, 133.25, 129.65, 129.10, 128.86, 127.48, 127.09, 124.15, 121.26, 120.13, 114.71, 109.74, 68.25, 64.10, 37.92, 32.18. **HRMS** (ESI): calcd for $C_{22}H_{21}N_3ONaS$ $[M + Na]^+$ 398.1303, found 398.1306.

1-(2-(decylthio)-1-phenylethyl)-1H-benzo[d][1,2,3]triazole

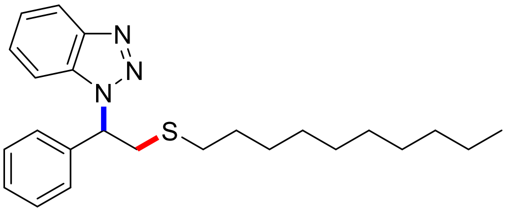

6c

Following the general procedure, using (petroleum ether : EtOAc = 9 : 1) as the eluant afforded a yellow solid (71.1 mg, 90% yield), Mp = 60-61℃. ^{1}**H NMR** (400 MHz, $CDCl_3$): δ 8.09 (d, *J* = 8.3 Hz, 1H), 7.46-7.32 (m, 8H), 5.92 (dd, *J* = 8.6, 6.4 Hz, 1H), 3.93 (dd, *J* = 13.8, 8.6 Hz, 1H), 3.59 (dd, *J* = 13.8, 6.4 Hz, 1H), 2.41 (td, *J* = 7.3, 5.2 Hz, 2H), 1.51 (q, *J* = 7.3 Hz, 2H), 1.27-1.25 (m, 14H), 0.91 (t, *J* = 6.8 Hz, 3H). ^{13}**C NMR** (100 MHz, $CDCl_3$): δ 146.18, 138.24, 133.18, 129.02, 128.76, 127.37, 127.10, 124.05, 120.11, 109.71, 64.28, 37.21, 33.06, 31.94, 29.63, 29.57, 29.52, 29.34, 29.18, 28.73, 22.72, 14.17. **HRMS** (ESI): calcd for $C_{24}H_{33}N_3NaS$ $[M + Na]^+$ 418.2293, found 418.2294.

1-(2-((cyclopropylmethyl)thio)-1-phenylethyl)-1H-benzo[d][1,2,3]triazole

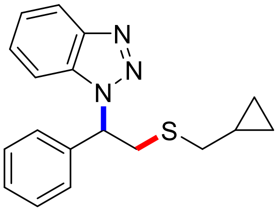

6d

Following the general procedure, using (petroleum ether : EtOAc = 9 : 1) as the eluant afforded a yellow liquid (47.6 mg, 77% yield). ^{1}**H NMR** (400 MHz, $CDCl_3$): δ 8.08 (d, *J* = 8.2 Hz, 1H), 7.46-7.32 (m, 8H), 5.96 (dd, *J* = 8.7, 6.3 Hz, 1H), 3.99 (dd, *J* = 13.9, 8.7 Hz, 1H), 3.66 (dd, *J* = 13.9, 6.3 Hz, 1H), 2.37 (dd, *J* = 7.0, 2.4 Hz, 2H), 0.93 (tt, *J* = 7.5, 4.8 Hz, 1H), 0.55 (dd, *J* = 8.1, 1.4 Hz, 2H), 0.18-0.15 (m, 2H). ^{13}**C NMR** (100 MHz, $CDCl_3$): δ 146.17, 138.23, 133.21, 129.03, 128.77, 127.39, 127.09, 124.06, 120.10, 109.73, 64.27, 38.38, 37.04, 11.19, 5.50, 5.48. **HRMS** (ESI): calcd for $C_{18}H_{19}N_3NaS$ $[M + Na]^+$ 332.1197, found 332.1201.

1-(1-phenyl-2-((4-(p-tolyloxy)butyl)thio)ethyl)-1H-benzo[d][1,2,3]triazole

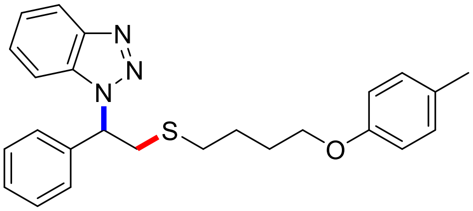

6e

Following the general procedure, using (petroleum ether : EtOAc = 9 : 1) as the eluant afforded a yellow liquid (76.7 mg, 92% yield). **^{1}H NMR** (400 MHz, $CDCl_3$): δ 8.13-8.09 (m, 1H), 7.48-7.33 (m, 9H), 7.11-7.09 (m, 2H), 6.83-6.79 (m, 2H), 5.96-5.90 (m, 1H), 4.02-3.89 (m, 3H), 3.67-3.58 (m, 1H), 2.55-2.47 (m, 2H), 2.32 (s, 3H), 1.82-1.71 (m, 4H). **^{13}C NMR** (100 MHz, $CDCl_3$): δ 156.84, 146.17, 138.17, 133.17, 129.95, 129.91, 129.06, 128.83, 127.45, 127.10, 124.12, 120.14, 114.40, 109.71, 67.28, 64.26, 37.13, 32.70, 28.28, 26.21, 20.53. **HRMS** (ESI): calcd for $C_{25}H_{27}N_3ONaS$ $[M + Na]^+$ 440.1773, found 440.1775.

1-(2-((4-(4-chlorophenoxy)butyl)thio)-1-phenylethyl)-1H-benzo[d][1,2,3]triazole

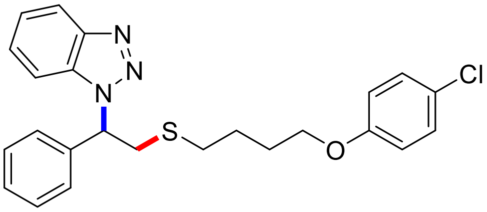

6f

Following the general procedure, using (petroleum ether : EtOAc = 9 : 1) as the eluant afforded a yellow solid (79.6 mg, 91% yield), Mp = 80-81℃. **^{1}H NMR** (400 MHz, $CDCl_3$): δ 8.09 (d, *J* = 8.3 Hz, 1H), 7.45-7.42 (m, 4H), 7.39-7.33 (m, 4H), 7.24 (d, *J* = 8.9 Hz, 2H), 6.81 (d, *J* = 9.0 Hz, 2H), 5.92 (dd, *J* = 8.7, 6.2 Hz, 1H), 3.96 (dd, *J* = 13.9, 8.7 Hz, 1H), 3.89 (t, *J* = 6.0 Hz, 2H), 3.60 (dd, *J* = 13.9, 6.3 Hz, 1H), 2.48 (td, *J* = 6.9, 4.9 Hz, 2H), 1.81-1.69 (m, 4H). **^{13}C NMR** (100 MHz, $CDCl_3$): δ 157.59, 146.17, 138.16, 133.18, 129.36, 129.08, 128.85, 127.48, 127.08, 125.49, 124.16, 120.10, 115.82, 109.73, 67.55, 64.28, 37.12, 32.64, 28.11, 26.09. **HRMS** (ESI): calcd for $C_{24}H_{24}N_3ONaSCl$ $[M + Na]^+$ 460.1226, found 460.1231.

1-(2-((4-(4-fluorophenoxy)butyl)thio)-1-phenylethyl)-1H-benzo[d][1,2,3]triazole

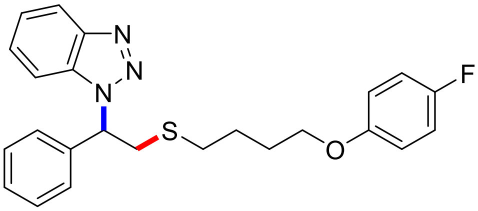

6g

Following the general procedure, using (petroleum ether : EtOAc = 9 : 1) as the eluant afforded a yellow solid (74.1 mg, 88% yield), Mp = 77-78℃. **^{1}H NMR** (400 MHz, $CDCl_3$): δ 8.10 (d, *J* = 8.2 Hz, 1H), 7.46-7.43 (m, 4H), 7.40-

7.34 (m, 4H), 6.99 (t, J = 8.7 Hz, 2H), 6.82 (dd, J = 9.1, 4.3 Hz, 2H), 5.92 (dd, J = 8.7, 6.3 Hz, 1H), 3.96 (dd, J = 13.9, 8.7 Hz, 1H), 3.89 (t, J = 6.0 Hz, 2H), 3.61 (dd, J = 13.8, 6.3 Hz, 1H), 2.49 (td, J = 6.9, 5.0 Hz, 2H), 1.82-1.70 (m, 4H). **^{13}C NMR** (100 MHz, $CDCl_3$): δ 158.42, 156.06, 155.08, 146.16, 138.15, 133.17, 129.07, 128.84, 127.46, 127.07, 124.15, 120.13, 115.93, 115.70, 115.49, 115.41, 109.69, 67.85, 64.29, 37.11, 32.67, 28.21, 26.12. **^{19}F NMR** (375 MHz, $CDCl_3$) δ -124.17 (1F); **HRMS** (ESI): calcd for $C_{24}H_{24}N_3ONaSF$ $[M + Na]^+$ 444.1522, found 444.1529.

5-isopropyl-2-methylphenyl (R)-4-(1-(1H-benzo[d][1,2,3]triazol-1-yl)-2-(methylthio)ethyl)benzoate

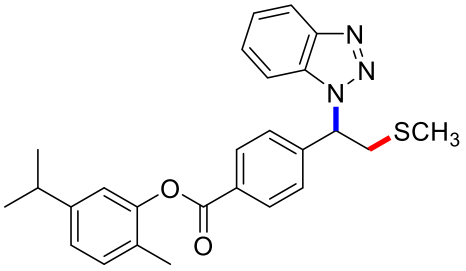

7a

Following the general procedure, using (petroleum ether : EtOAc = 9 : 1) as the eluant afforded a yellow liquid (77.5 mg, 87% yield). **^{1}H NMR** (400 MHz, $CDCl_3$): δ 8.24 (d, J = 8.5 Hz, 2H), 8.14 (d, J = 8.3 Hz, 1H), 7.61 (d, J = 8.4 Hz, 2H), 7.52-7.45 (m, 2H), 7.43-7.39 (m, 1H), 7.22 (d, J = 7.8 Hz, 1H), 7.09 (dd, J = 7.8, 1.8 Hz, 1H), 6.99 (d, J = 1.8 Hz, 1H), 6.03 (dd, J = 8.4, 6.5 Hz, 1H), 3.97 (dd, J = 13.9, 8.4 Hz, 1H), 3.64 (dd, J = 13.9, 6.5 Hz, 1H), 2.92 (p, J = 6.9 Hz, 1H), 2.18 (s, 3H), 2.08 (s, 3H), 1.27 (d, J = 6.9 Hz, 6H). **^{13}C NMR** (100 MHz, $CDCl_3$): δ 164.25, 149.35, 148.27, 146.20, 143.54, 133.12, 131.05, 130.93, 130.10, 127.78, 127.42, 127.31, 124.41, 124.38, 120.32, 119.81, 109.42, 63.35, 39.20, 33.64, 23.98, 16.55, 15.90. **HRMS** (ESI): calcd for $C_{26}H_{27}N_3O_2NaS$ $[M + Na]^+$ 468.1722, found 468.1720.

5-chloro-2-(2,4-dichlorophenoxy)phenyl (R)-4-(1-(1H-benzo[d][1,2,3]triazol-1-yl)-2-(methylthio)ethyl)benzoate

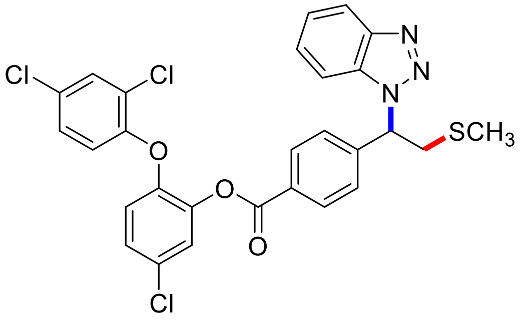

7b

Following the general procedure, using (petroleum ether : EtOAc = 9 : 1) as the eluant afforded a yellow liquid (92.1 mg, 79% yield). **^{1}H NMR** (400 MHz, $CDCl_3$): δ 8.12 (d, J = 8.2 Hz, 1H), 8.05 (d, J = 8.4 Hz, 2H), 7.54 (d, J = 8.4 Hz, 2H), 7.50-7.38 (m, 3H), 7.32 (d, J = 2.5 Hz, 1H), 7.27 (d, J = 2.5 Hz, 1H), 7.23 (dd, J = 8.8, 2.5 Hz, 1H), 7.14 (dd, J = 8.8, 2.5 Hz, 1H), 6.91 (dd, J = 8.8, 2.6 Hz, 2H), 5.99 (dd, J = 8.4, 6.5 Hz, 1H), 3.94 (dd, J = 13.9, 8.4

Hz, 1H), 3.60 (dd, *J* = 13.9, 6.5 Hz, 1H), 2.06 (s, 3H). ^{13}**C NMR** (100 MHz, $CDCl_3$): δ 163.38, 150.98, 146.84, 146.18, 143.97, 141.63, 133.08, 131.00, 130.37, 129.58, 129.31, 128.87, 128.17, 127.80, 127.36, 127.26, 126.03, 124.59, 124.39, 120.60, 120.33, 120.31, 109.37, 63.28, 39.17, 16.54. **HRMS** (ESI): calcd for $C_{28}H_{20}N_3O_3NaSCl_3$ $[M + Na]^+$ 606.0189, found 606.0188.

^{1}H, ^{13}C and ^{19}F NMR spectra of products

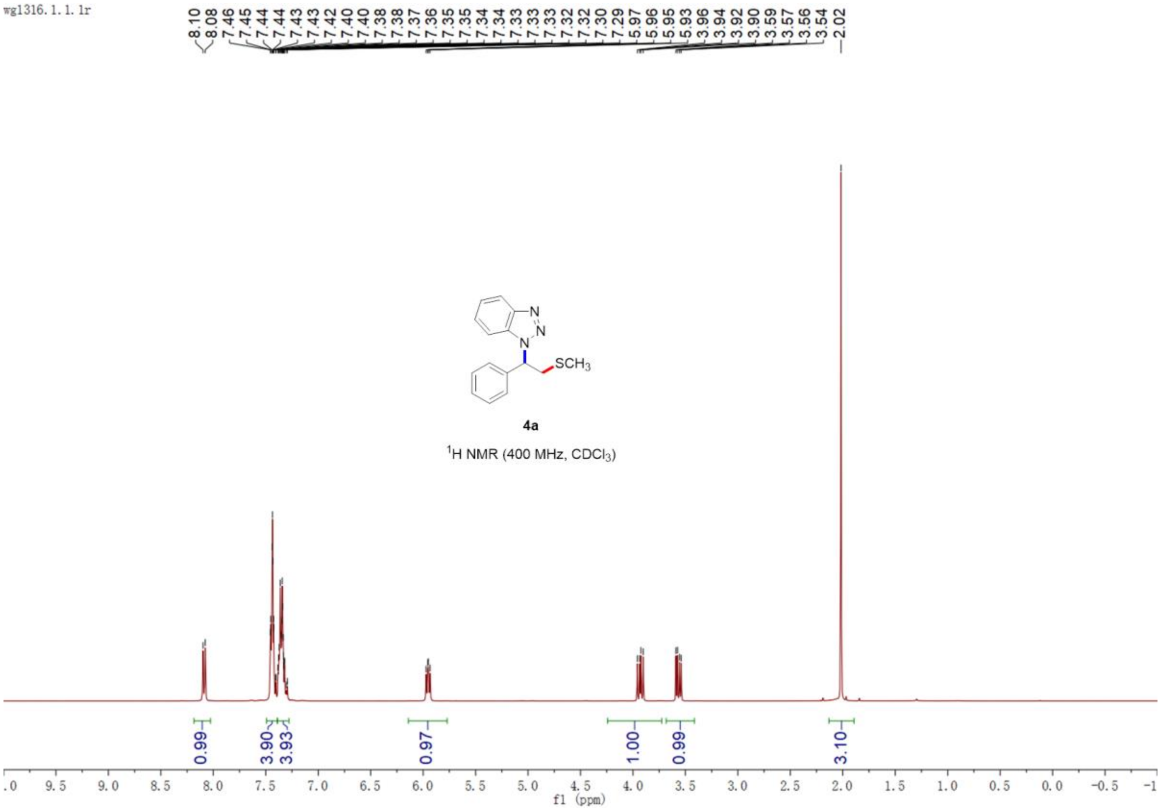

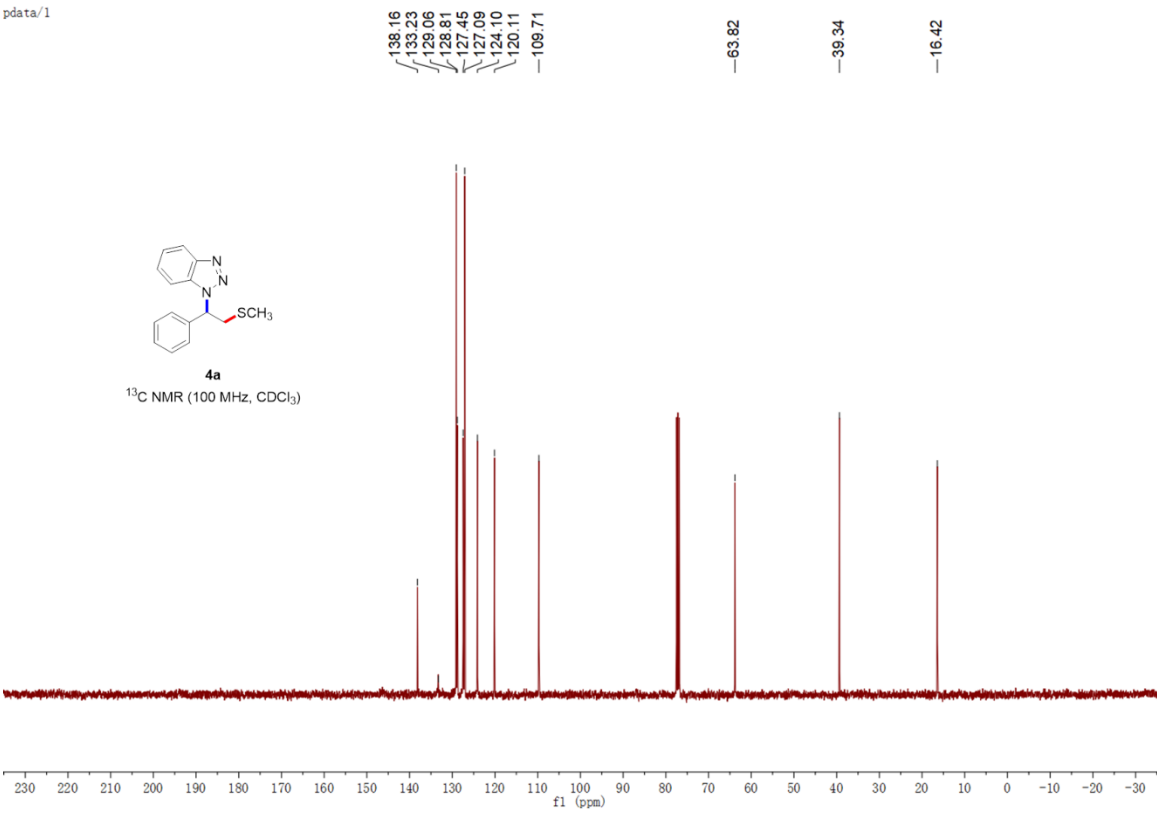

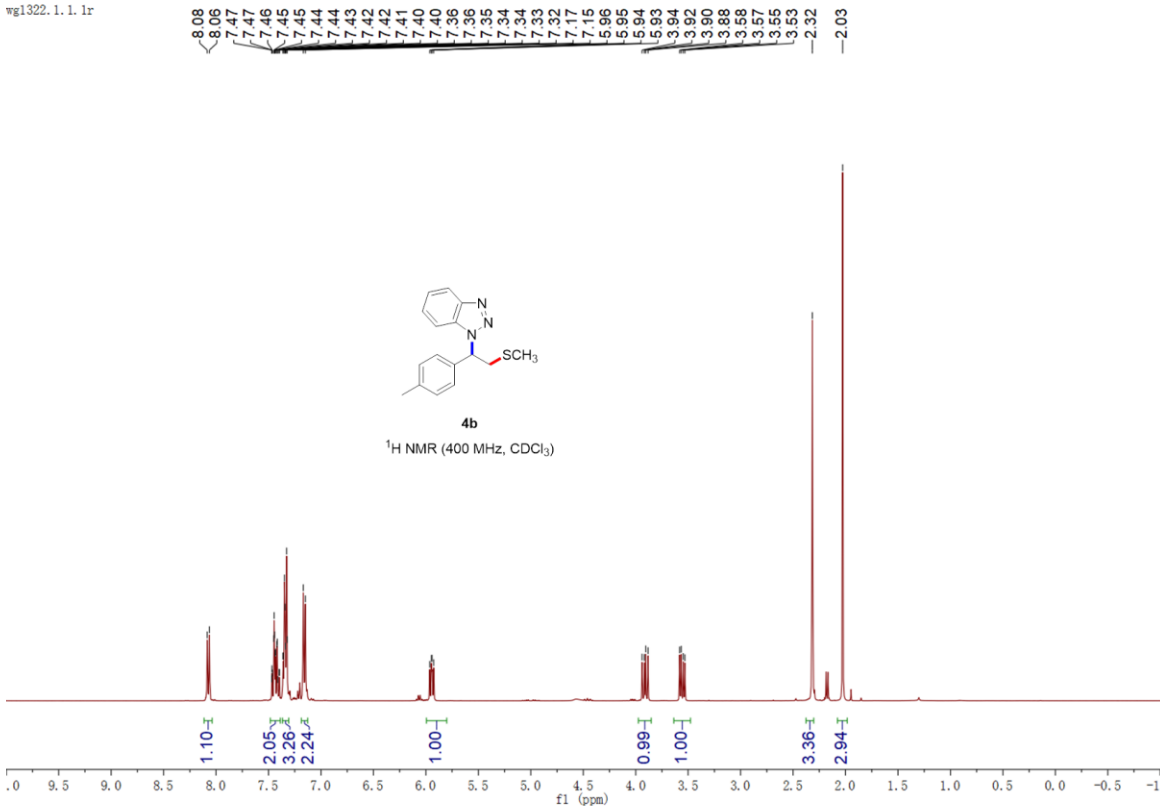
wg1322.1.1.1r
8.08
8.06
7.47
7.47
7.46
7.45
7.45
7.44
7.44
7.43
7.42
7.42
7.41
7.40
7.40
7.36
7.36
7.35
7.34
7.34
7.34
7.33
7.32
7.17
7.15
5.96
5.95
5.94
5.93
3.94
3.92
3.90
3.88
3.58
3.57
3.55
3.53
2.32
2.03
N
N
N
SCH3
4b
1H NMR (400 MHz, CDCl3)
1.10
2.05
3.26
2.24
1.00
0.99
1.00
3.36
2.94
f1 (ppm)

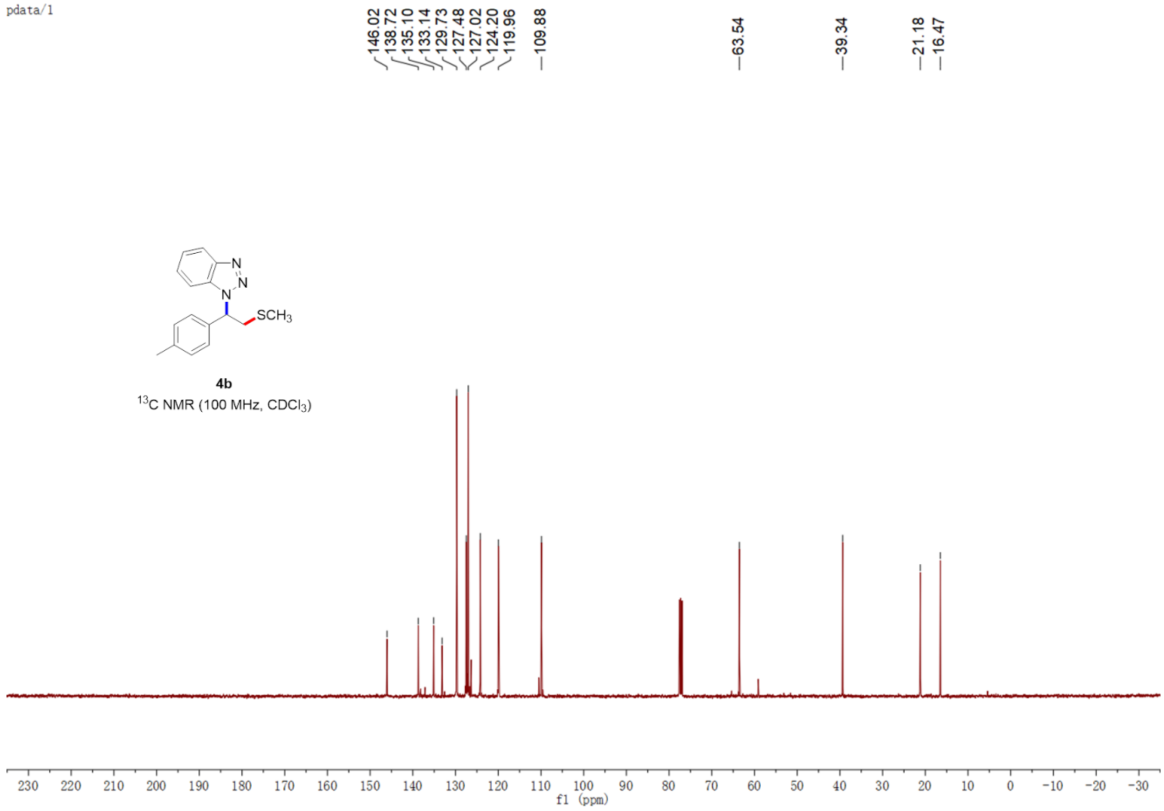
pdata/1
146.02
138.72
135.10
133.14
129.73
127.48
127.02
124.20
119.96
109.88
63.54
39.34
21.18
16.47
N
N
N
SCH3
4b
13C NMR (100 MHz, CDCl3)
f1 (ppm)

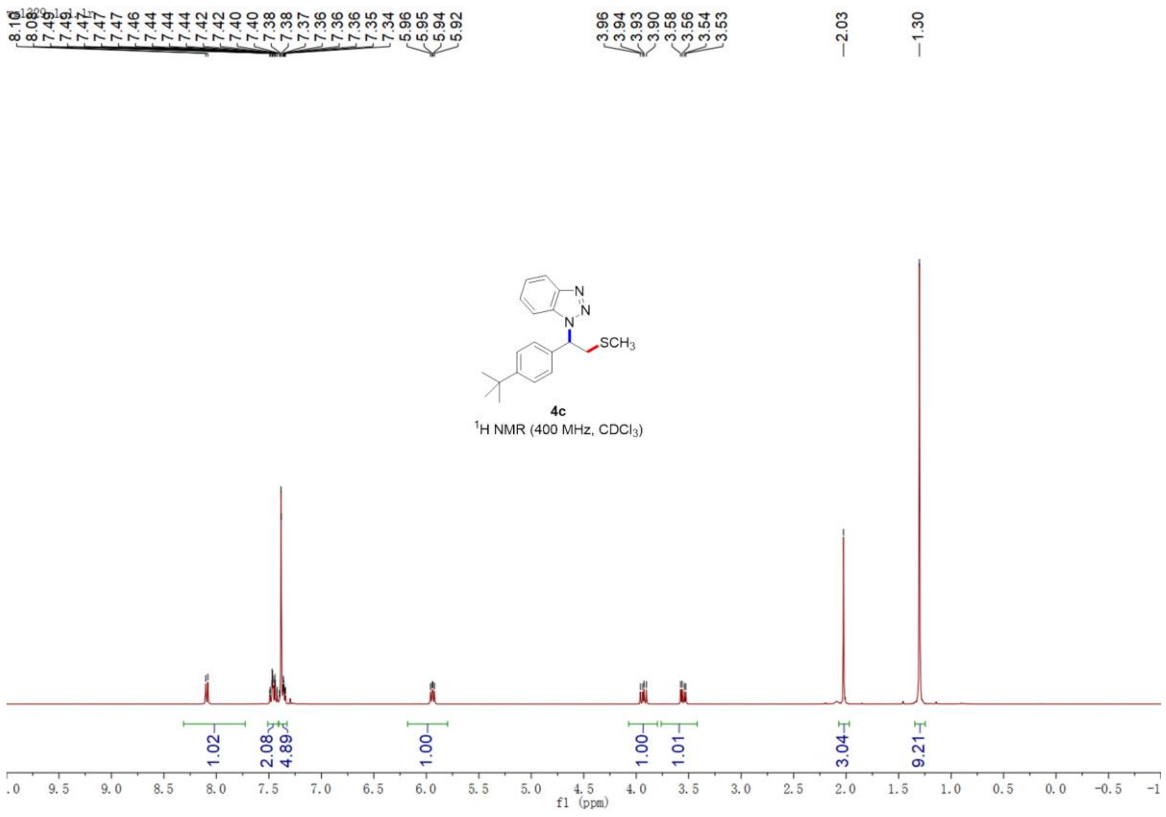

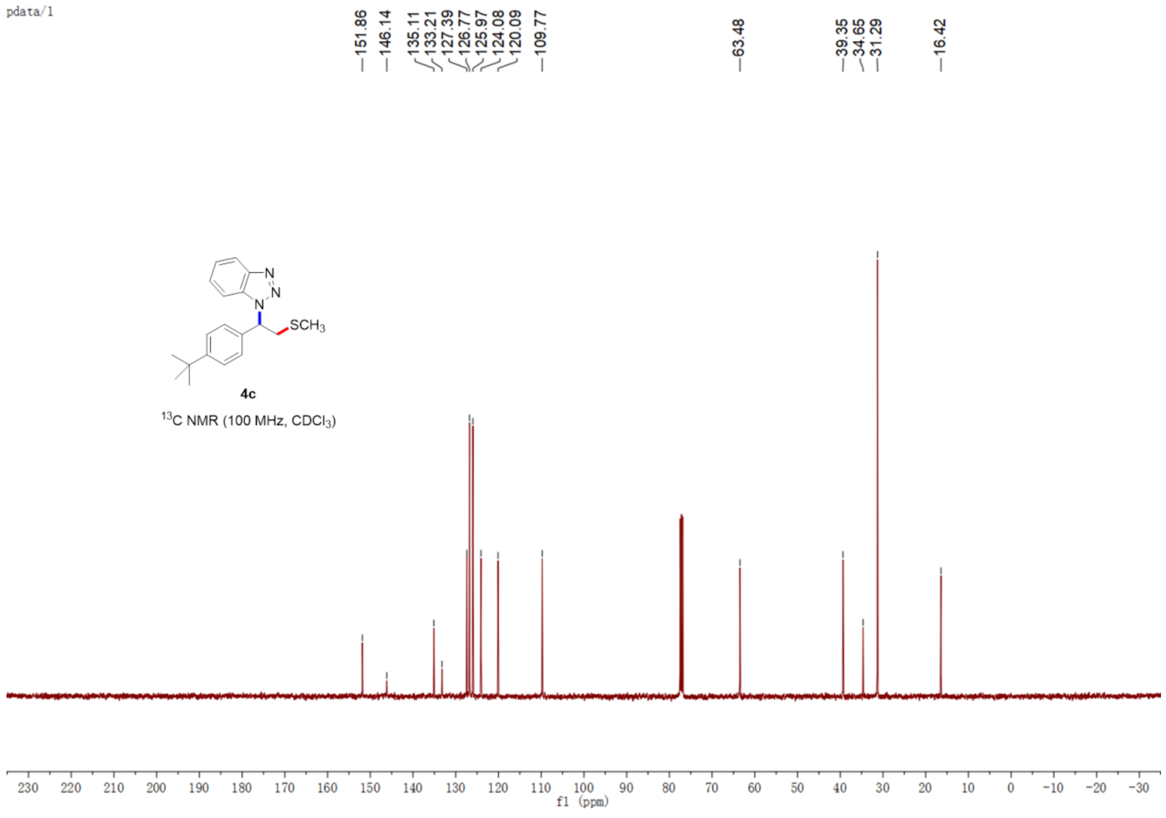

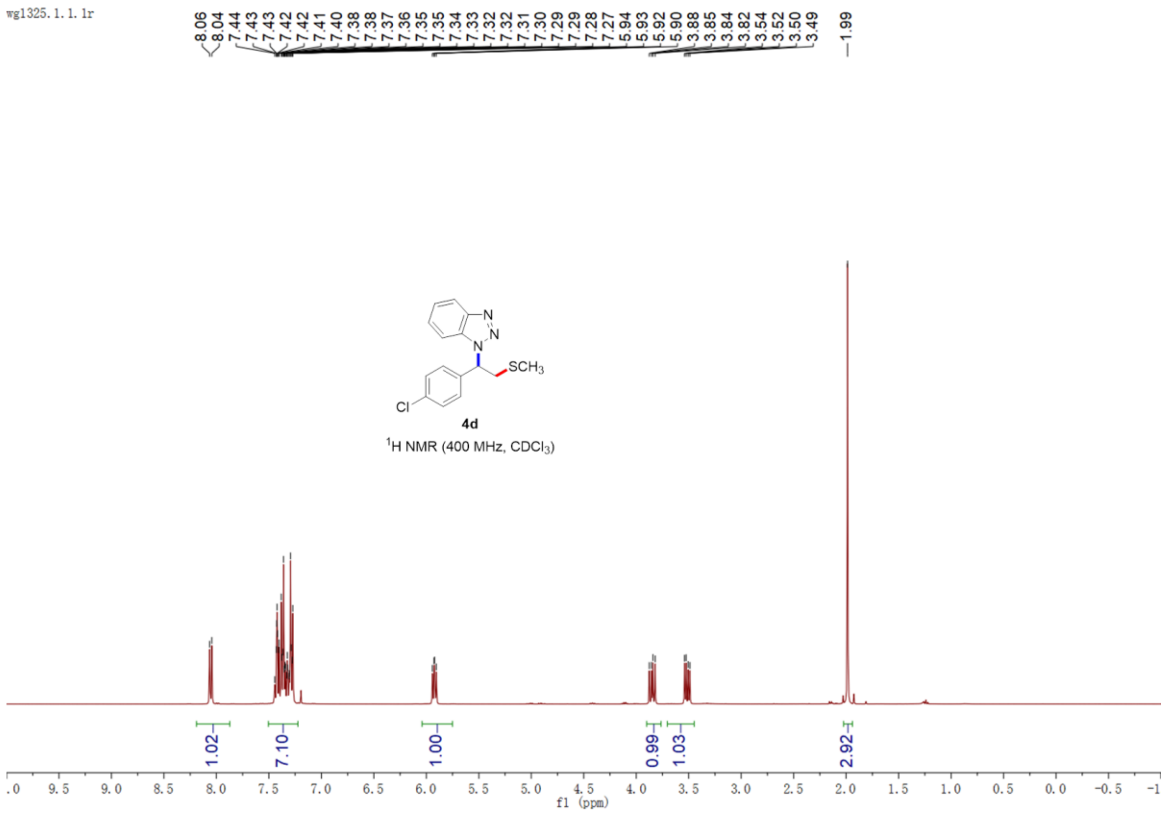
wg1325.1.1.1r
8.06
8.04
7.44
7.43
7.43
7.42
7.42
7.41
7.40
7.38
7.38
7.37
7.36
7.35
7.35
7.34
7.33
7.32
7.32
7.31
7.30
7.29
7.29
7.28
7.27
5.94
5.93
5.92
5.90
3.88
3.85
3.84
3.82
3.54
3.52
3.50
3.49
1.99
N
N
N
SCH3
Cl
4d
1H NMR (400 MHz, CDCl3)
1.02
7.10
1.00
0.99
1.03
2.92
f1 (ppm)

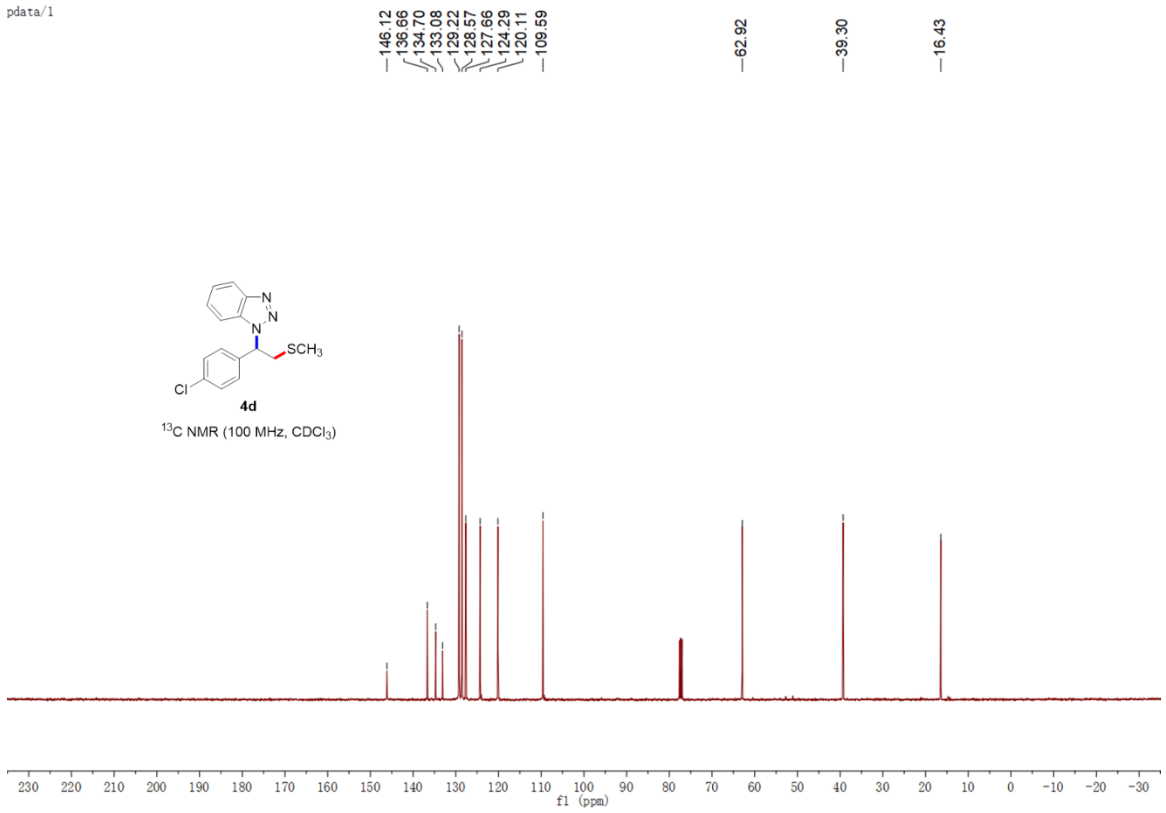
pdata/1
146.12
136.66
134.70
133.08
129.22
128.57
127.66
124.29
120.11
109.59
62.92
39.30
16.43
N
N
N
SCH3
Cl
4d
13C NMR (100 MHz, CDCl3)
f1 (ppm)

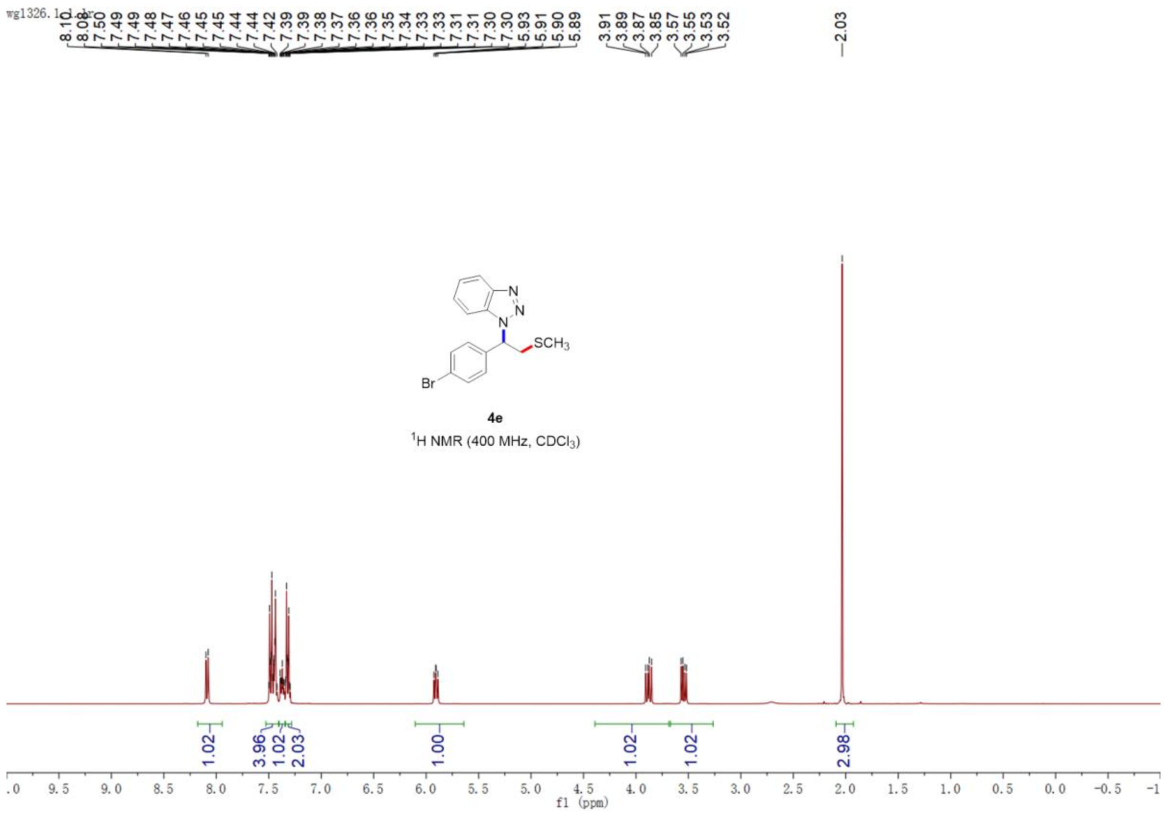

wg1326.1
8.10
8.08
7.50
7.49
7.48
7.47
7.46
7.45
7.45
7.44
7.44
7.42
7.39
7.39
7.38
7.37
7.36
7.36
7.35
7.34
7.33
7.33
7.31
7.31
7.30
7.30
5.93
5.91
5.90
5.89
3.91
3.89
3.87
3.85
3.57
3.55
3.53
3.52
2.03
SCH3
Br
N
N
N
4e
1H NMR (400 MHz, CDCl3)
1.02
3.96
1.02
2.03
1.00
1.02
1.02
2.98
f1 (ppm)

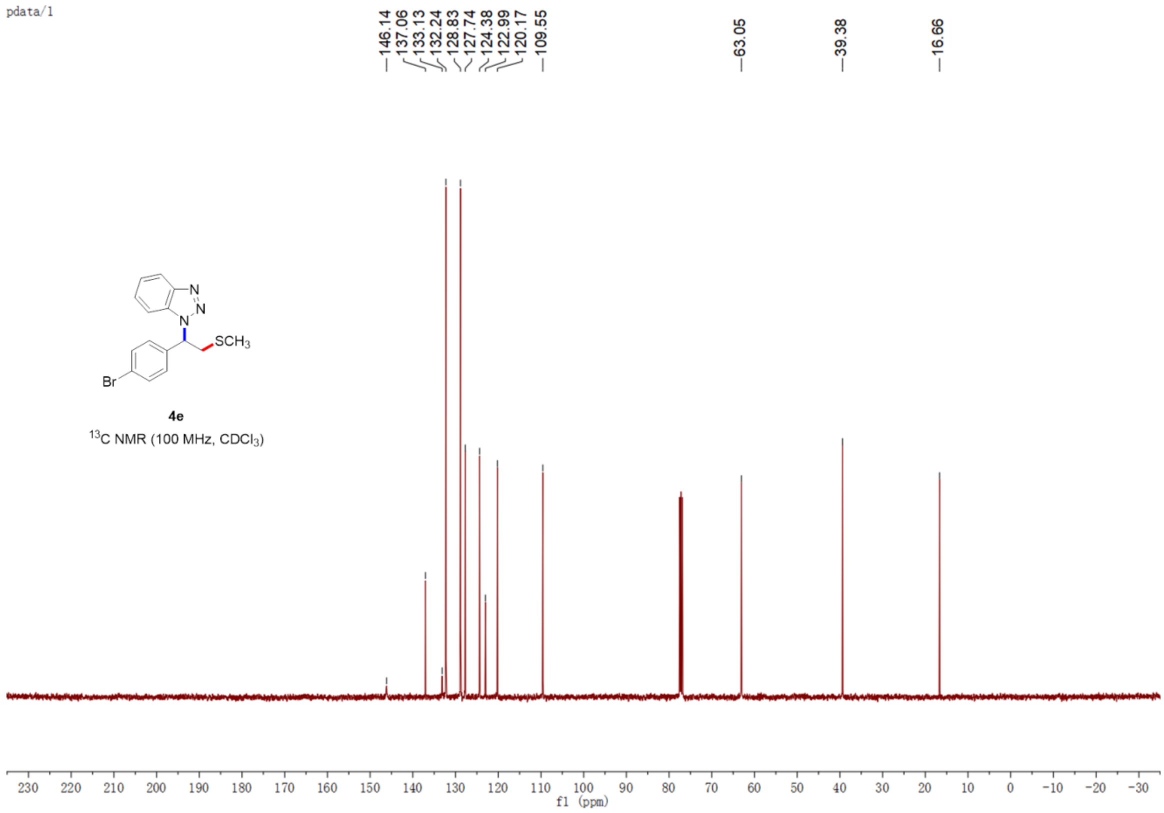

pdata/1
146.14
137.06
133.13
132.24
128.83
127.74
124.38
122.99
120.17
109.55
63.05
39.38
16.66
SCH3
Br
N
N
N
4e
13C NMR (100 MHz, CDCl3)
f1 (ppm)

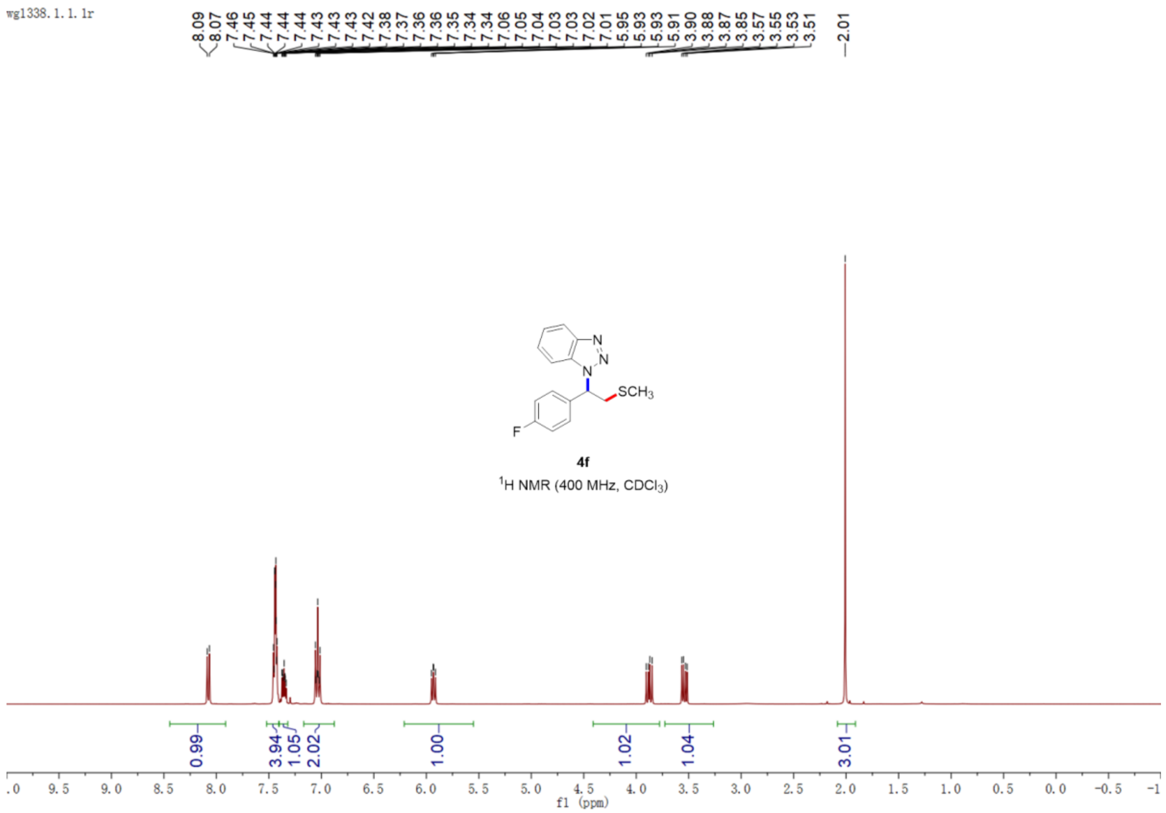
wg1338.1.1.1r
8.09
8.07
7.46
7.45
7.44
7.44
7.44
7.43
7.43
7.43
7.42
7.38
7.37
7.36
7.36
7.35
7.34
7.34
7.06
7.05
7.04
7.03
7.03
7.02
7.01
5.95
5.93
5.93
5.91
3.90
3.88
3.87
3.85
3.57
3.55
3.53
3.51
2.01
SCH3
F
4f
1H NMR (400 MHz, CDCl3)
0.99
3.94
1.05
2.02
1.00
1.02
1.04
3.01
f1 (ppm)

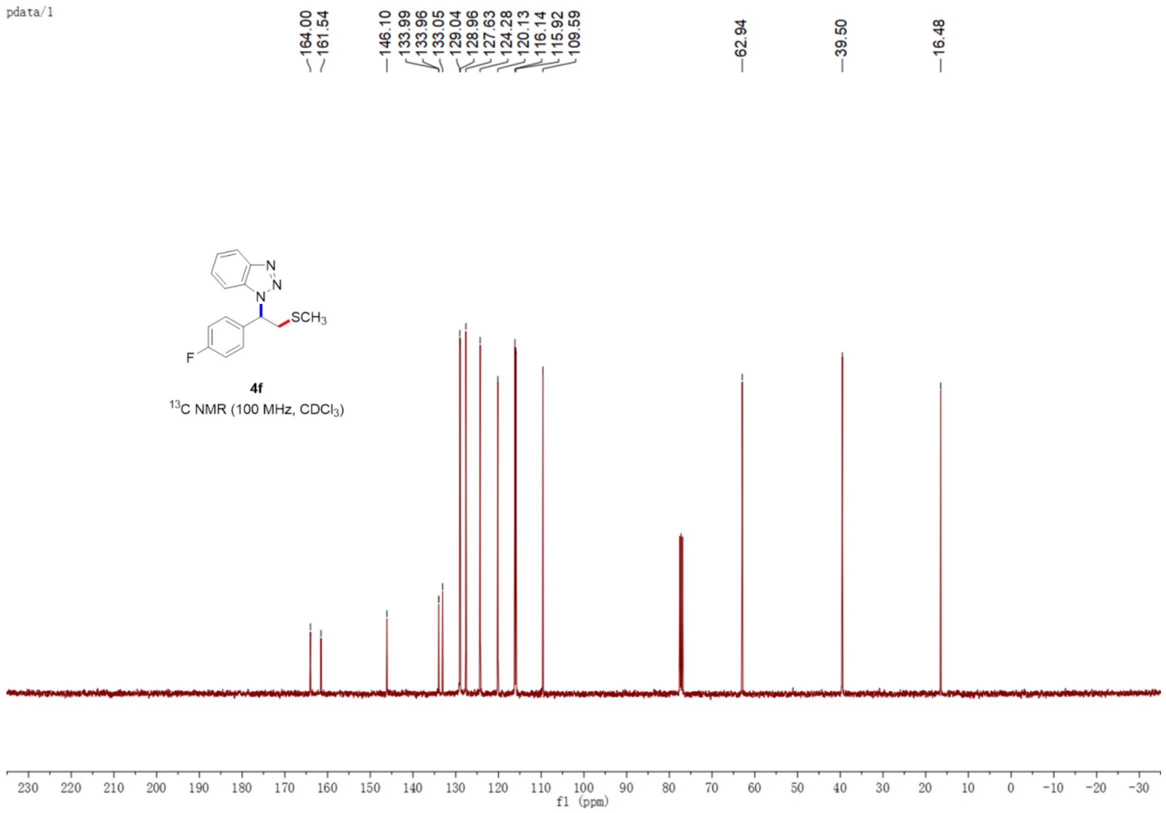
pdata/1
164.00
161.54
146.10
133.99
133.96
133.05
129.04
128.96
127.63
124.28
120.13
116.14
115.92
109.59
62.94
39.50
16.48
SCH3
F
4f
13C NMR (100 MHz, CDCl3)
f1 (ppm)

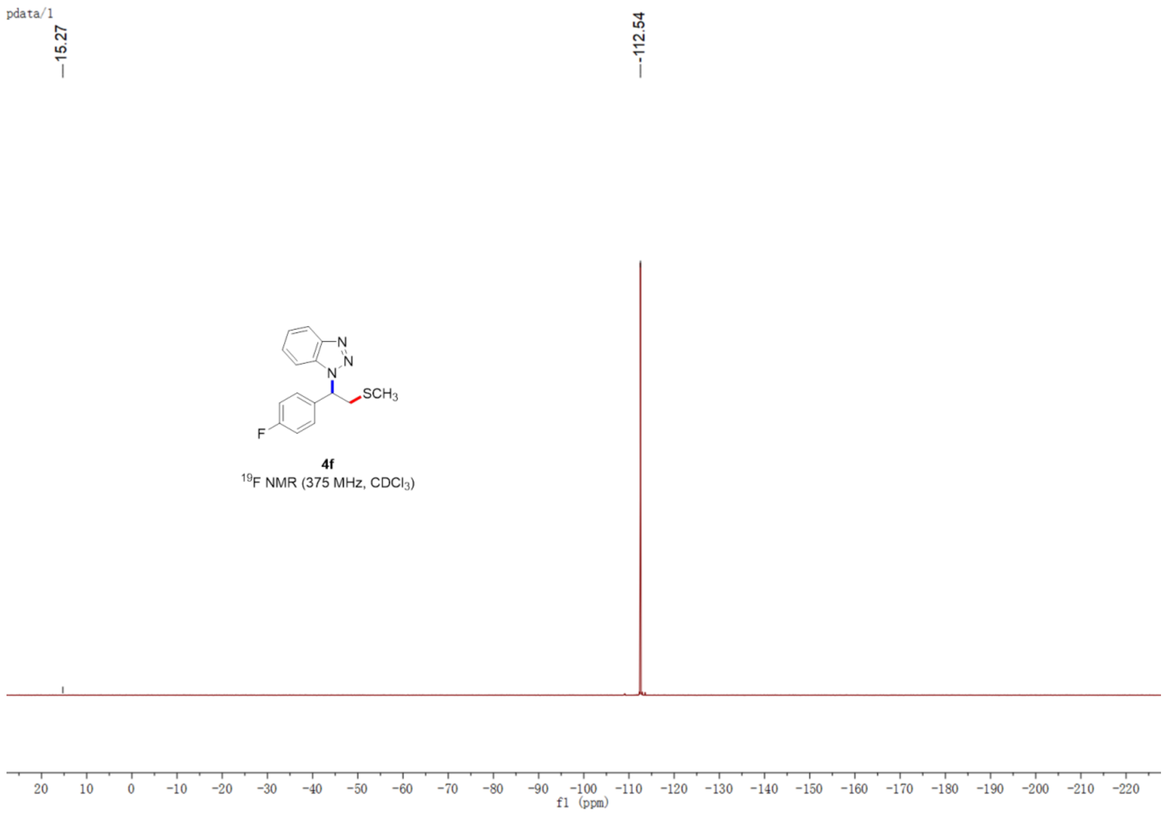
pdata/1
15.27
-112.54
SCH3
F
4f
19F NMR (375 MHz, CDCl3)
f1 (ppm)

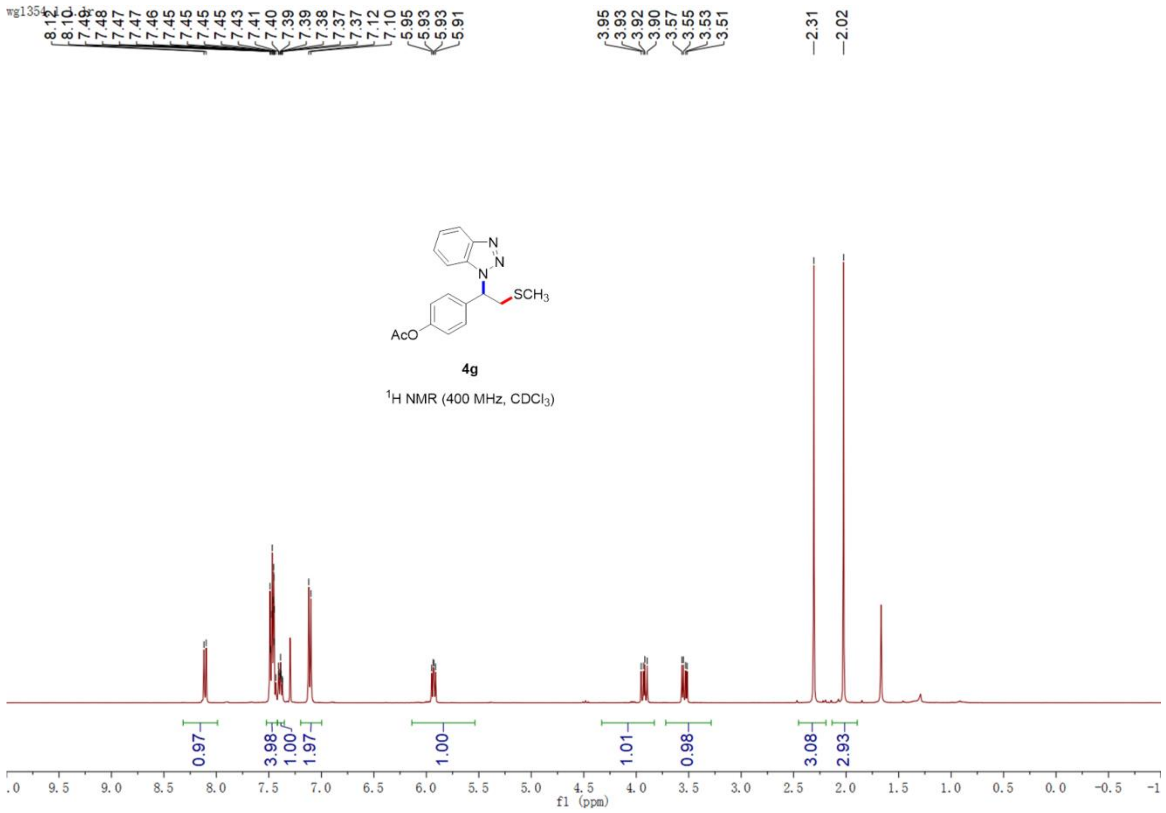
8.12
8.10
7.49
7.48
7.47
7.47
7.46
7.45
7.45
7.45
7.45
7.43
7.41
7.40
7.39
7.39
7.38
7.37
7.37
7.12
7.10
5.95
5.93
5.93
5.91
3.95
3.93
3.92
3.90
3.57
3.55
3.53
3.51
2.31
2.02
SCH3
AcO
4g
1H NMR (400 MHz, CDCl3)
0.97
3.98
1.00
1.97
1.00
1.01
0.98
3.08
2.93
f1 (ppm)

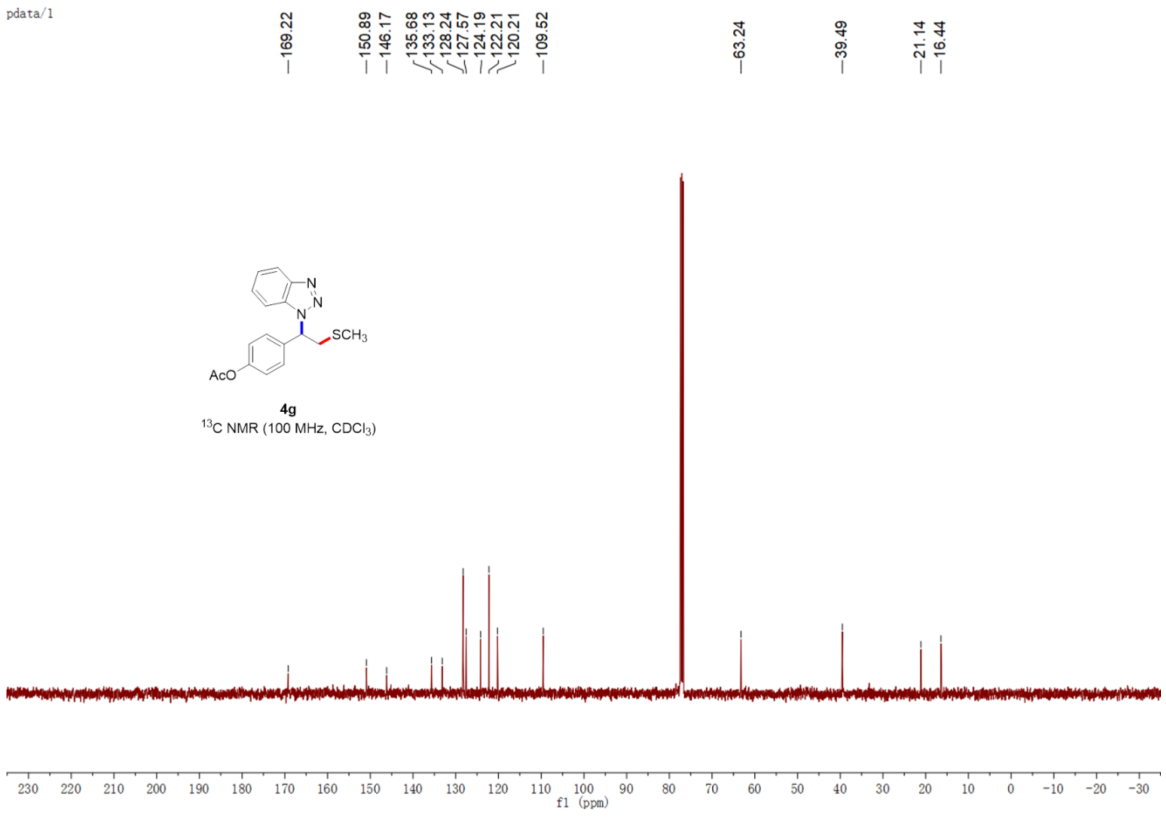
pdata/1
4g
13C NMR (100 MHz, CDCl3)
f1 (ppm)

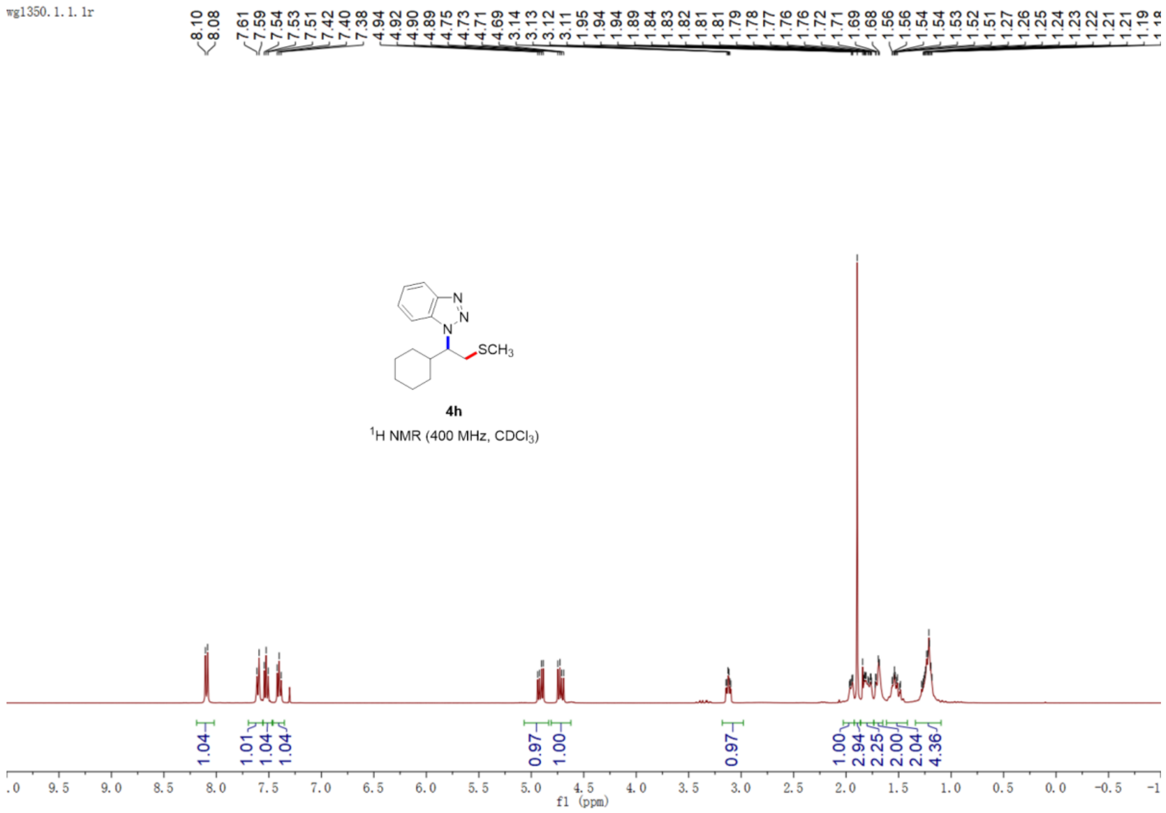
wg1350.1.1.1r
4h
1H NMR (400 MHz, CDCl3)
f1 (ppm)

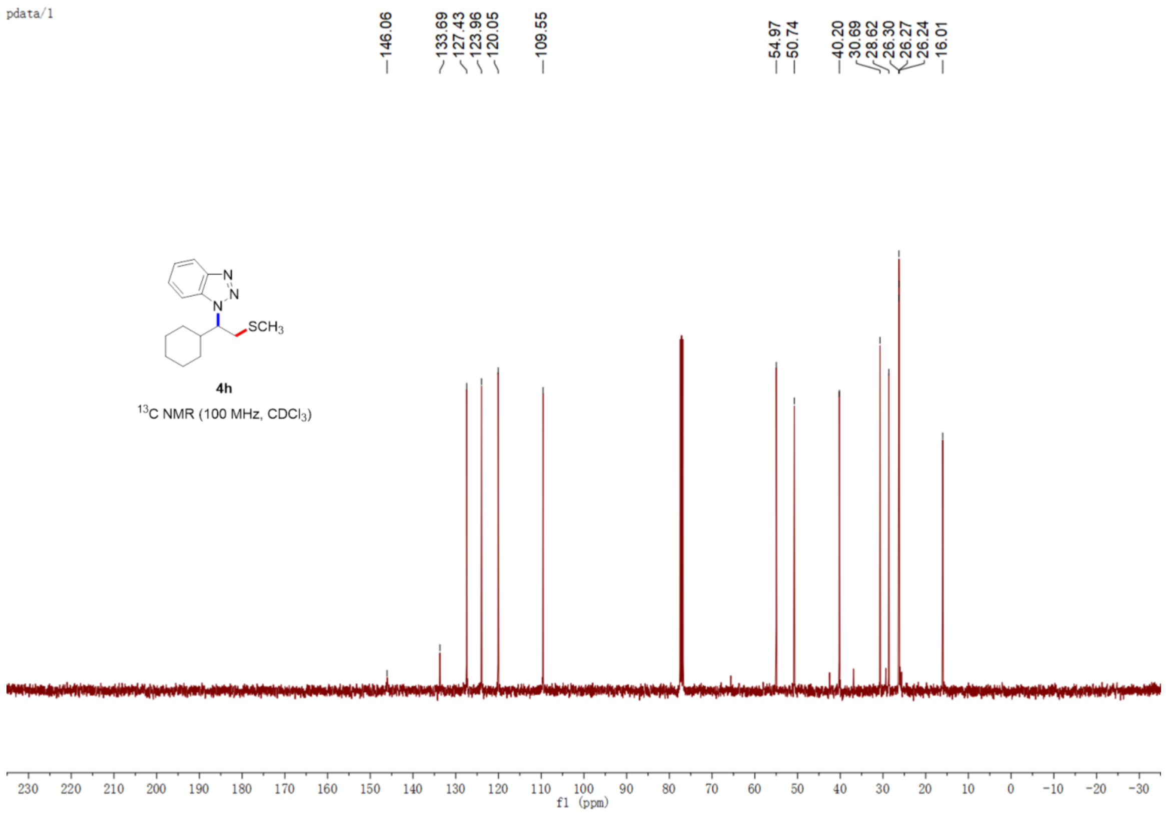
pdata/1
146.06
133.69
127.43
123.96
120.05
109.55
54.97
50.74
40.20
30.69
28.62
26.30
26.27
26.24
16.01
4h
13C NMR (100 MHz, CDCl3)
f1 (ppm)

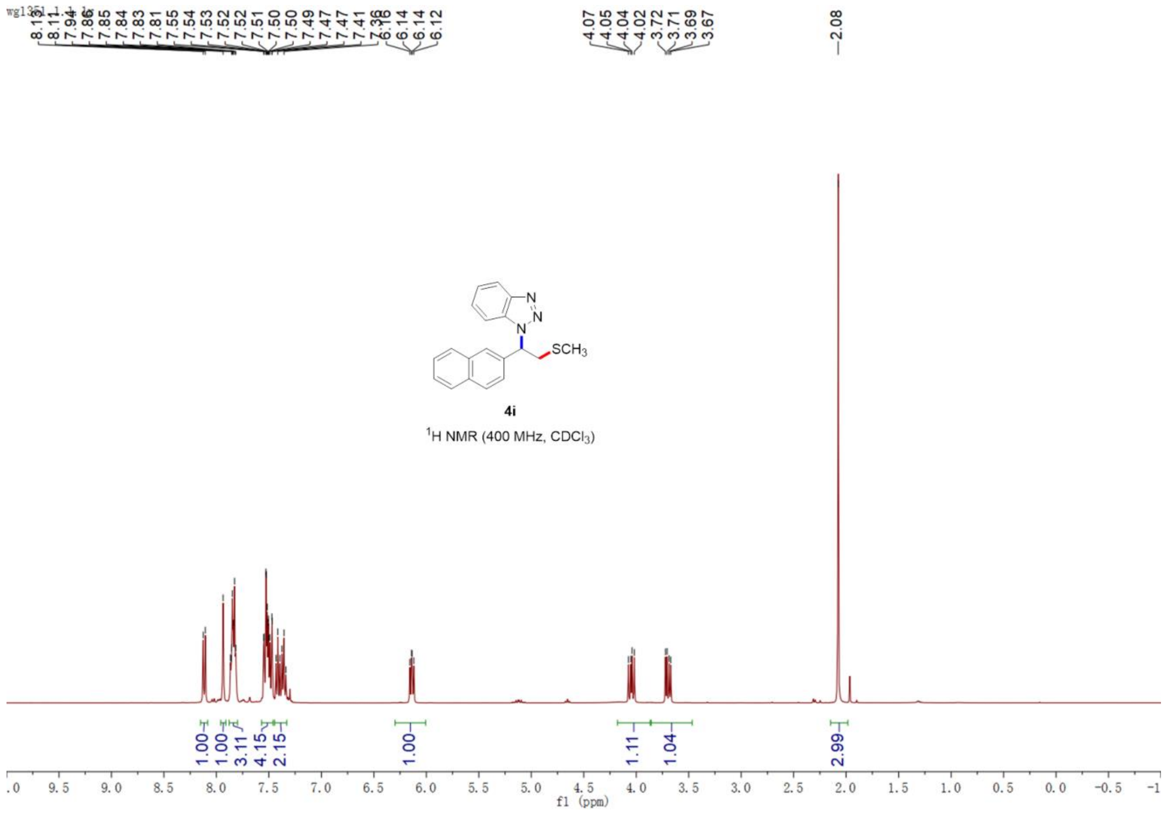
4.07
4.05
4.04
4.02
3.72
3.71
3.69
3.67
2.08
4i
1H NMR (400 MHz, CDCl3)
1.00
1.00
3.11
4.15
2.15
1.00
1.11
1.04
2.99
f1 (ppm)

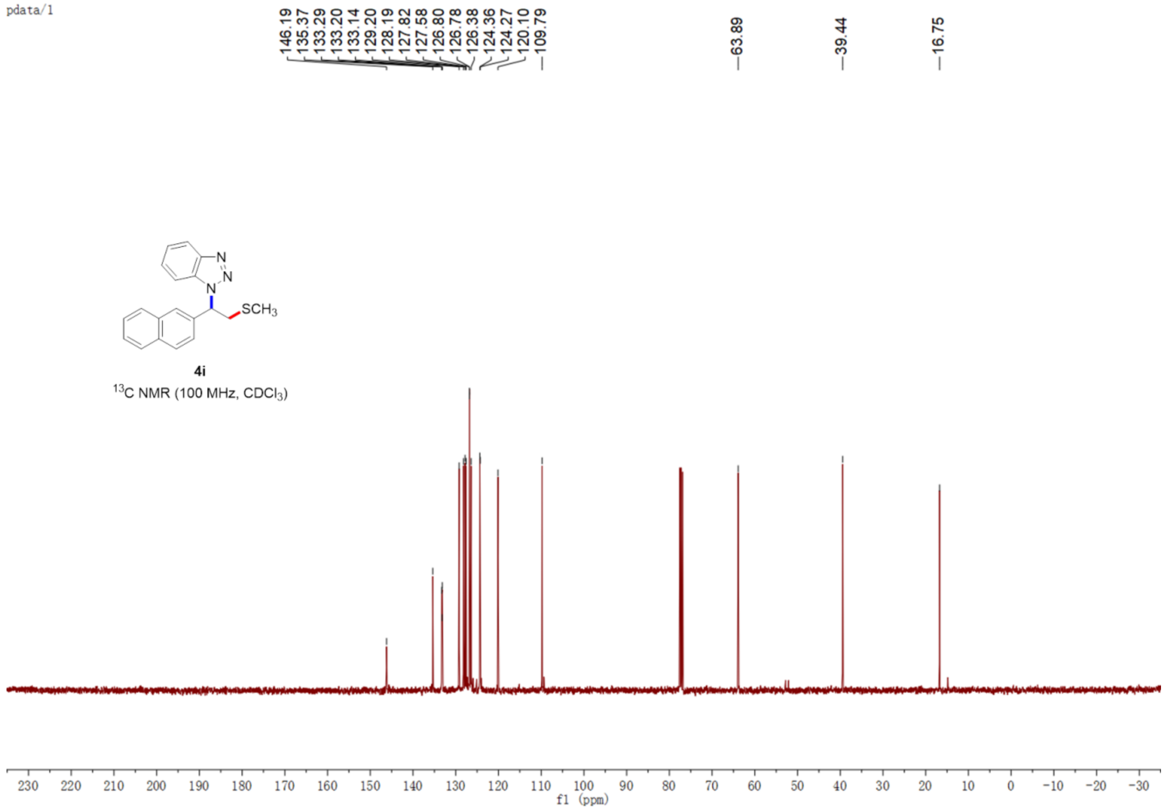
pdata/1
146.19
135.37
133.29
133.20
133.14
129.20
128.19
127.82
127.58
126.80
126.78
126.38
124.36
124.27
120.10
109.79
63.89
39.44
16.75
N
N
N
SCH3
4i
13C NMR (100 MHz, CDCl3)
230 220 210 200 190 180 170 160 150 140 130 120 110 100 90 80 70 60 50 40 30 20 10 0 -10 -20 -30
f1 (ppm)

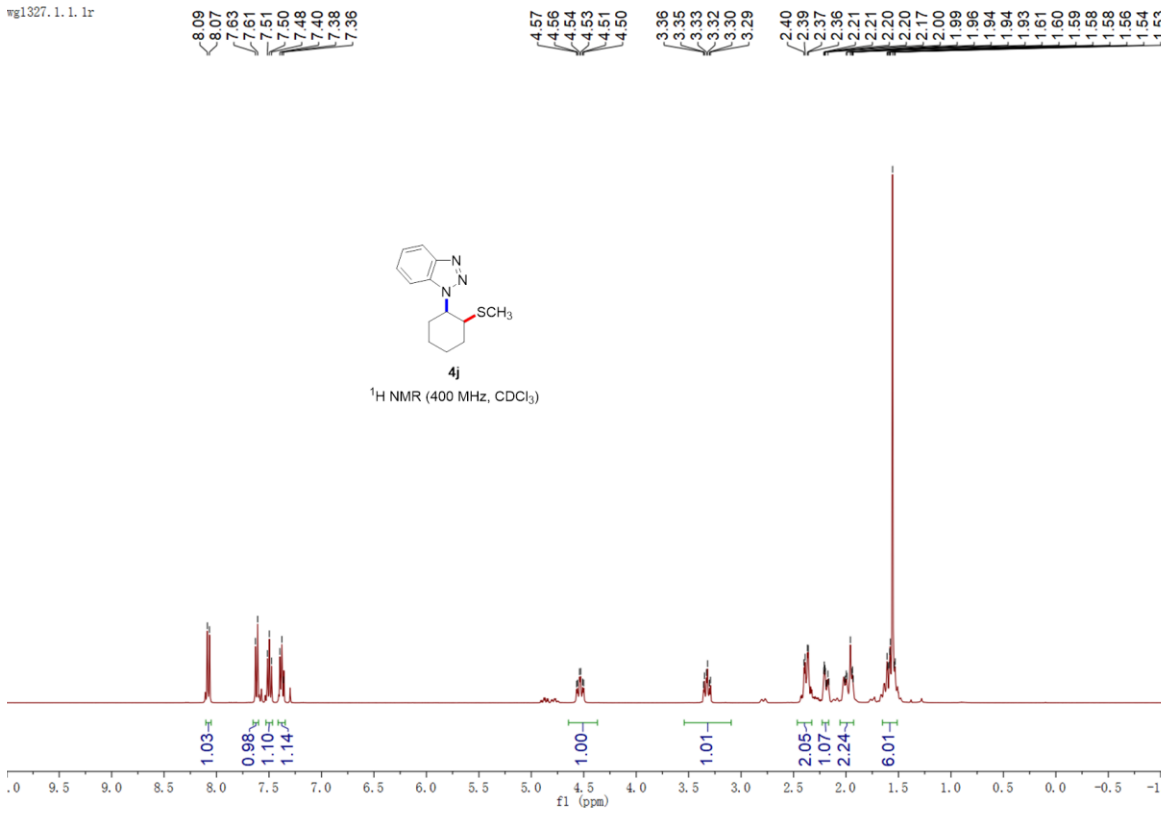
wg1327.1.1.1r
8.09
8.07
7.63
7.61
7.51
7.50
7.48
7.40
7.38
7.36
4.57
4.56
4.54
4.53
4.51
4.50
3.36
3.35
3.33
3.32
3.30
3.29
2.40
2.39
2.37
2.36
2.21
2.21
2.20
2.20
2.17
2.00
1.99
1.96
1.94
1.94
1.93
1.61
1.60
1.59
1.58
1.58
1.56
1.54
N
N
N
SCH3
4j
1H NMR (400 MHz, CDCl3)
1.03
0.98
1.10
1.14
1.00
1.01
2.05
1.07
2.24
6.01
9.5 9.0 8.5 8.0 7.5 7.0 6.5 6.0 5.5 5.0 4.5 4.0 3.5 3.0 2.5 2.0 1.5 1.0 0.5 0.0 -0.5
f1 (ppm)

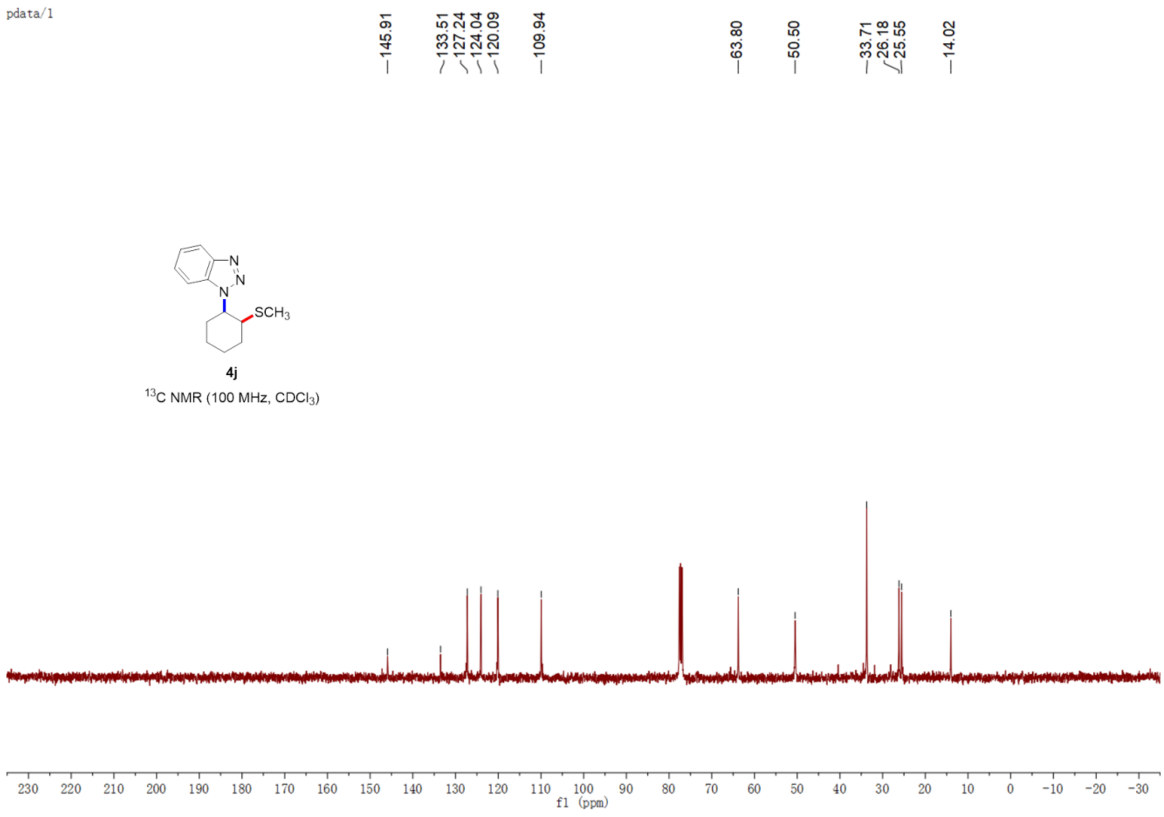
pdata/1
145.91
133.51
127.24
124.04
120.09
109.94
63.80
50.50
33.71
26.18
25.55
14.02
SCH3
4j
13C NMR (100 MHz, CDCl3)
230 220 210 200 190 180 170 160 150 140 130 120 110 100 90 80 70 60 50 40 30 20 10 0 -10 -20 -30
f1 (ppm)

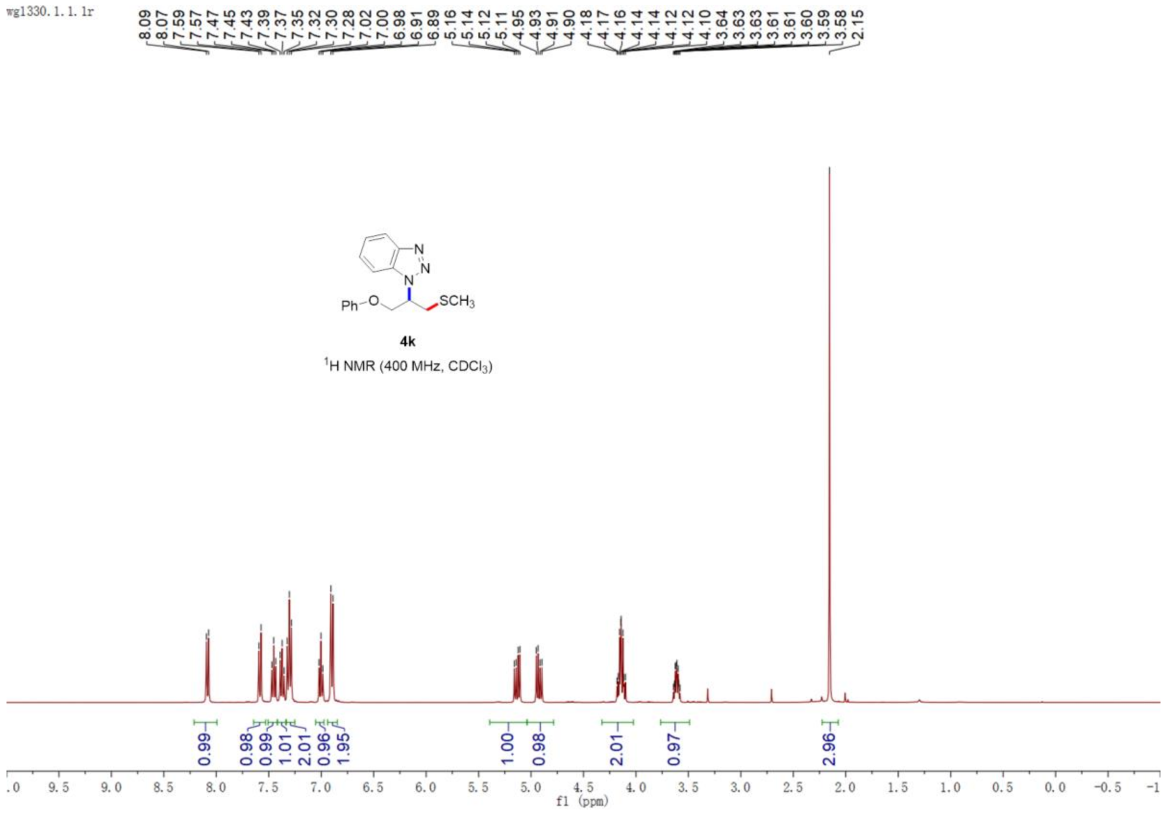
wg1330.1.1.1r
8.09
8.07
7.59
7.57
7.47
7.45
7.43
7.39
7.37
7.35
7.32
7.30
7.28
7.02
7.00
6.98
6.91
6.89
5.16
5.14
5.12
5.11
4.95
4.93
4.91
4.90
4.18
4.17
4.16
4.14
4.14
4.12
4.12
4.10
3.64
3.63
3.63
3.61
3.60
3.59
3.58
2.15
Ph
O
SCH3
4k
1H NMR (400 MHz, CDCl3)
0.99
0.98
0.99
1.01
2.01
0.96
1.95
1.00
0.98
2.01
0.97
2.96
.0 9.5 9.0 8.5 8.0 7.5 7.0 6.5 6.0 5.5 5.0 4.5 4.0 3.5 3.0 2.5 2.0 1.5 1.0 0.5 0.0 -0.5 -1
f1 (ppm)

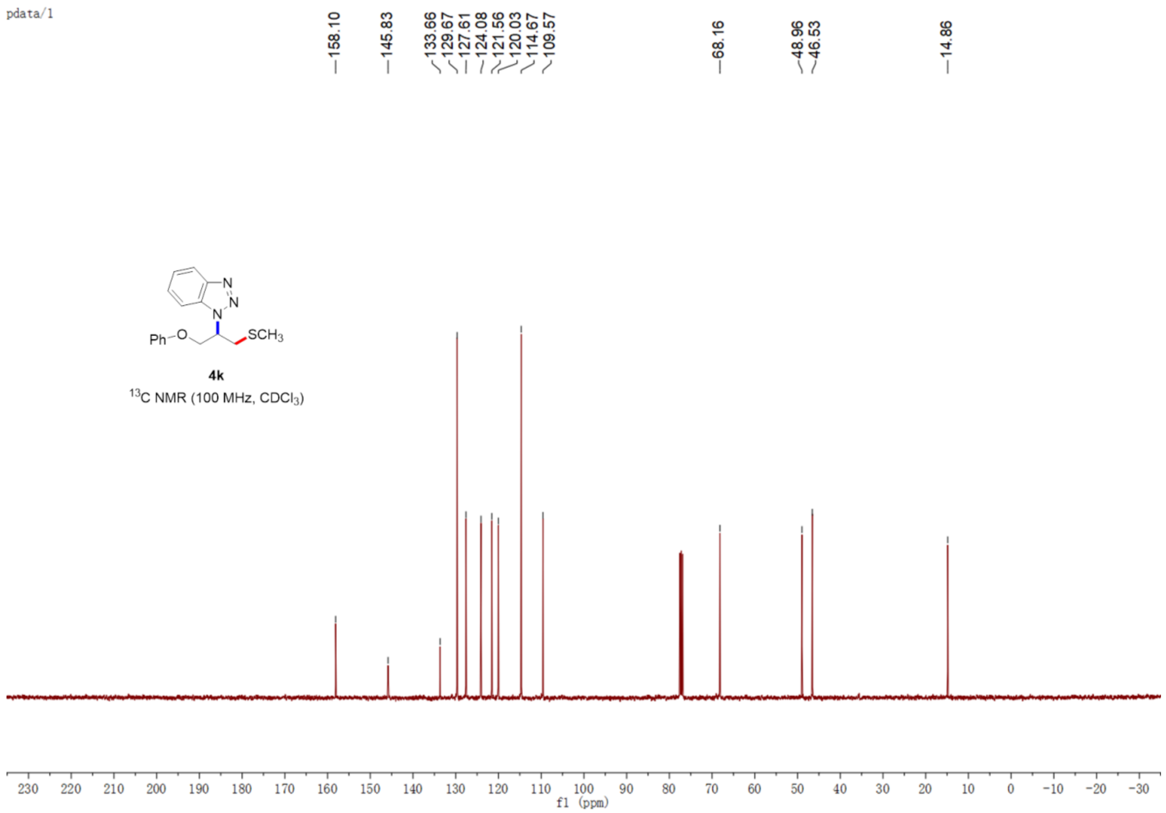
pdata/1
158.10
145.83
133.66
129.67
127.61
124.08
121.56
120.03
114.67
109.57
68.16
48.96
46.53
14.86
Ph–O
SCH3
4k
13C NMR (100 MHz, CDCl3)
f1 (ppm)

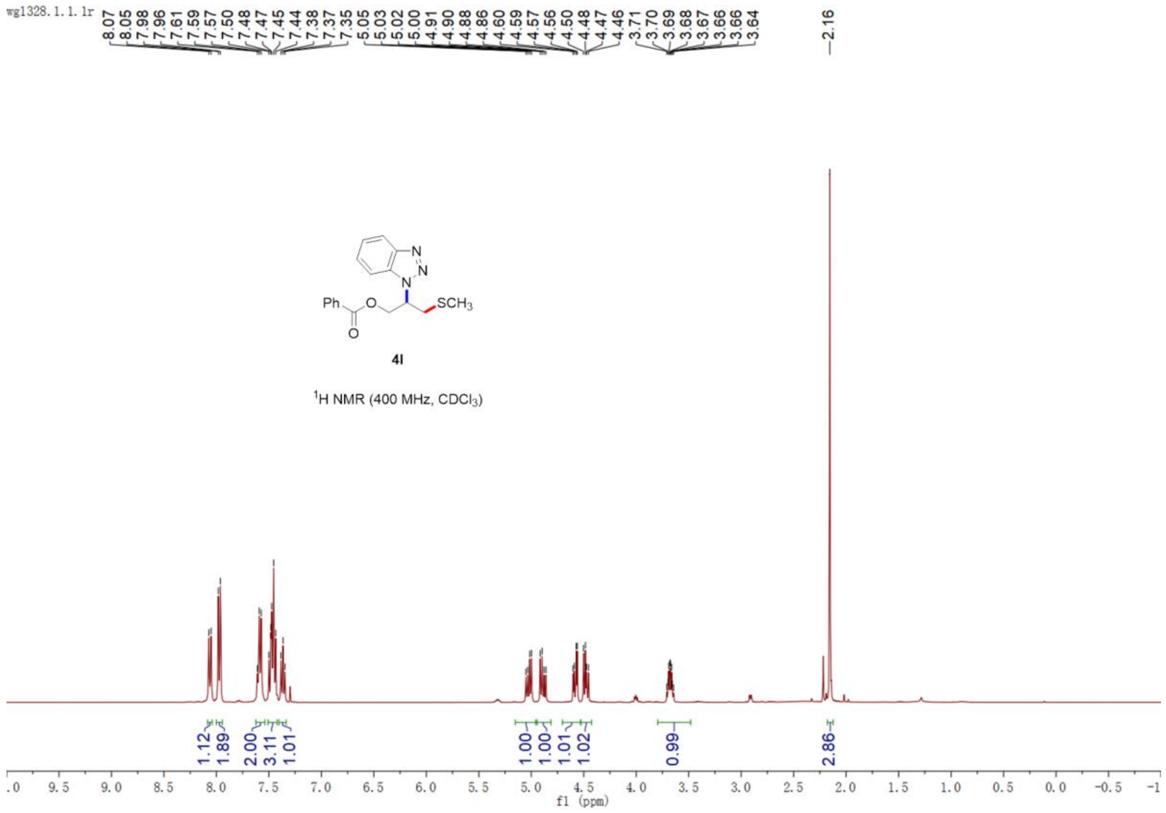
wg1328.1.1.1r
2.16
Ph
O
SCH3
4l
1H NMR (400 MHz, CDCl3)
1.12
1.89
2.00
3.11
1.01
1.00
1.00
1.01
1.02
0.99
2.86
f1 (ppm)

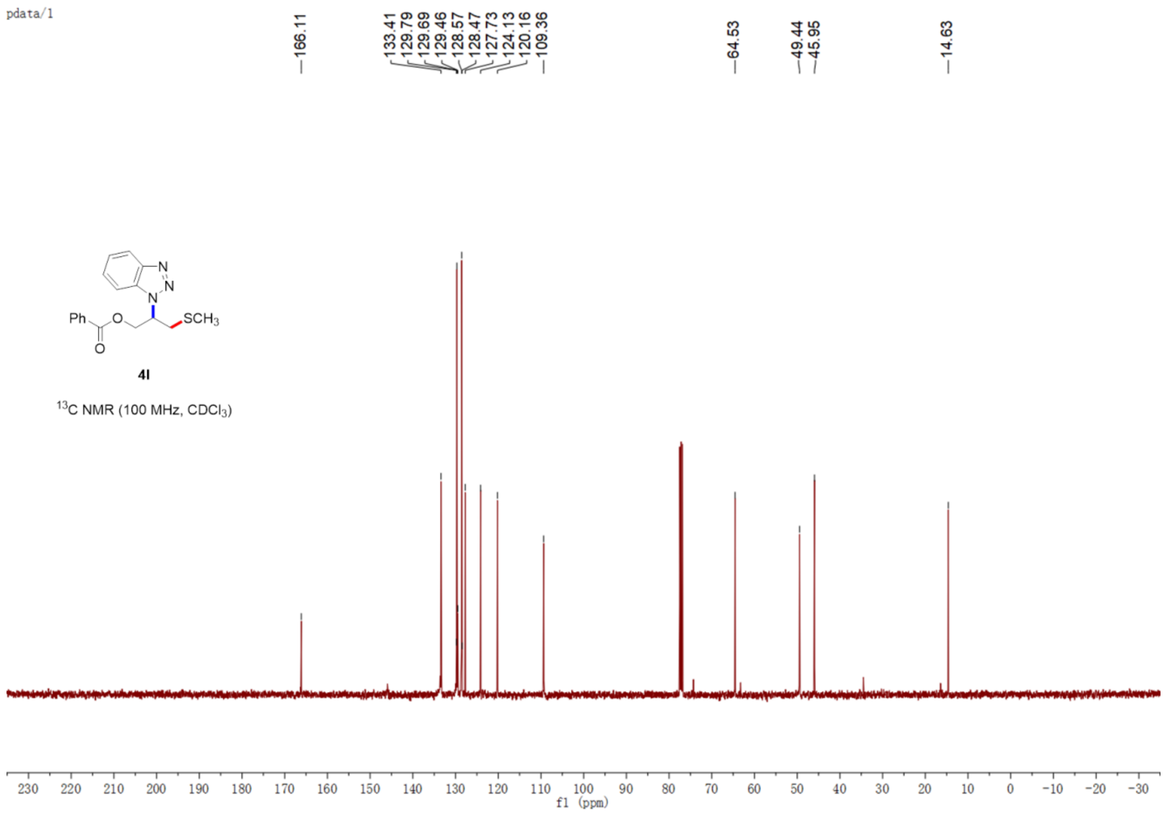
pdata/1
166.11
133.41
129.79
129.69
129.46
128.57
128.47
127.73
124.13
120.16
109.36
64.53
49.44
45.95
14.63
Ph
O
O
N
N
N
SCH3
4l
13C NMR (100 MHz, CDCl3)
f1 (ppm)

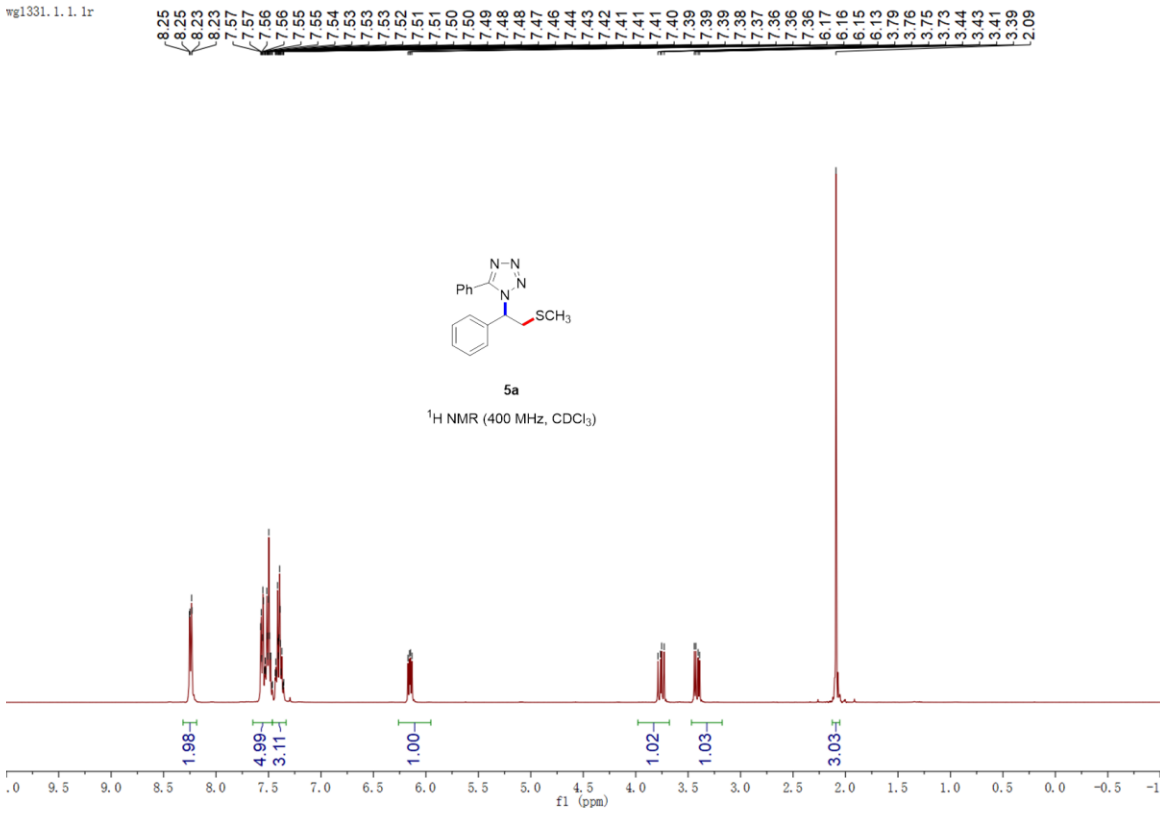
wg1331.1.1.1r
8.25
8.25
8.23
8.23
7.57
7.56
7.56
7.55
7.54
7.53
7.53
7.52
7.51
7.51
7.50
7.50
7.49
7.48
7.48
7.47
7.46
7.44
7.43
7.42
7.41
7.41
7.40
7.39
7.39
7.38
7.37
7.36
7.36
6.17
6.16
6.15
6.13
3.79
3.76
3.75
3.73
3.44
3.43
3.41
3.39
2.09
Ph
N
N
N
N
SCH3
5a
1H NMR (400 MHz, CDCl3)
1.98
4.99
3.11
1.00
1.02
1.03
3.03
f1 (ppm)

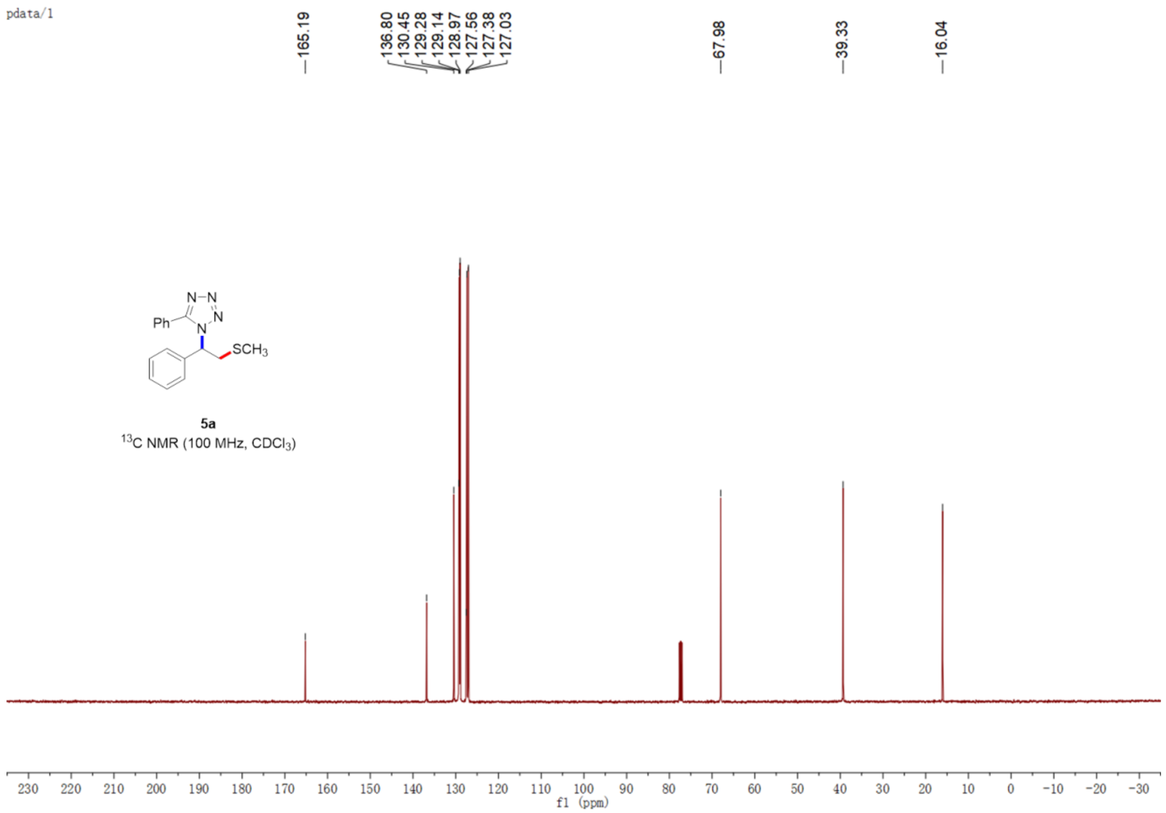
pdata/1
165.19
136.80
130.45
129.28
129.14
128.97
127.56
127.38
127.03
67.98
39.33
16.04
Ph
N
SCH3
5a
13C NMR (100 MHz, CDCl3)
f1 (ppm)

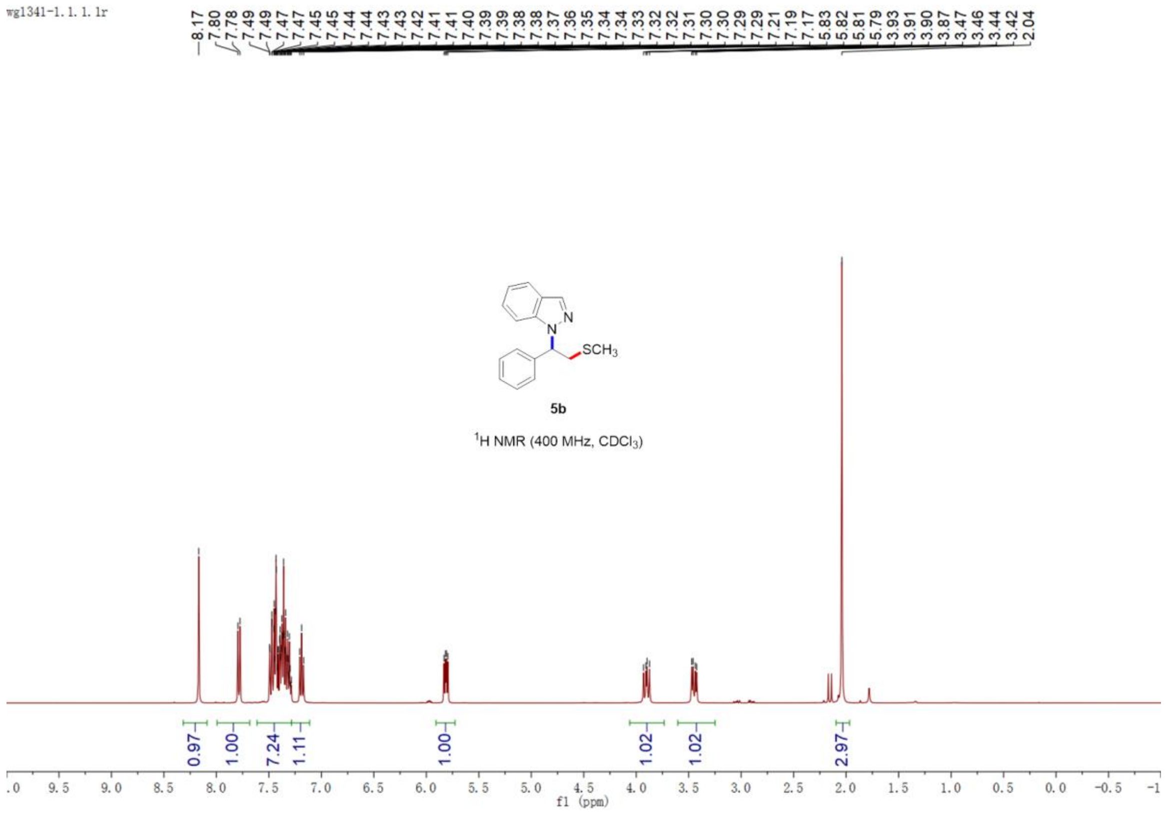
wg1341-1.1.1.1r
N
SCH3
5b
1H NMR (400 MHz, CDCl3)
0.97
1.00
7.24
1.11
1.00
1.02
1.02
2.97
f1 (ppm)

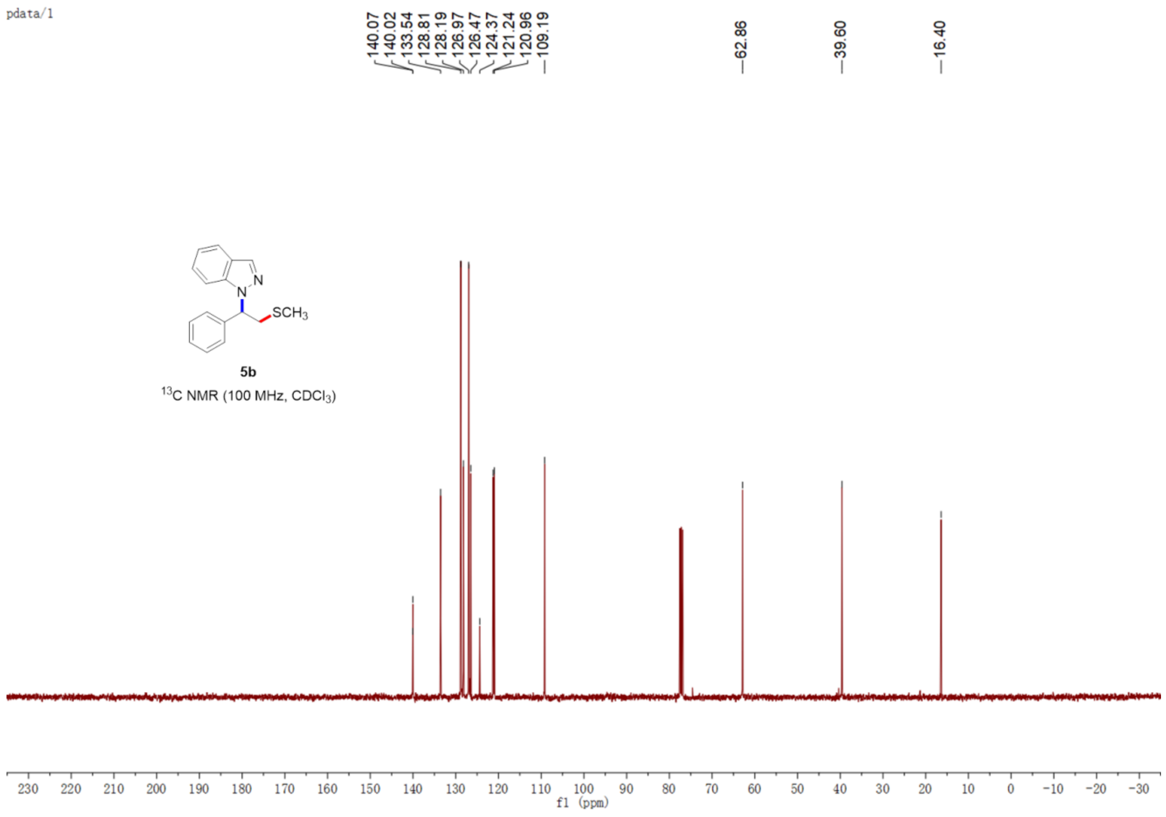
pdata/1
140.07
140.02
133.54
128.81
128.19
126.97
126.47
124.37
121.24
120.96
109.19
62.86
39.60
16.40
SCH3
5b
13C NMR (100 MHz, CDCl3)
f1 (ppm)

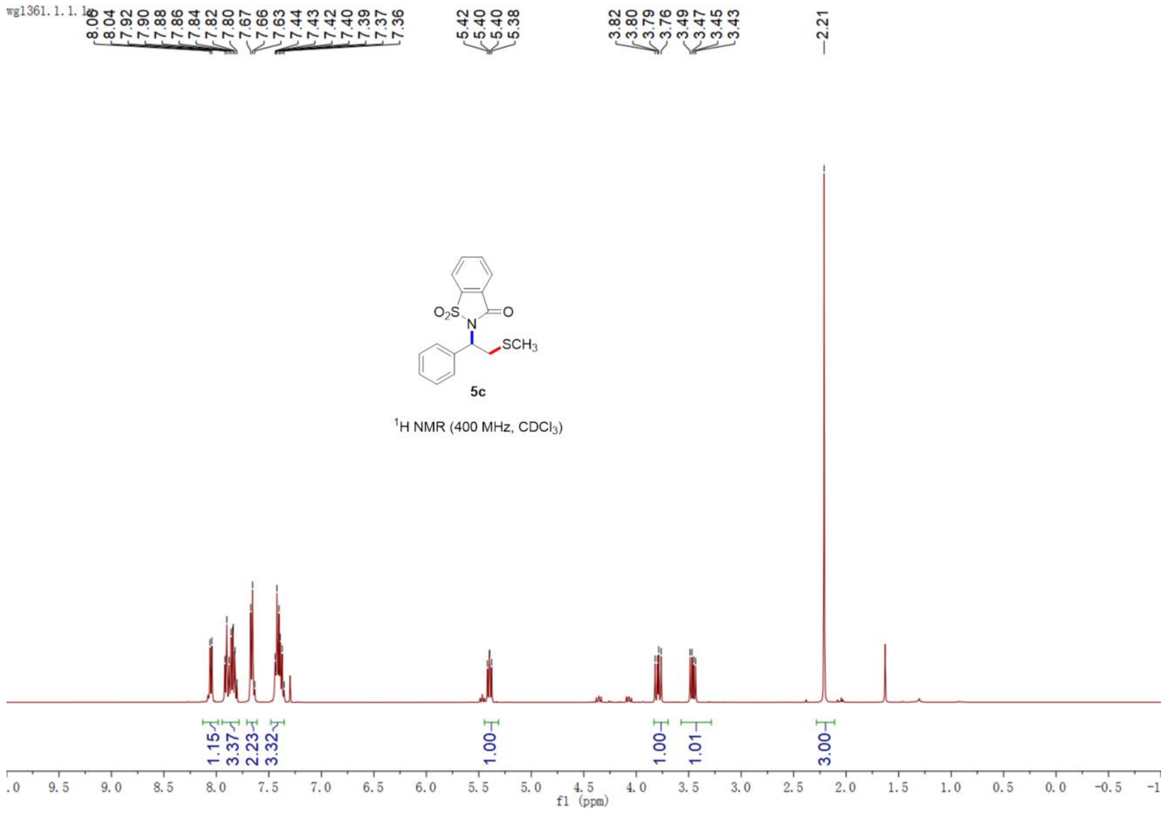
wg1361.1.1.1r
8.06
8.04
7.92
7.90
7.88
7.86
7.84
7.82
7.80
7.67
7.66
7.63
7.44
7.43
7.42
7.40
7.39
7.37
7.36
5.42
5.40
5.40
5.38
3.82
3.80
3.79
3.76
3.49
3.47
3.45
3.43
2.21
SCH3
5c
1H NMR (400 MHz, CDCl3)
1.15
3.37
2.23
3.32
1.00
1.00
1.01
3.00
f1 (ppm)

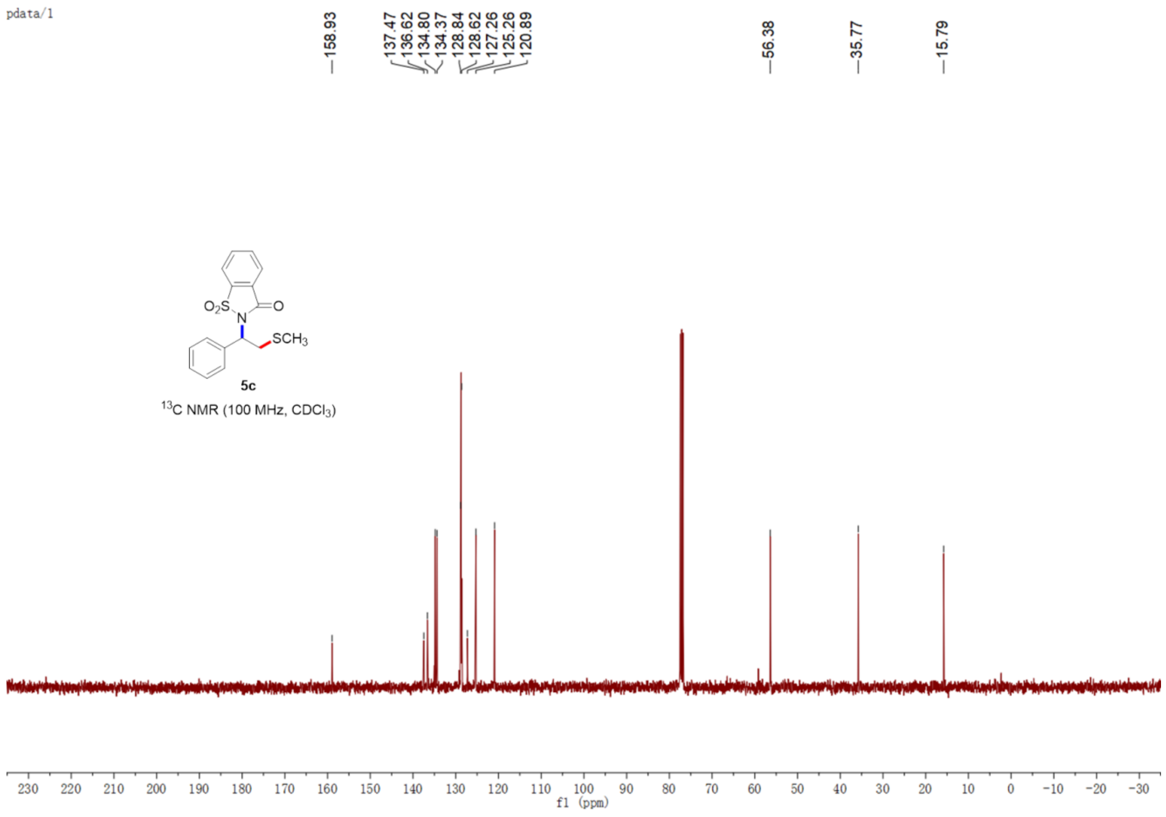
pdata/1
158.93
137.47
136.62
134.80
134.37
128.84
128.62
127.26
125.26
120.89
56.38
35.77
15.79
5c
13C NMR (100 MHz, CDCl3)
230 220 210 200 190 180 170 160 150 140 130 120 110 100 90 80 70 60 50 40 30 20 10 0 -10 -20 -30
f1 (ppm)

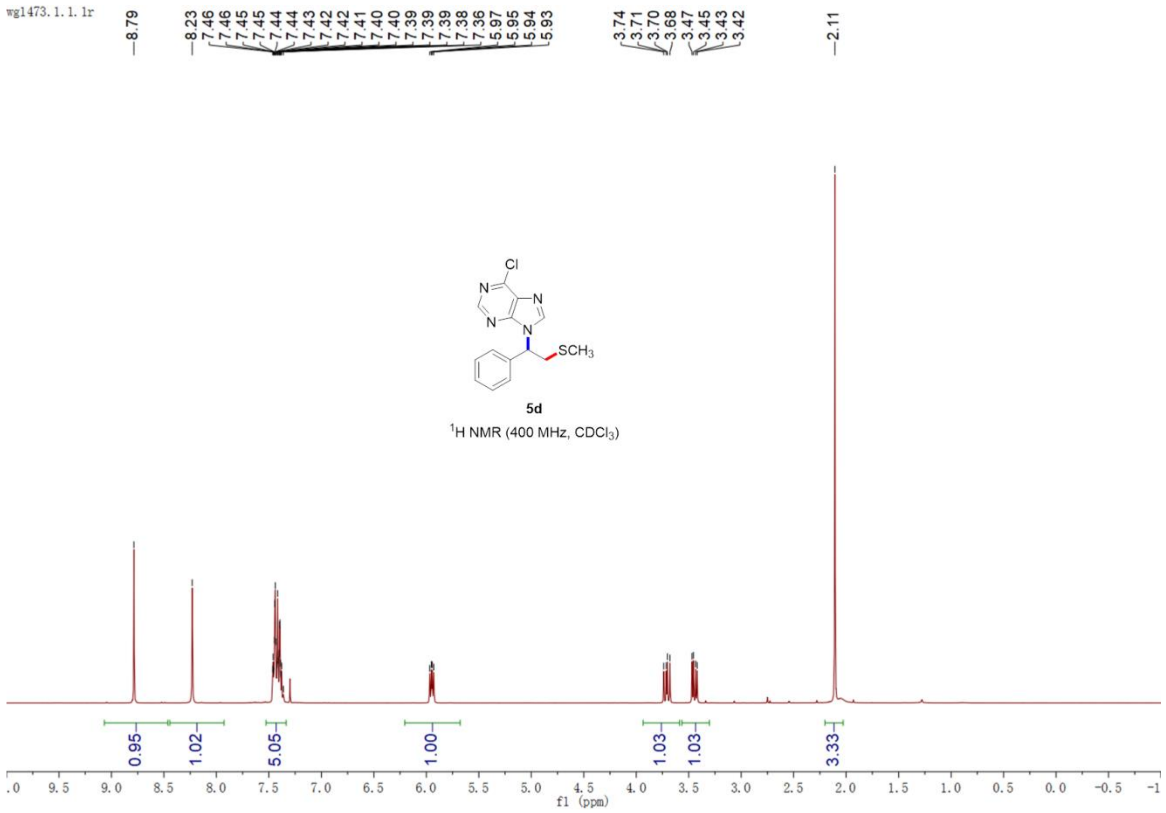
wg1473.1.1.1r
8.79
8.23
7.46
7.45
7.45
7.44
7.44
7.43
7.42
7.42
7.41
7.40
7.39
7.39
7.39
7.38
7.36
5.97
5.95
5.94
5.93
3.74
3.71
3.70
3.68
3.47
3.45
3.43
3.42
2.11
Cl
5d
1H NMR (400 MHz, CDCl3)
0.95
1.02
5.05
1.00
1.03
1.03
3.33
9.5 9.0 8.5 8.0 7.5 7.0 6.5 6.0 5.5 5.0 4.5 4.0 3.5 3.0 2.5 2.0 1.5 1.0 0.5 0.0 -0.5
f1 (ppm)

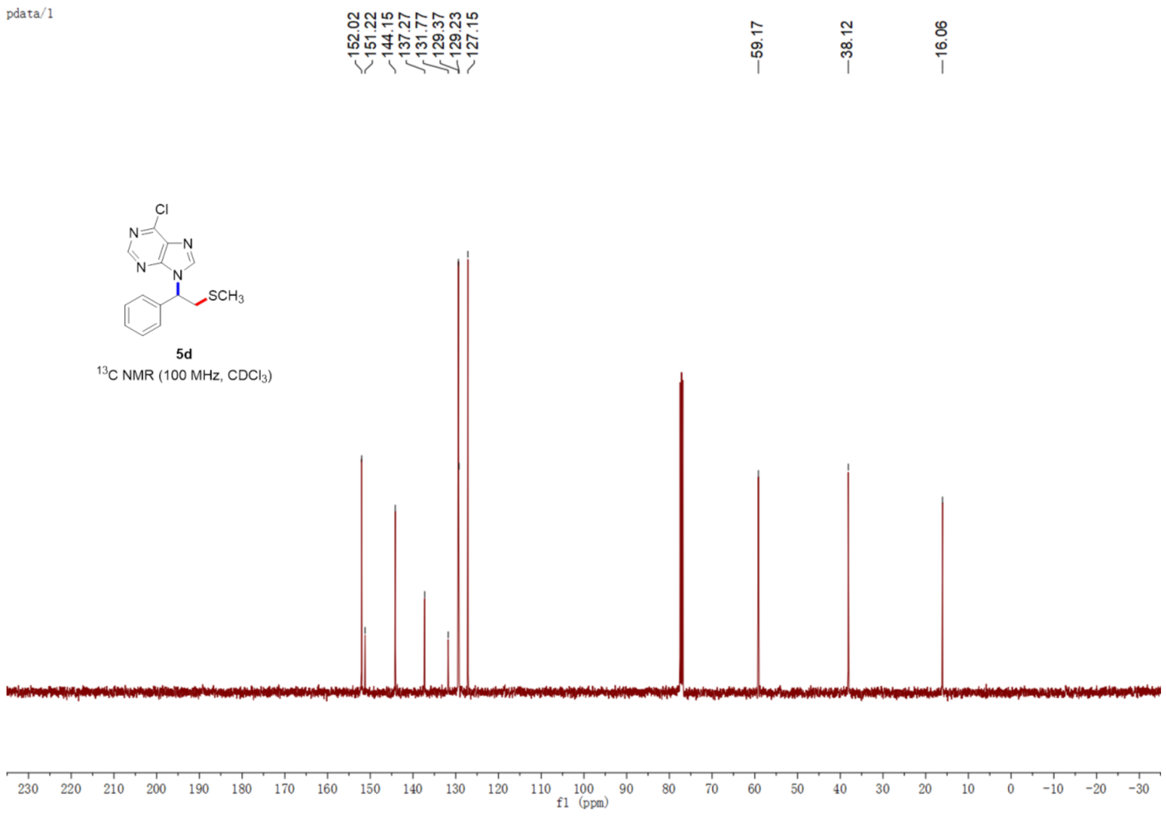

5d

^{13}C NMR (100 MHz, $CDCl_3$)

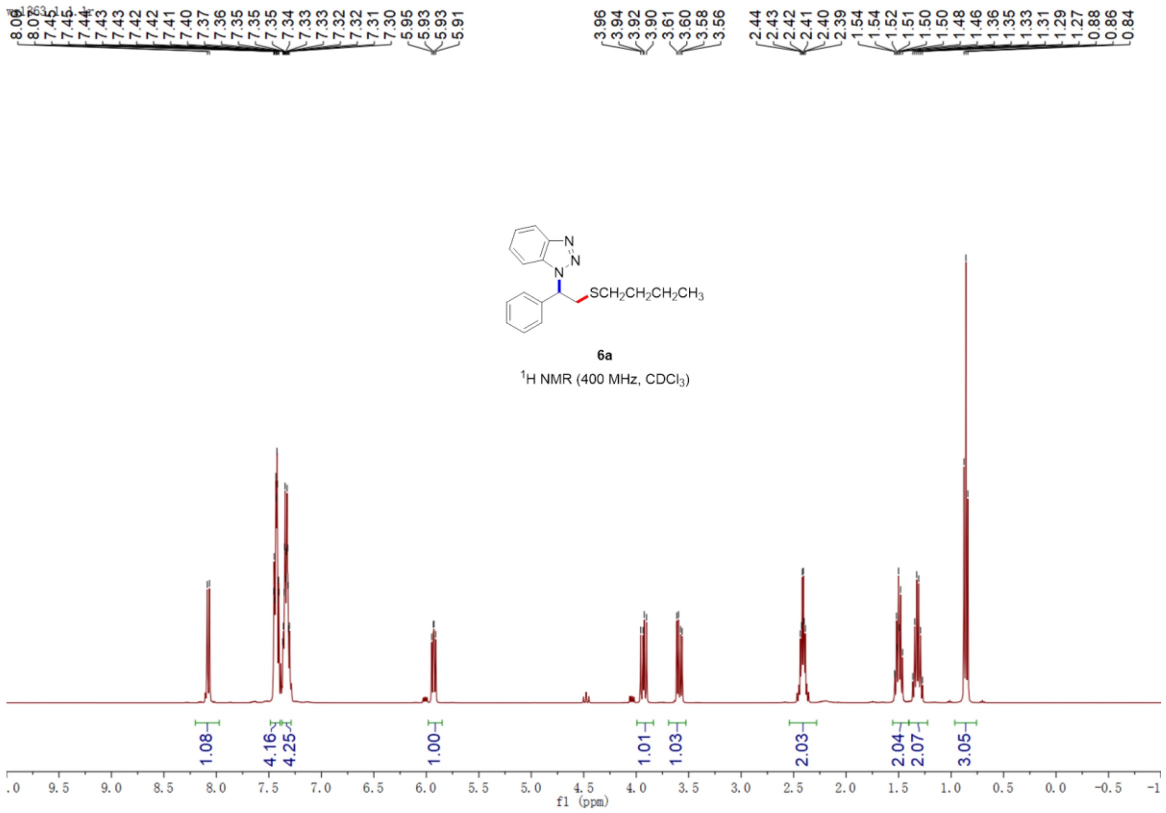

6a

^{1}H NMR (400 MHz, $CDCl_3$)

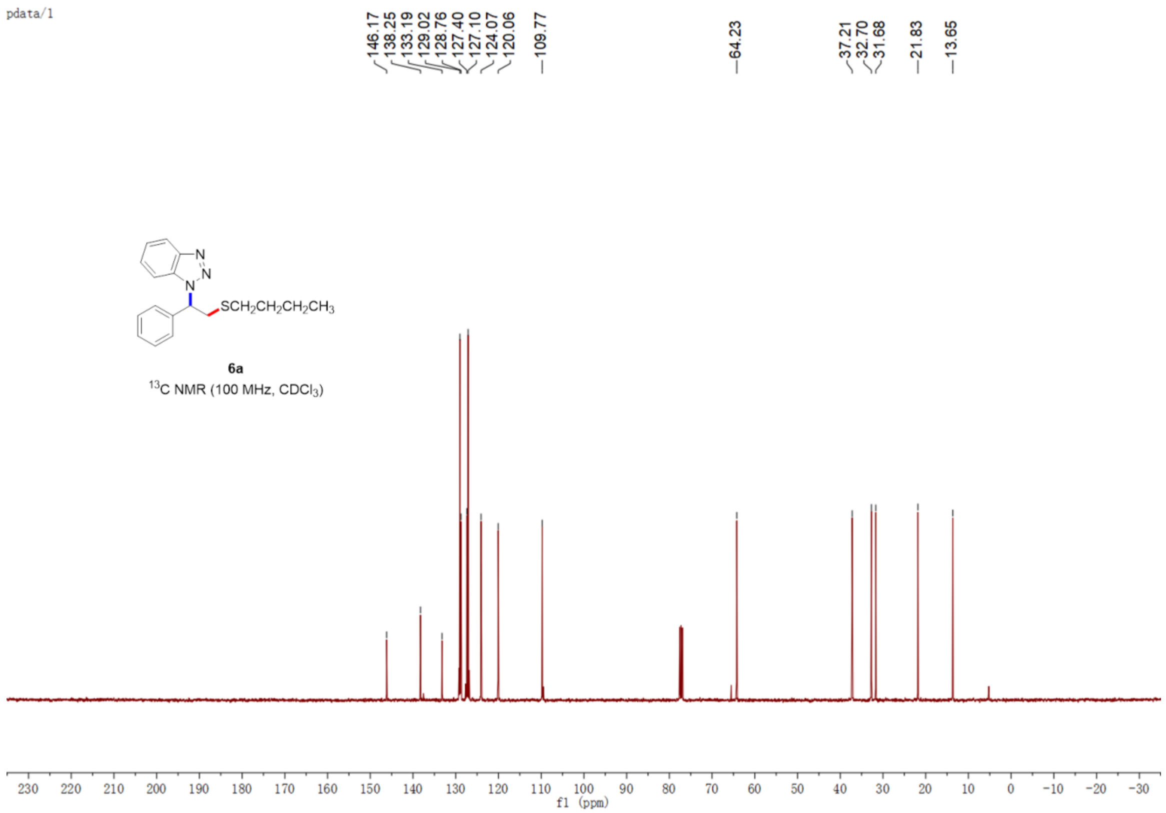
pdata/1
146.17
138.25
133.19
129.02
128.76
127.40
127.10
124.07
120.06
109.77
64.23
37.21
32.70
31.68
21.83
13.65
SCH2CH2CH2CH3
6a
13C NMR (100 MHz, CDCl3)
f1 (ppm)

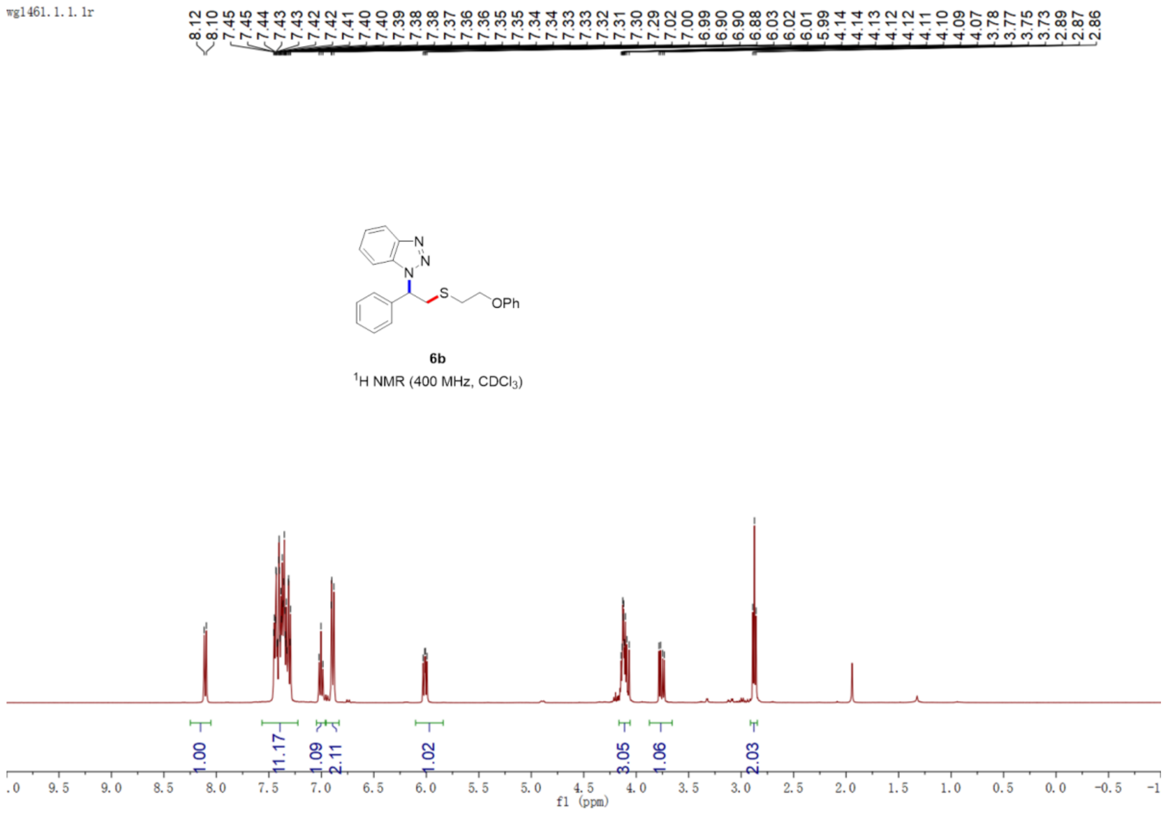
wg1461.1.1.1r
8.12
8.10
7.45
7.45
7.44
7.43
7.43
7.42
7.42
7.41
7.40
7.40
7.39
7.38
7.38
7.37
7.36
7.36
7.35
7.35
7.34
7.34
7.33
7.33
7.32
7.31
7.30
7.29
7.02
7.00
6.99
6.90
6.90
6.88
6.03
6.02
6.01
5.99
4.14
4.14
4.13
4.12
4.12
4.11
4.10
4.09
4.07
3.78
3.77
3.75
3.73
2.89
2.87
2.86
OPh
6b
1H NMR (400 MHz, CDCl3)
1.00
11.17
1.09
2.11
1.02
3.05
1.06
2.03
f1 (ppm)

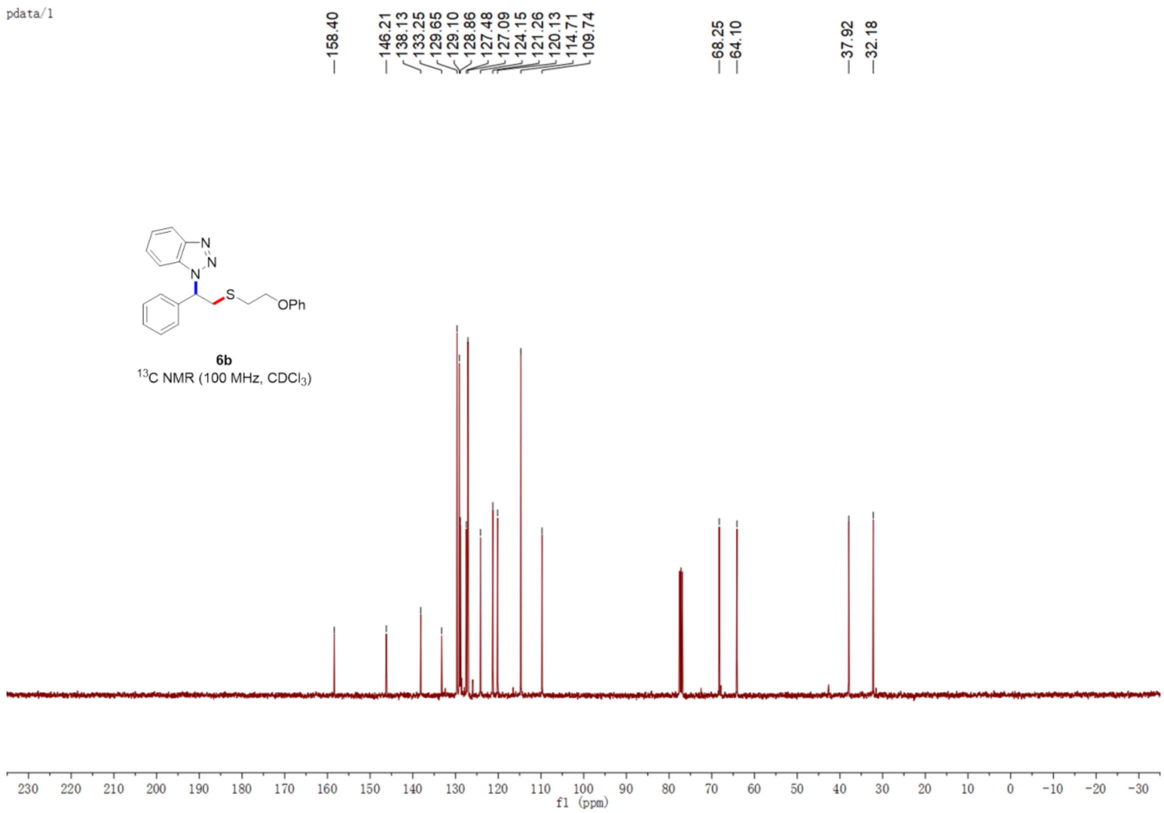
pdata/1
158.40
146.21
138.13
133.25
129.65
129.10
128.86
127.48
127.09
124.15
121.26
120.13
114.71
109.74
68.25
64.10
37.92
32.18
N
N
N
S
OPh
6b
^{13}C NMR (100 MHz, CDCl$_3$)
230 220 210 200 190 180 170 160 150 140 130 120 110 100 90 80 70 60 50 40 30 20 10 0 -10 -20 -30
f1 (ppm)

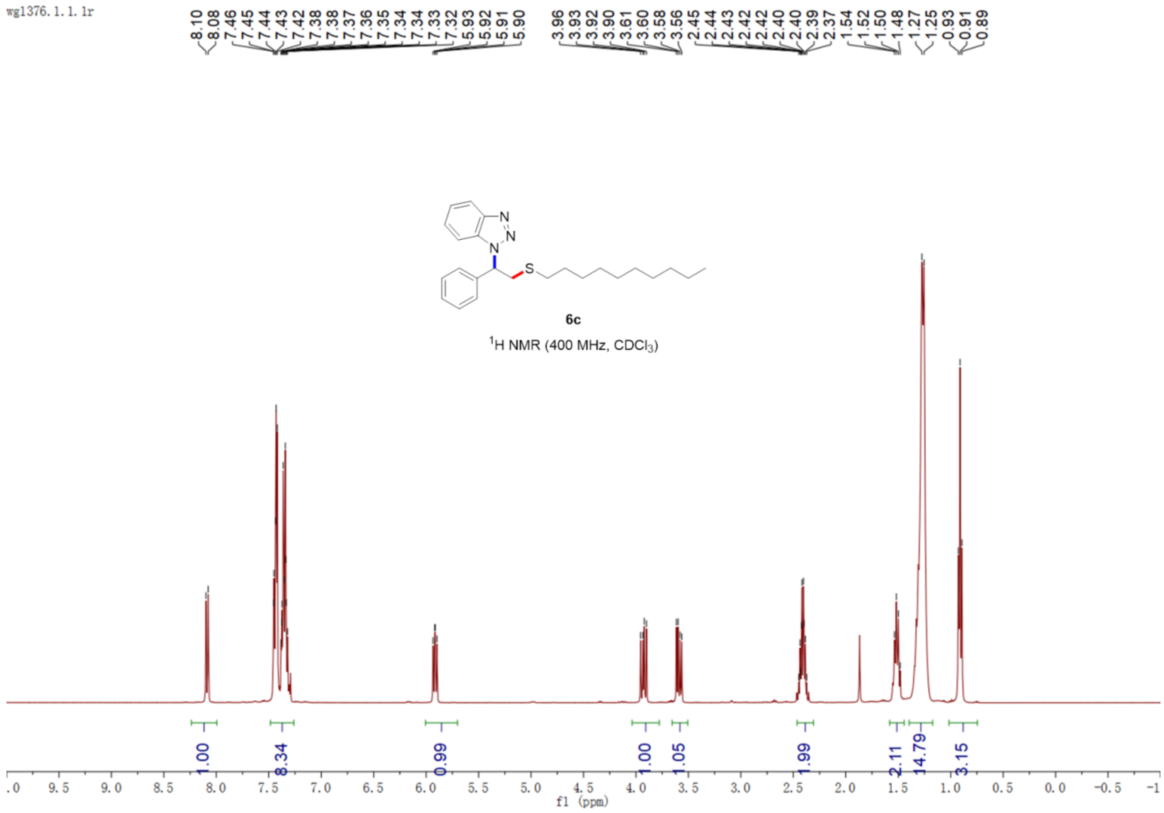
wg1376.1.1.1r
8.10
8.08
7.46
7.45
7.44
7.43
7.42
7.38
7.38
7.37
7.36
7.35
7.34
7.34
7.33
7.32
5.93
5.92
5.91
5.90
3.96
3.93
3.92
3.90
3.61
3.60
3.58
3.56
2.45
2.44
2.43
2.42
2.42
2.40
2.40
2.39
2.37
1.54
1.52
1.50
1.48
1.27
1.25
0.93
0.91
0.89
N
N
N
S
6c
^{1}H NMR (400 MHz, CDCl$_3$)
1.00
8.34
0.99
1.00
1.05
1.99
2.11
14.79
3.15
.0 9.5 9.0 8.5 8.0 7.5 7.0 6.5 6.0 5.5 5.0 4.5 4.0 3.5 3.0 2.5 2.0 1.5 1.0 0.5 0.0 -0.5 -1
f1 (ppm)

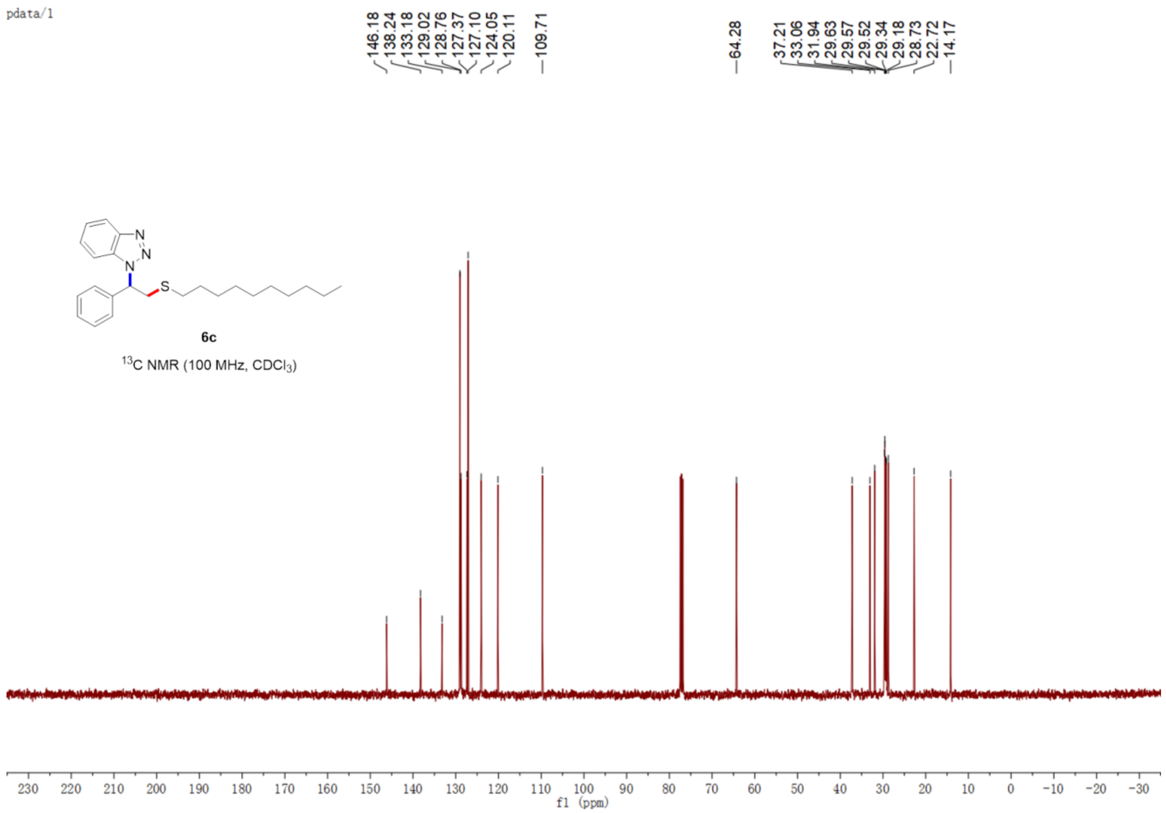
pdata/1
146.18
138.24
133.18
129.02
128.76
127.37
127.10
124.05
120.11
109.71
64.28
37.21
33.06
31.94
29.63
29.57
29.52
29.34
29.18
28.73
22.72
14.17
6c
^{13}C NMR (100 MHz, CDCl$_3$)
f1 (ppm)

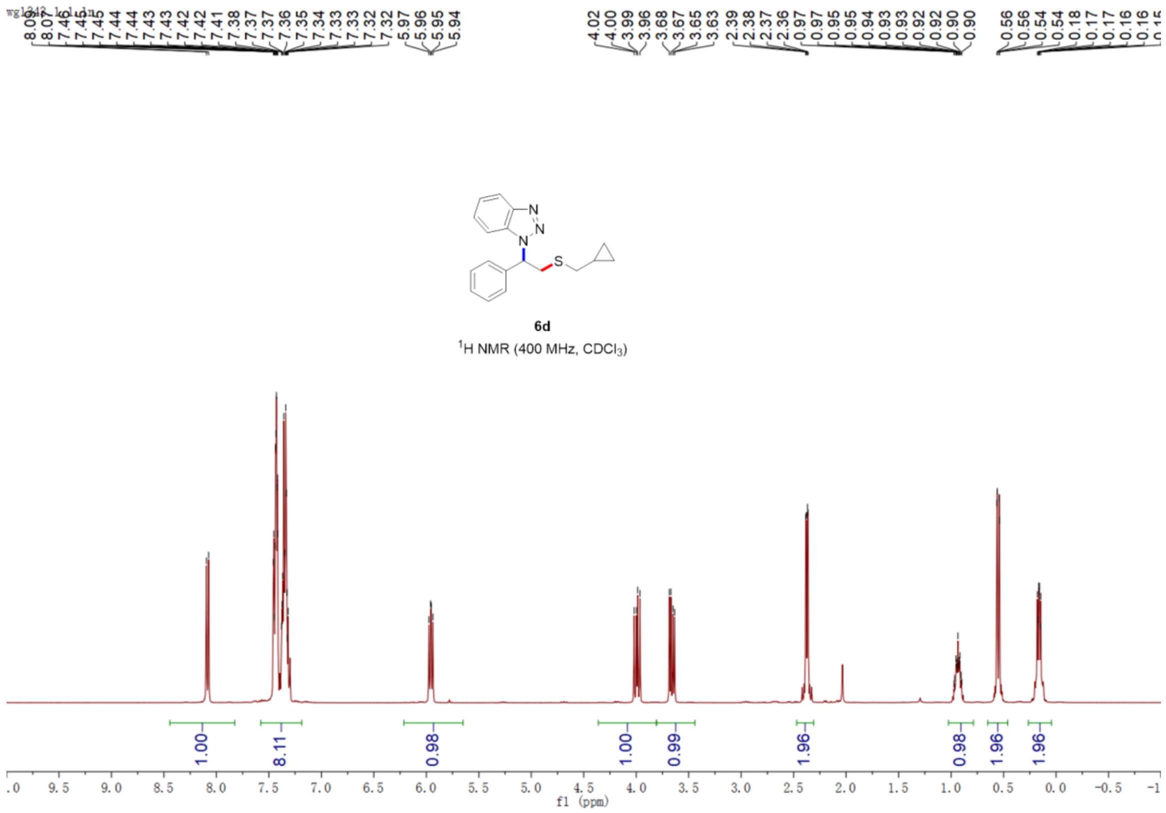
7.46
7.45
7.45
7.44
7.44
7.43
7.42
7.42
7.41
7.38
7.37
7.36
7.35
7.34
7.33
7.33
7.32
7.32
5.97
5.96
5.95
5.94
4.02
4.00
3.99
3.96
3.68
3.67
3.65
3.63
2.39
2.38
2.37
2.36
0.97
0.97
0.95
0.95
0.94
0.93
0.93
0.92
0.92
0.90
0.90
0.56
0.56
0.54
0.54
0.18
0.17
0.17
0.16
0.16
6d
^{1}H NMR (400 MHz, CDCl$_3$)
1.00
8.11
0.98
1.00
0.99
1.96
0.98
1.96
1.96
f1 (ppm)

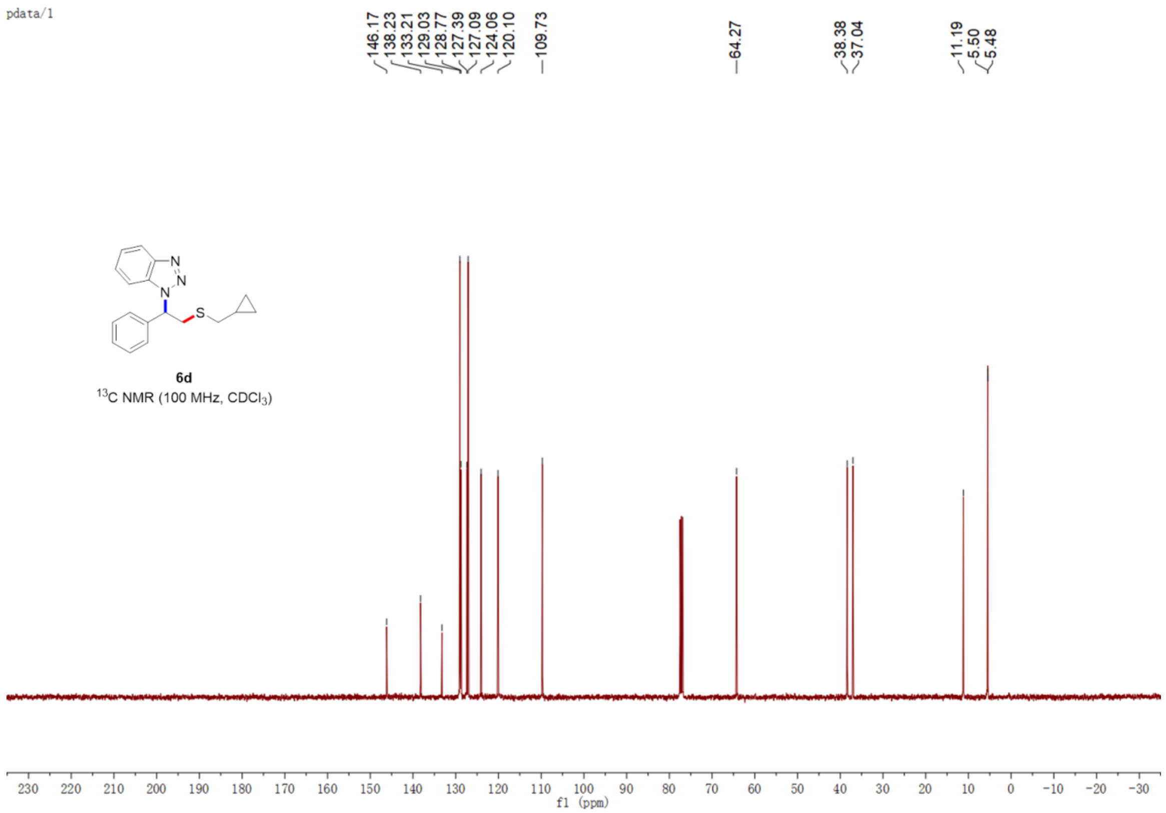
pdata/1
146.17
138.23
133.21
129.03
128.77
127.39
127.09
124.06
120.10
109.73
64.27
38.38
37.04
11.19
5.50
5.48
6d
^{13}C NMR (100 MHz, $CDCl_3$)
f1 (ppm)

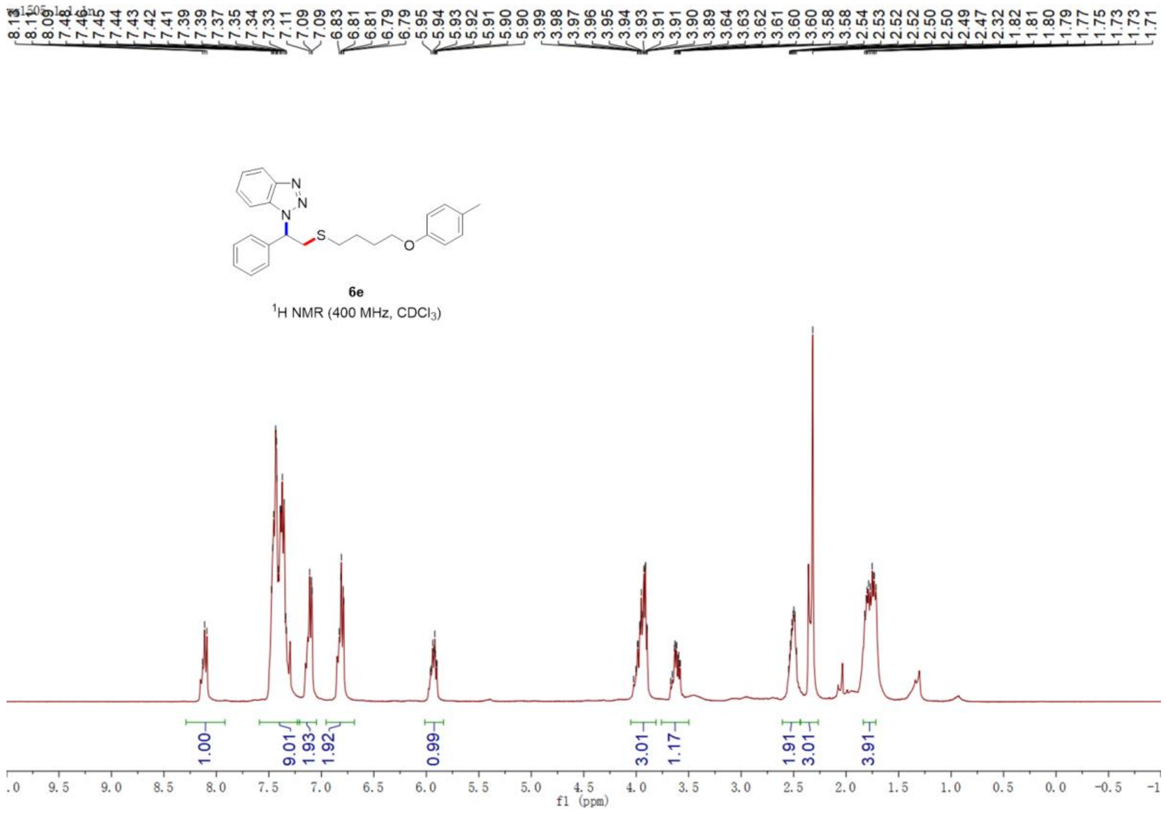
6e
^{1}H NMR (400 MHz, $CDCl_3$)
1.00
9.01
1.93
1.92
0.99
3.01
1.17
1.91
3.01
3.91
f1 (ppm)

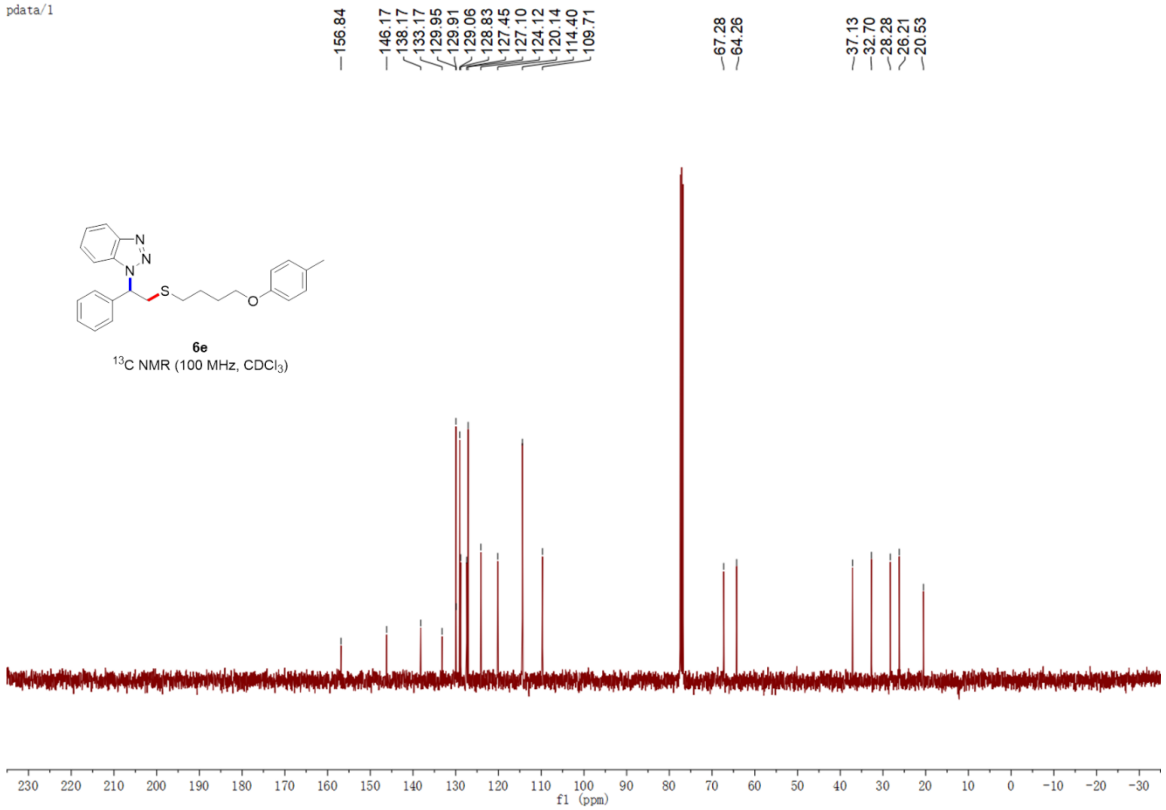

6e

^{13}C NMR (100 MHz, $CDCl_3$)

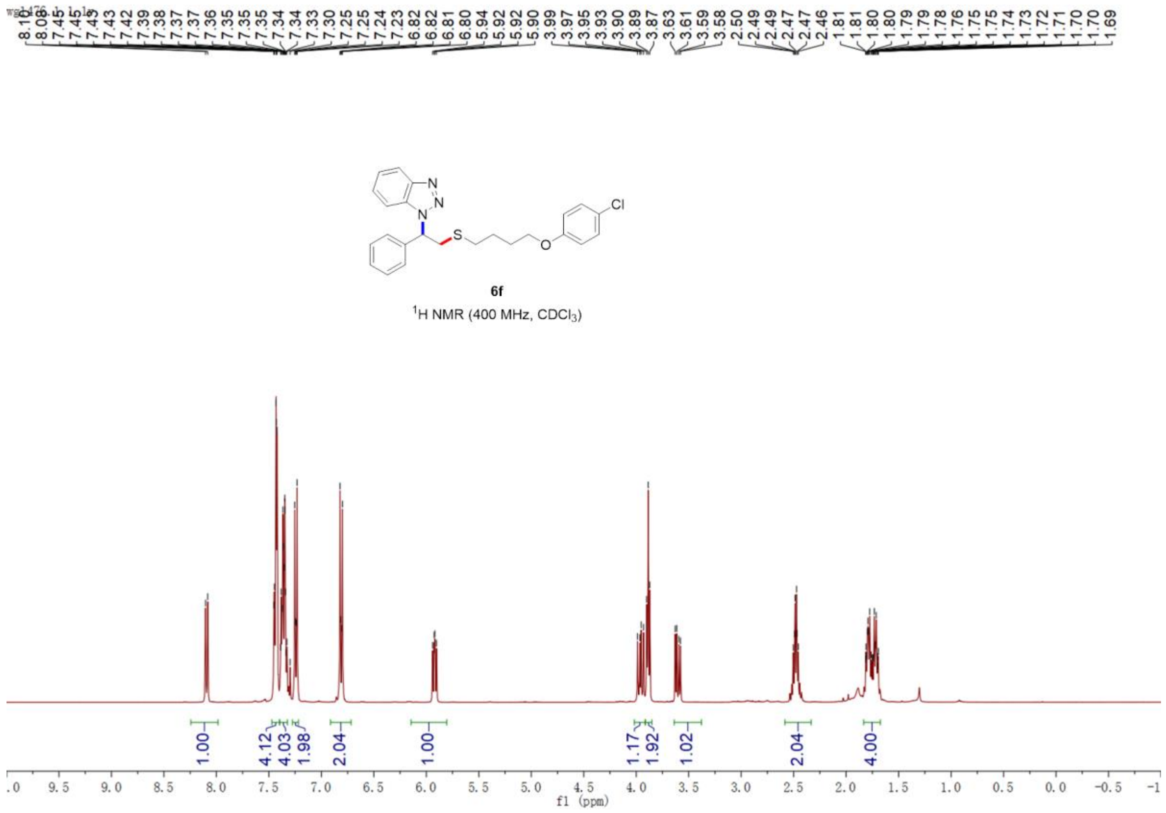

6f

^{1}H NMR (400 MHz, $CDCl_3$)

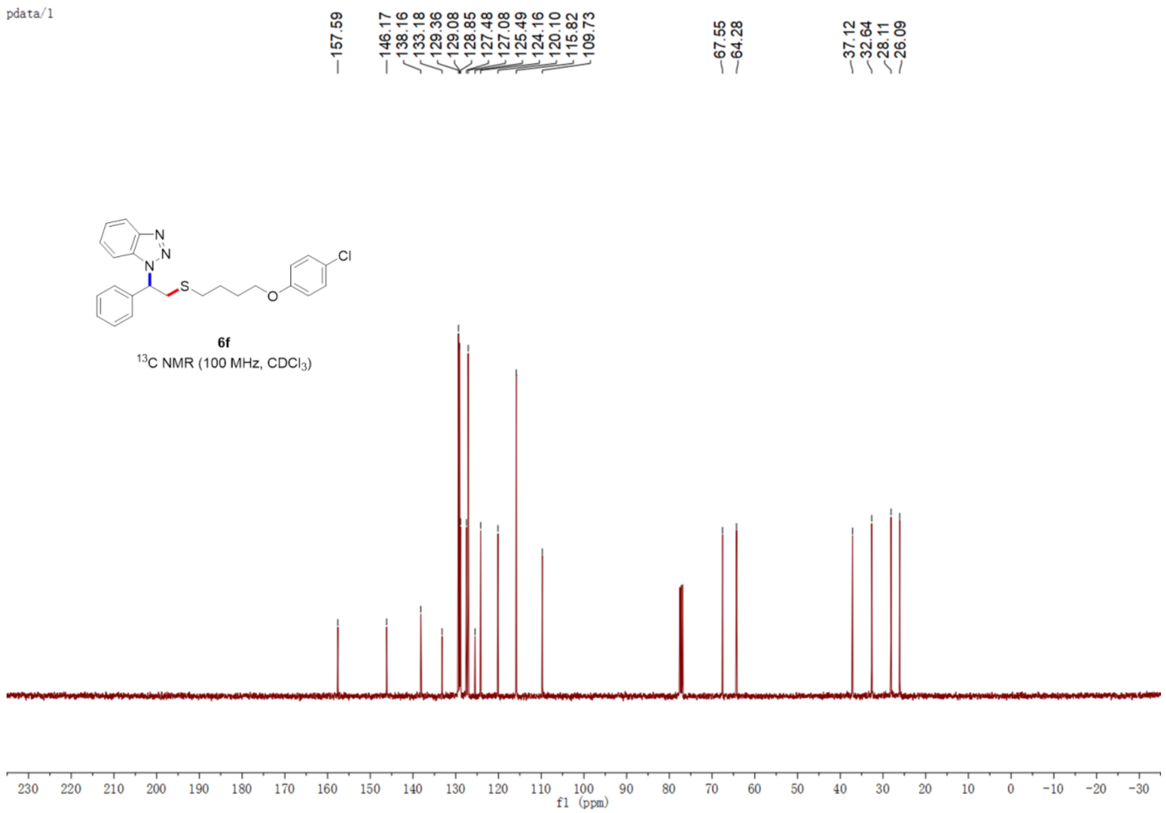
pdata/1
157.59
146.17
138.16
133.18
129.36
129.08
128.85
127.48
127.08
125.49
124.16
120.10
115.82
109.73
67.55
64.28
37.12
32.64
28.11
26.09
N
N
N
S
O
Cl
6f
^{13}C NMR (100 MHz, CDCl$_3$)
f1 (ppm)

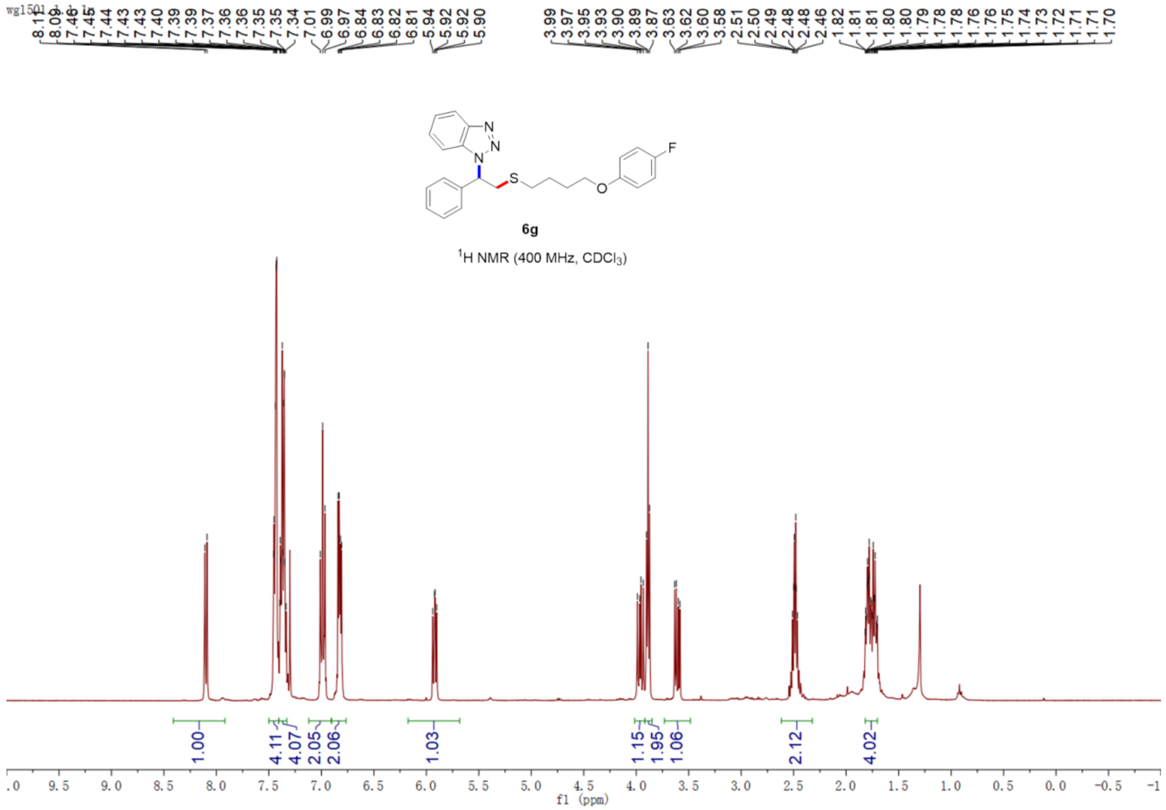
8.11
8.09
7.46
7.45
7.44
7.43
7.43
7.40
7.39
7.39
7.37
7.36
7.36
7.35
7.35
7.34
7.01
6.99
6.97
6.84
6.83
6.82
6.81
5.94
5.92
5.92
5.90
3.99
3.97
3.95
3.93
3.90
3.89
3.87
3.63
3.62
3.60
3.58
2.51
2.50
2.49
2.48
2.48
2.46
1.82
1.81
1.81
1.80
1.80
1.79
1.78
1.78
1.76
1.76
1.75
1.74
1.73
1.72
1.71
1.71
1.70
N
N
N
S
O
F
6g
^{1}H NMR (400 MHz, CDCl$_3$)
1.00
4.11
4.07
2.05
2.06
1.03
1.15
1.95
1.06
2.12
4.02
f1 (ppm)

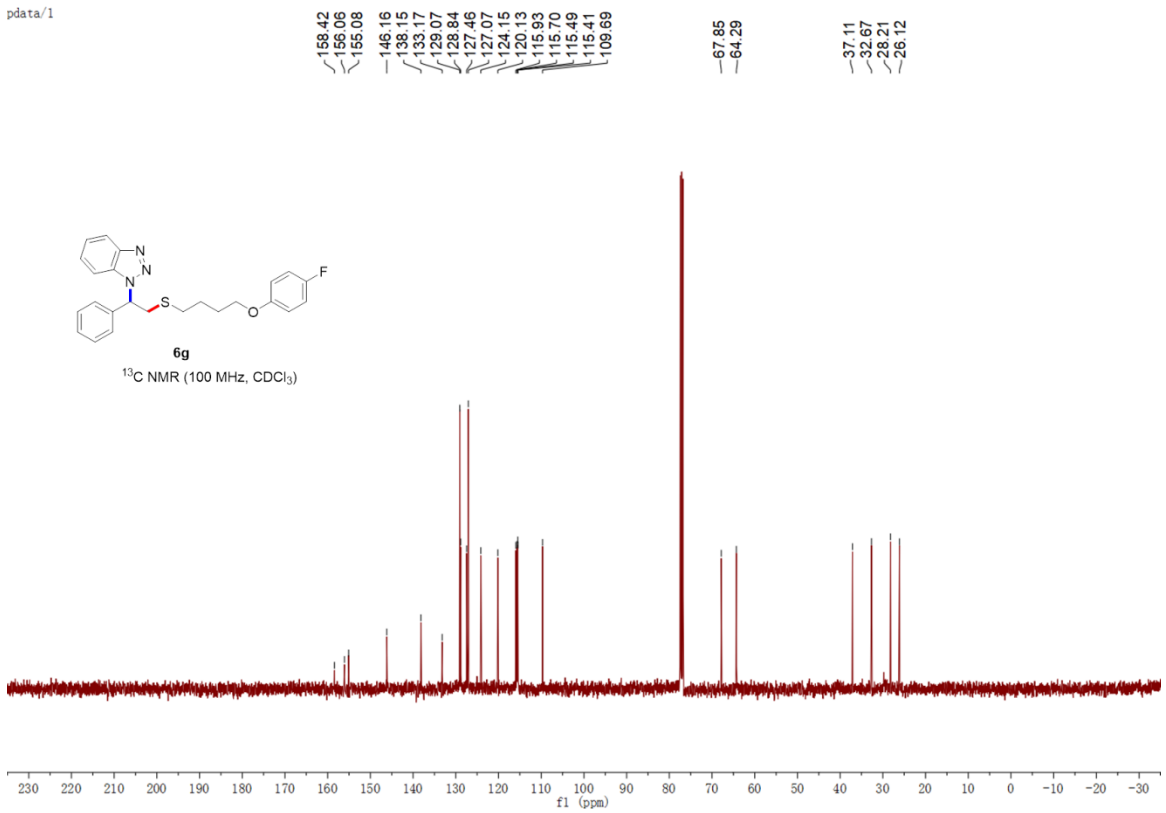
pdata/1
158.42
156.06
155.08
146.16
138.15
133.17
129.07
128.84
127.46
127.07
124.15
120.13
115.93
115.70
115.49
115.41
109.69
67.85
64.29
37.11
32.67
28.21
26.12
6g
^{13}C NMR (100 MHz, $CDCl_3$)
230 220 210 200 190 180 170 160 150 140 130 120 110 100 90 80 70 60 50 40 30 20 10 0 -10 -20 -30
f1 (ppm)

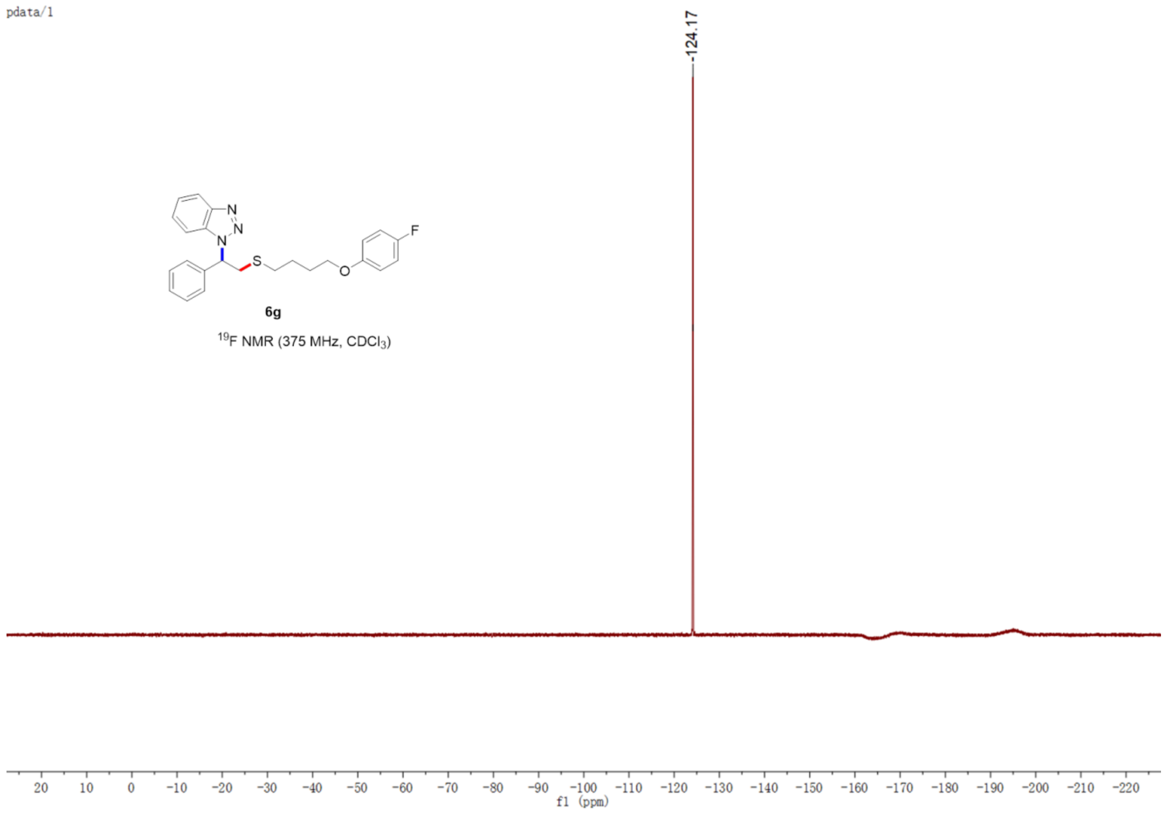
pdata/1
-124.17
6g
^{19}F NMR (375 MHz, $CDCl_3$)
20 10 0 -10 -20 -30 -40 -50 -60 -70 -80 -90 -100 -110 -120 -130 -140 -150 -160 -170 -180 -190 -200 -210 -220
f1 (ppm)

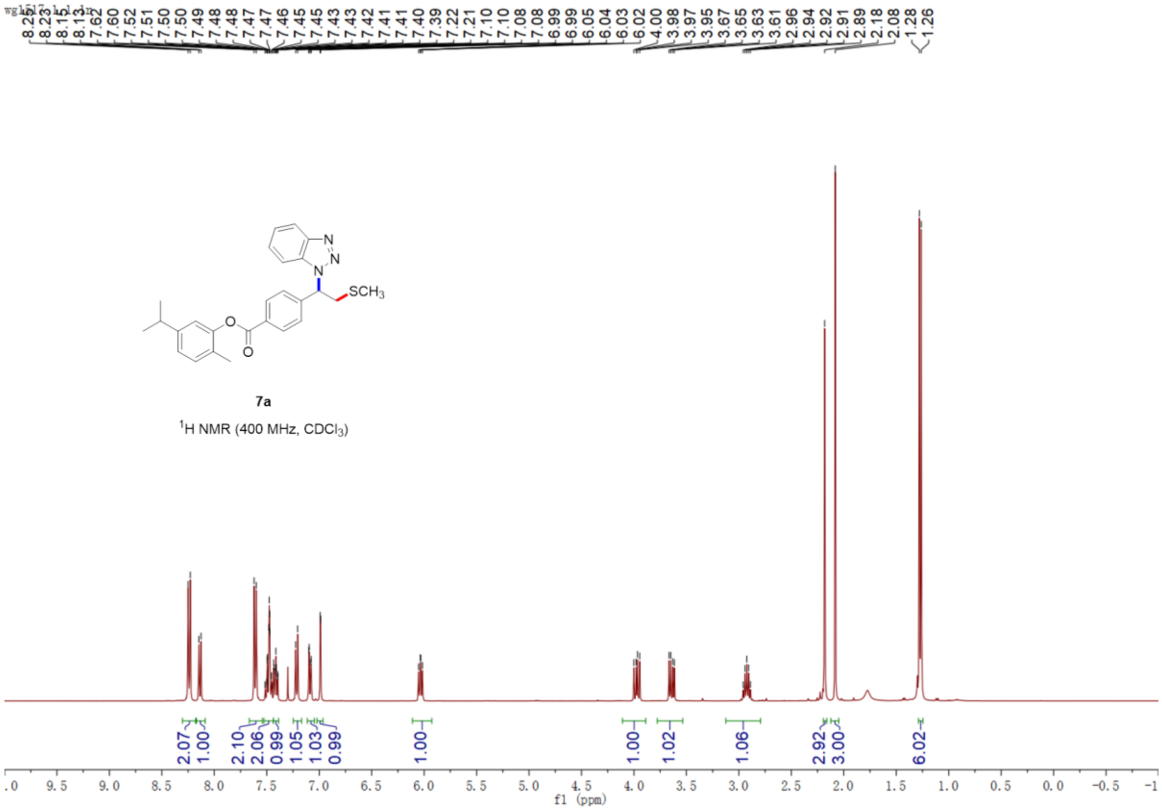

7a

^{1}H NMR (400 MHz, $CDCl_3$)

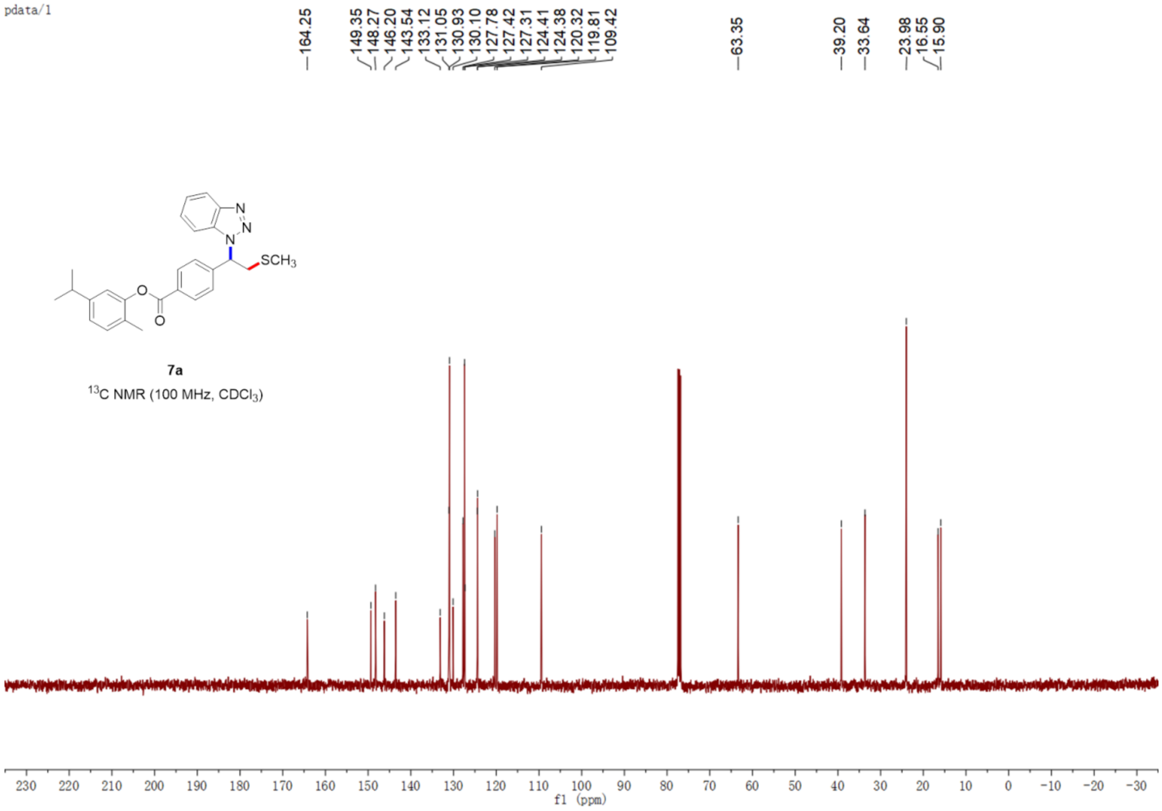

7a

^{13}C NMR (100 MHz, $CDCl_3$)

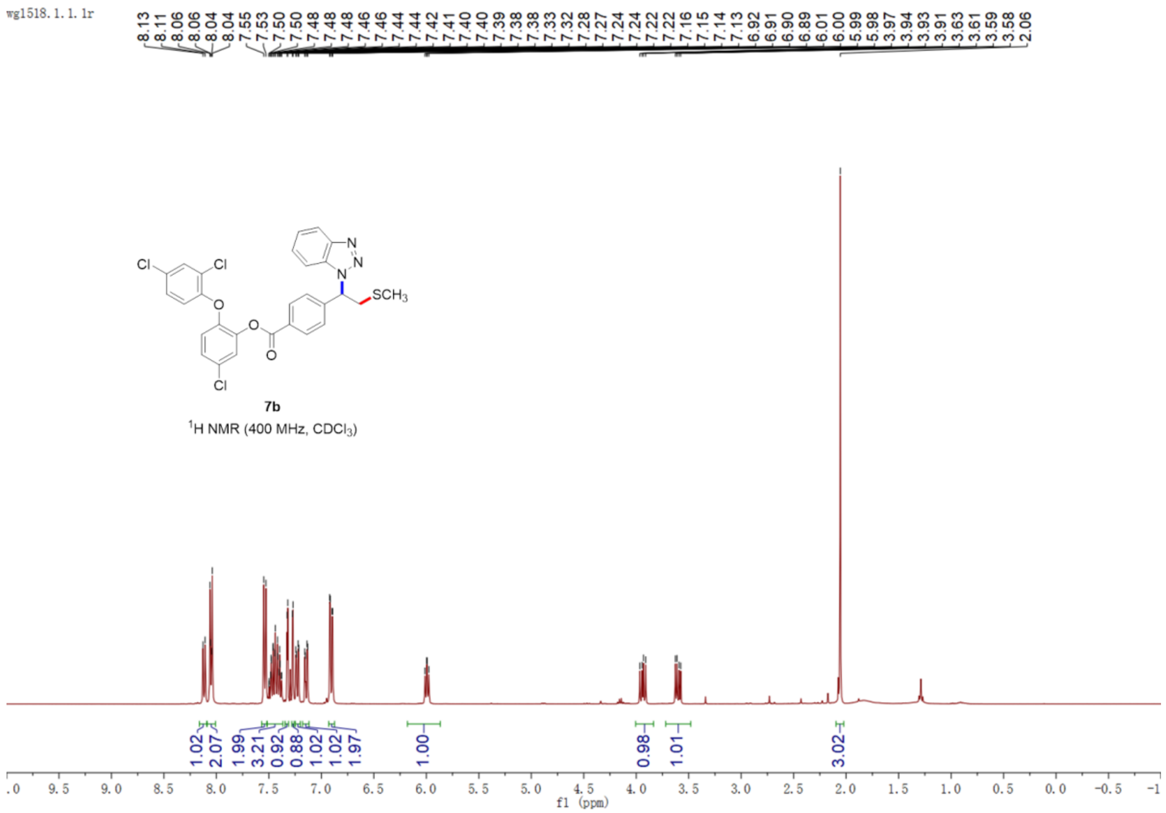

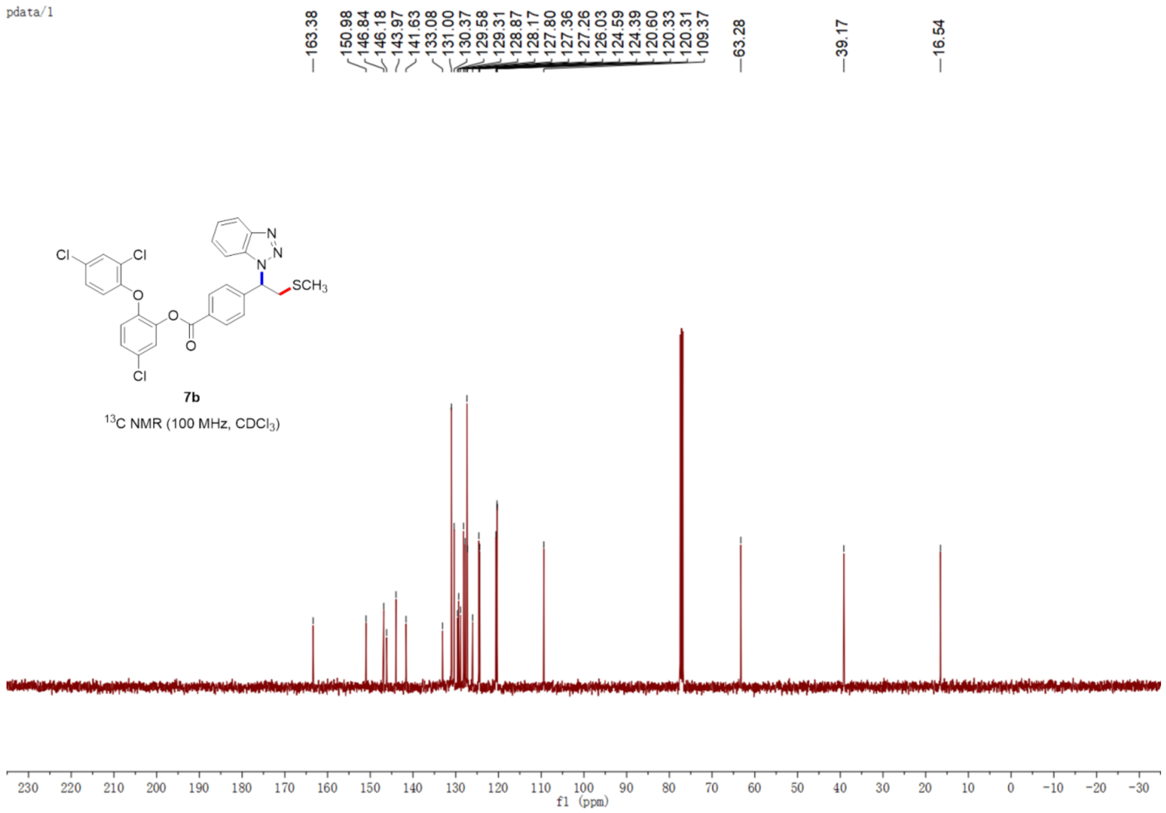

HRMS of products

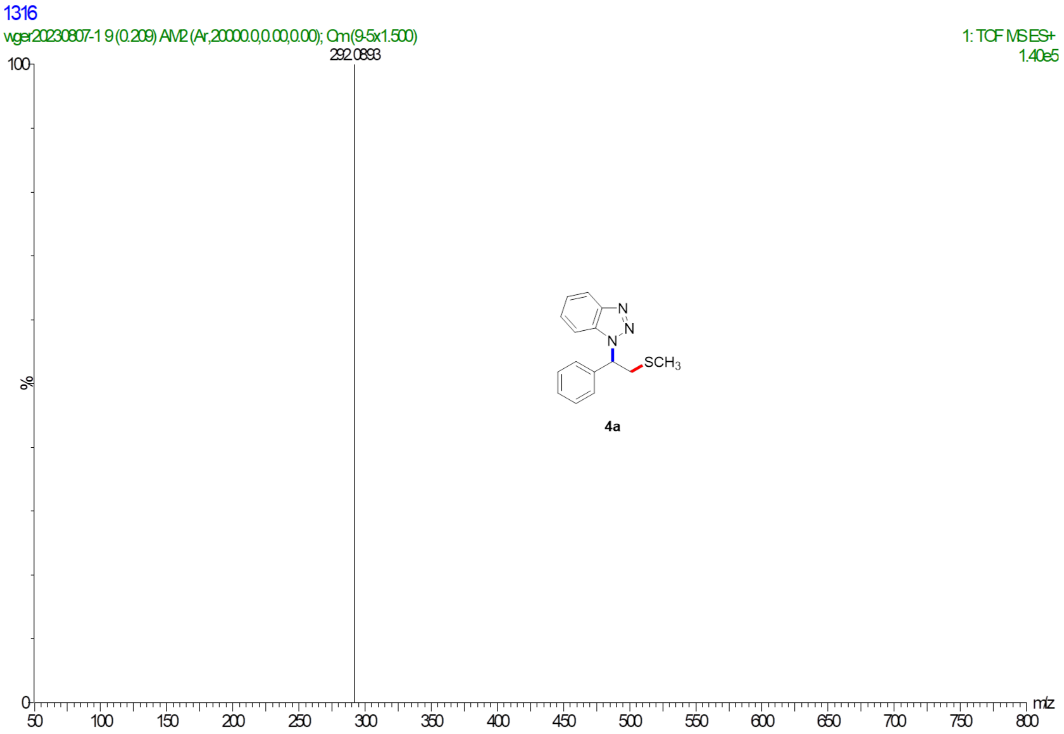

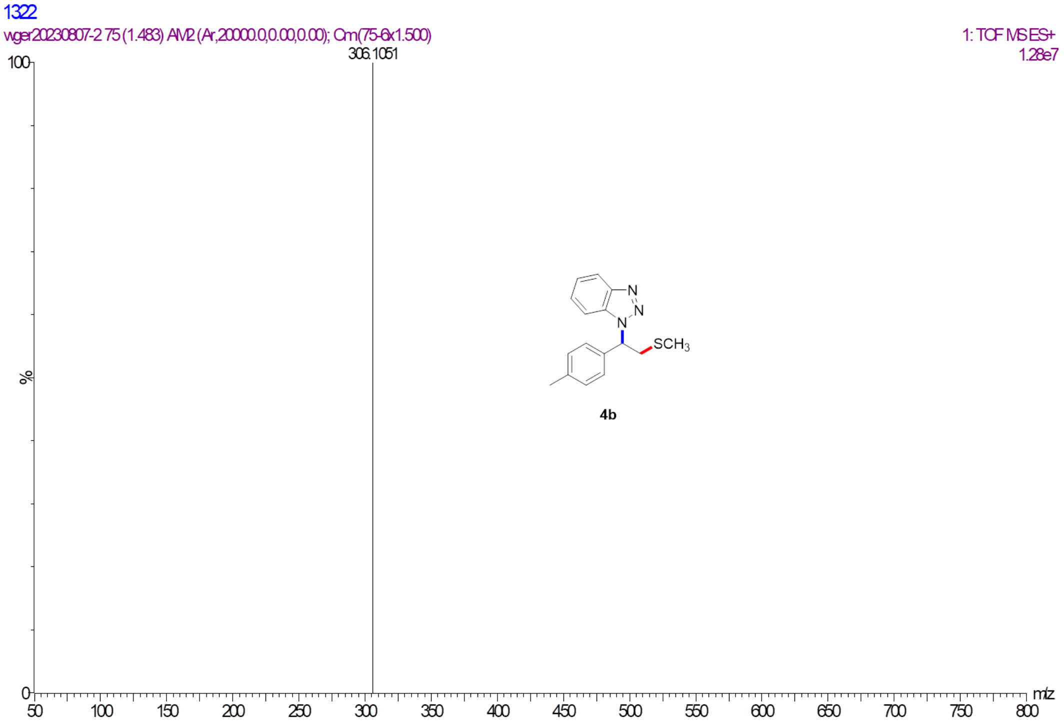

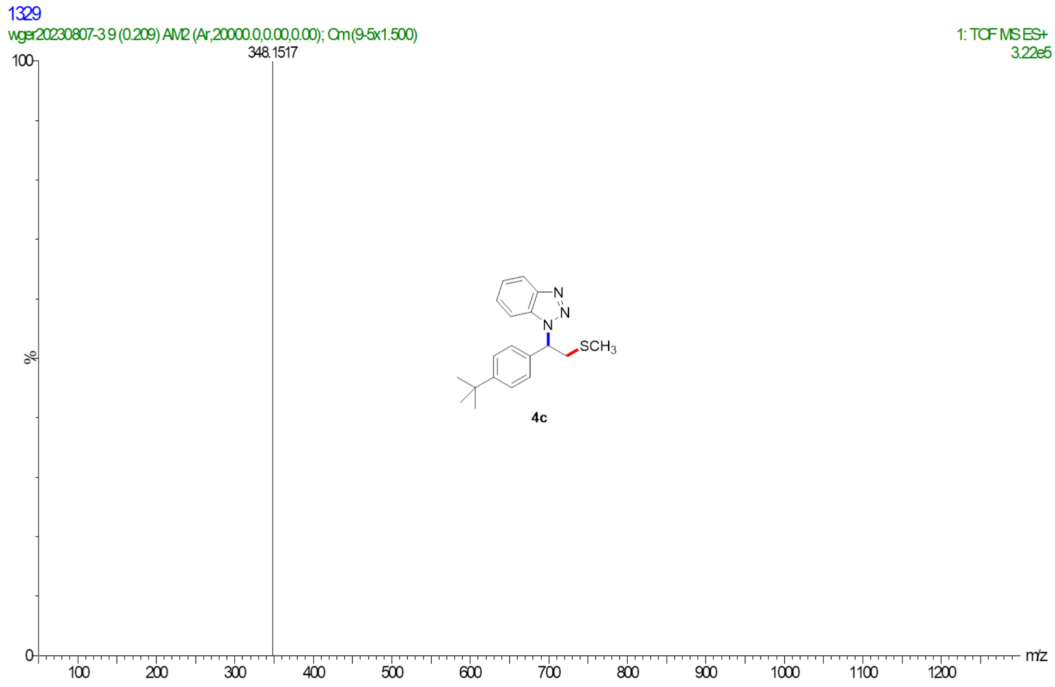
1329
wger20230807-3 9 (0.209) AM2 (Ar,20000.0,0.00,0.00); Cm (9-5x1.500)
1: TOF MS ES+
3.22e5
348.1517
100
%
0
m/z
100
200
300
400
500
600
700
800
900
1000
1100
1200
N
N
N
SCH3
4c

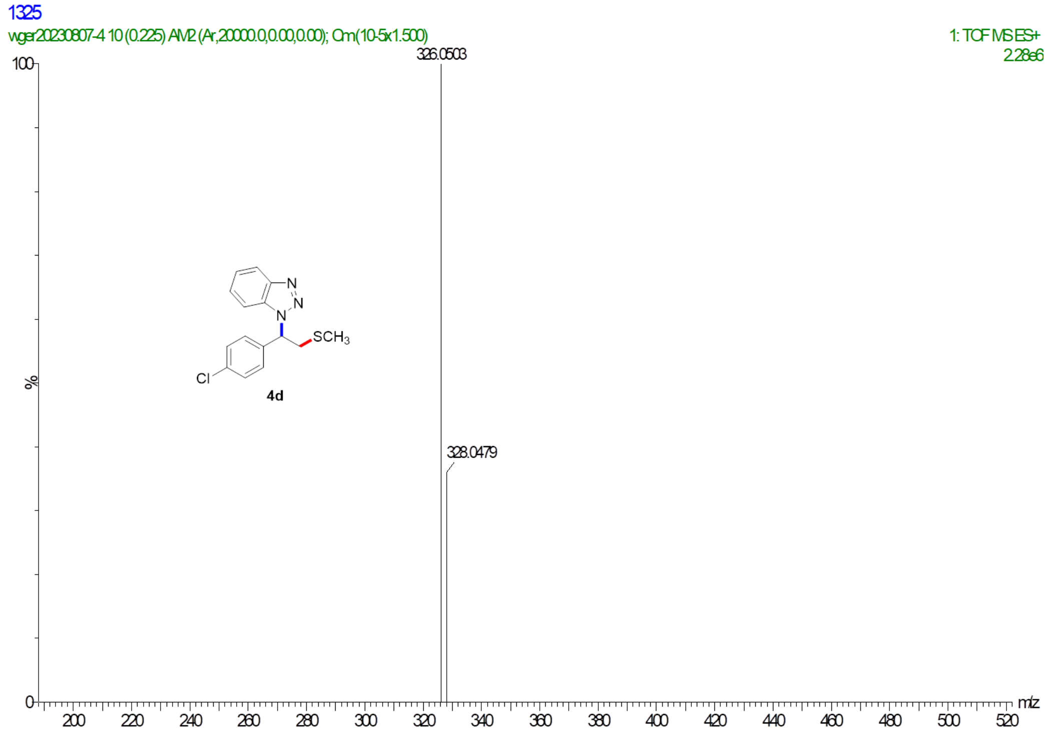
1325
wger20230807-4 10 (0.225) AM2 (Ar,20000.0,0.00,0.00); Cm (10-5x1.500)
1: TOF MS ES+
2.28e6
326.0503
328.0479
100
%
0
m/z
200
220
240
260
280
300
320
340
360
380
400
420
440
460
480
500
520
N
N
N
SCH3
Cl
4d

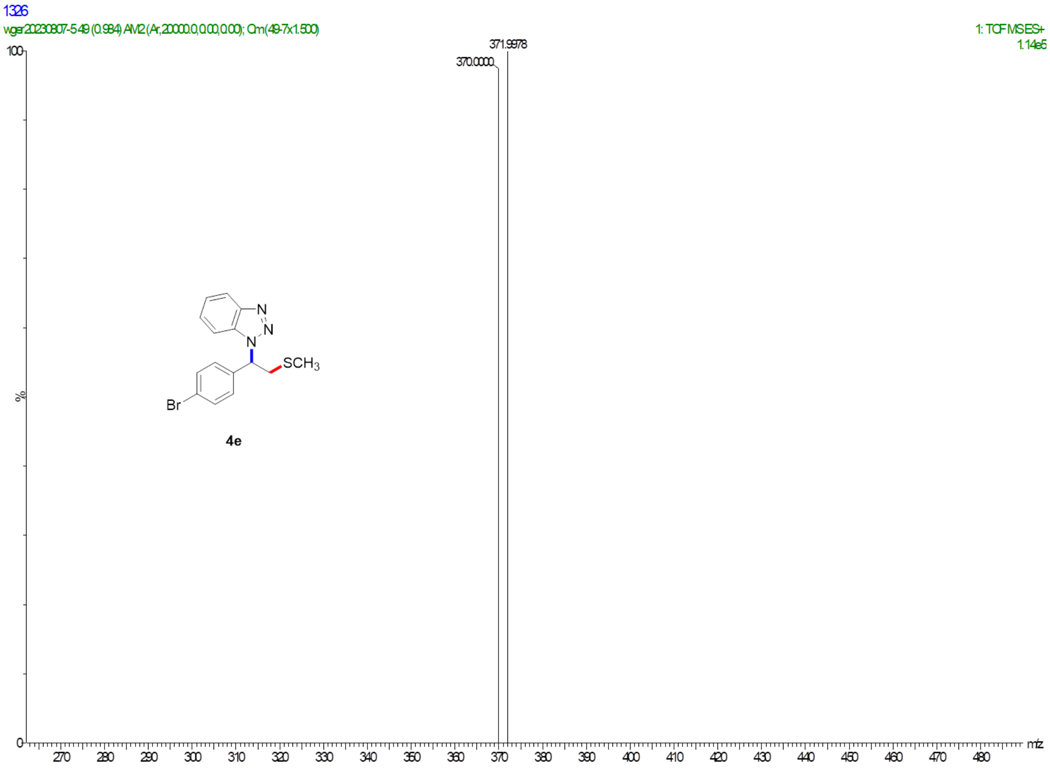
1326
wger20230807-5 49 (0.984) AM2 (Ar,20000.0,0.00,0.00); Cm (49-7x1.500)
1: TOF MS ES+
1.14e5
371.9978
370.0000
100
%
0
m/z
SCH$_3$
Br
N
4e

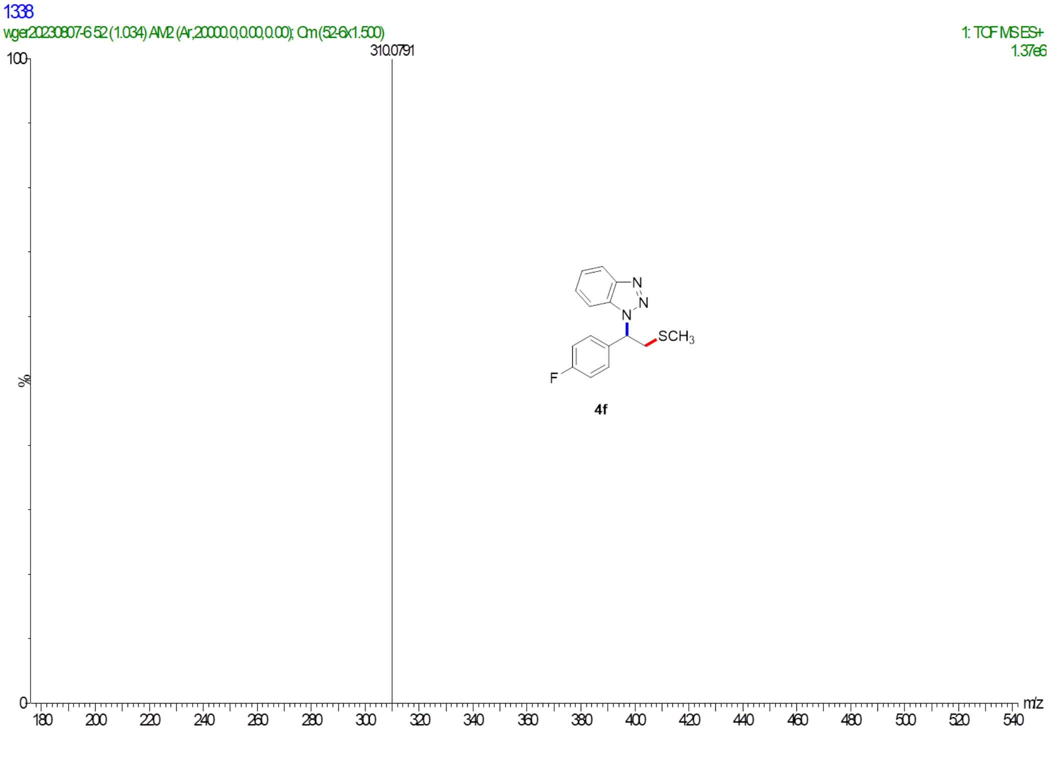
1338
wger20230807-6 52 (1.034) AM2 (Ar,20000.0,0.00,0.00); Cm (52-6x1.500)
1: TOF MS ES+
1.37e6
310.0791
100
%
0
m/z
SCH$_3$
F
N
4f

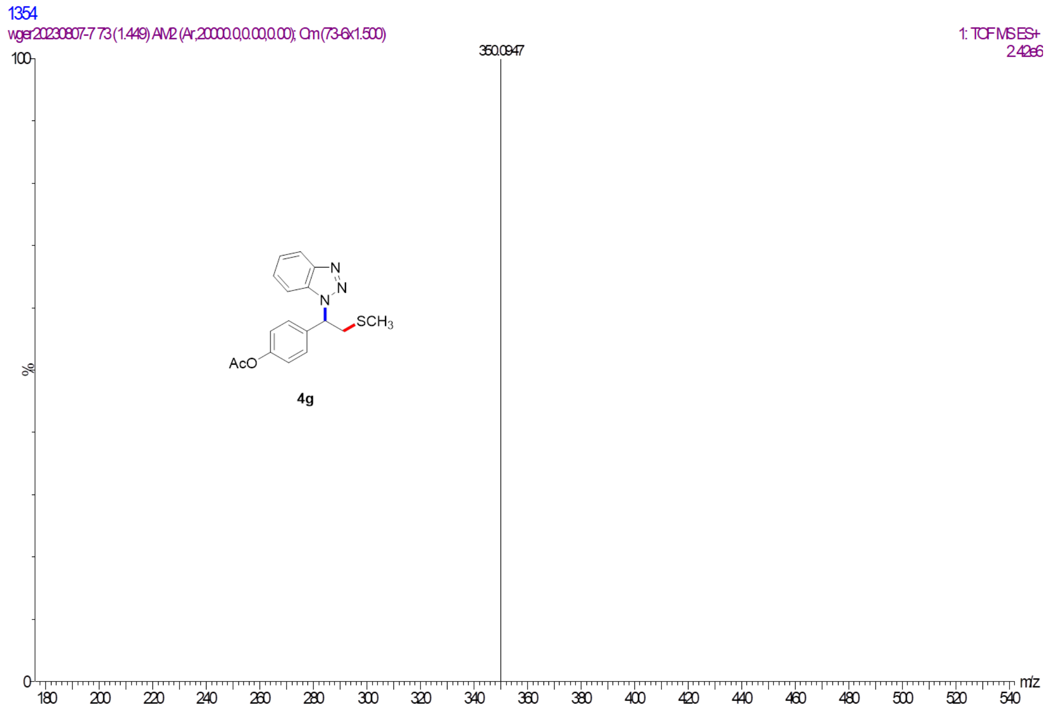
1354
wger20230807-7 73 (1.449) AM2 (Ar,20000.0,0.00,0.00); Cm (73-6x1.500)
1: TOF MS ES+
2.42e6
350.0947
100
%
0
m/z
180 200 220 240 260 280 300 320 340 360 380 400 420 440 460 480 500 520 540
N
N
N
SCH3
AcO
4g

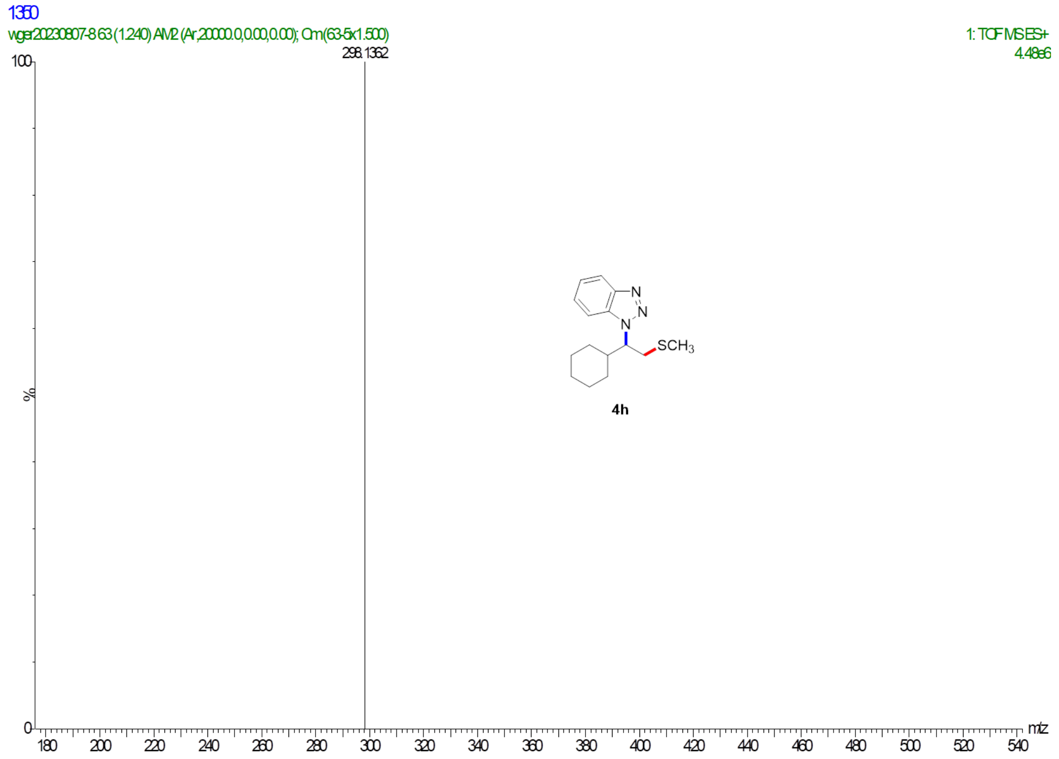
1350
wger20230807-8 63 (1.240) AM2 (Ar,20000.0,0.00,0.00); Cm(63-5x1.500)
1: TOF MS ES+
4.48e6
298.1362
100
%
0
m/z
180 200 220 240 260 280 300 320 340 360 380 400 420 440 460 480 500 520 540
N
N
N
SCH3
4h

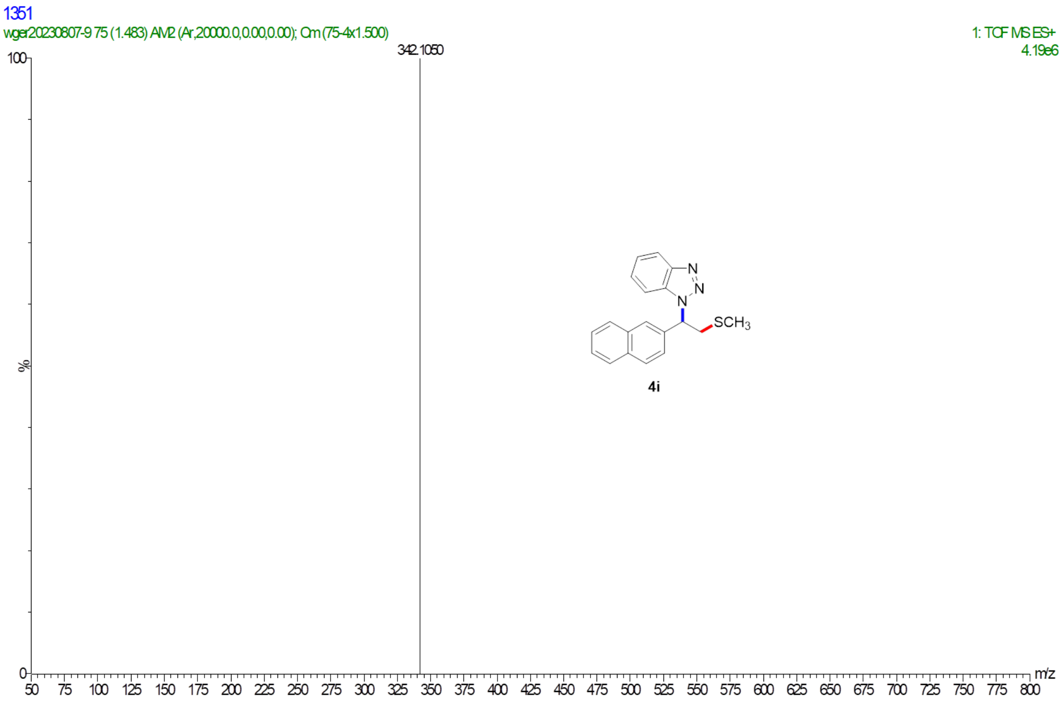
1351
wger20230807-9 75 (1.483) AM2 (Ar,20000.0,0.00,0.00); Cm (75-4x1.500)
1: TOF MS ES+
4.19e6
342.1050
100
%
0
m/z
SCH3
4i

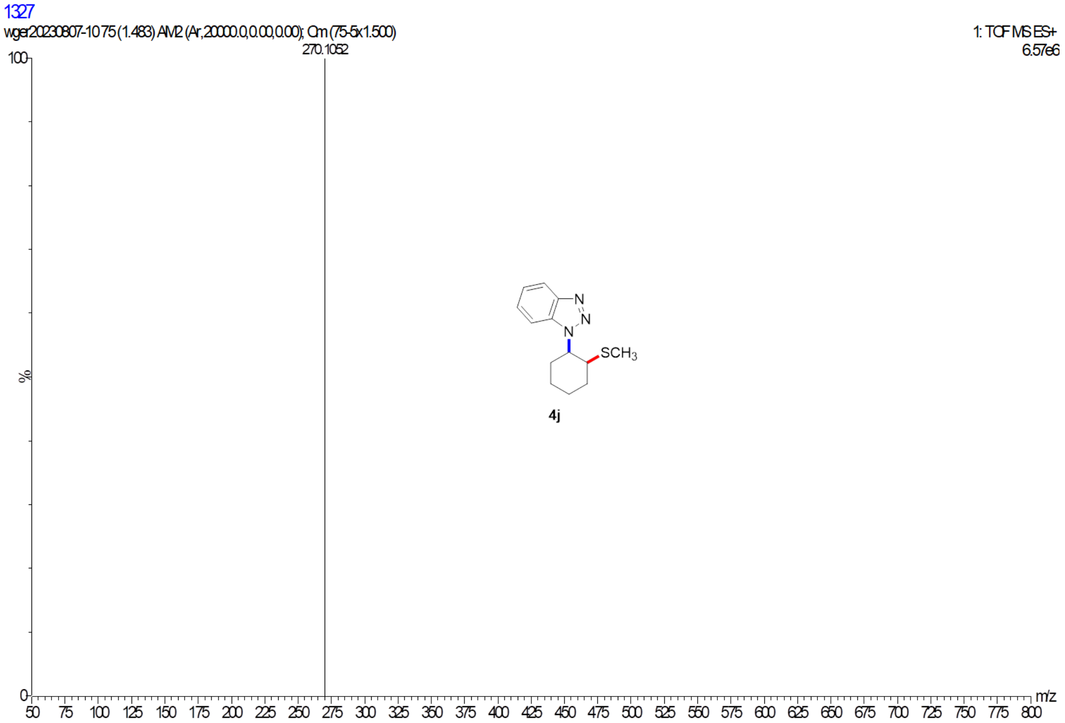
1327
wger20230807-10 75 (1.483) AM2 (Ar,20000.0,0.00,0.00); Cm (75-5x1.500)
1: TOF MS ES+
6.57e6
270.1052
100
%
0
m/z
SCH3
4j

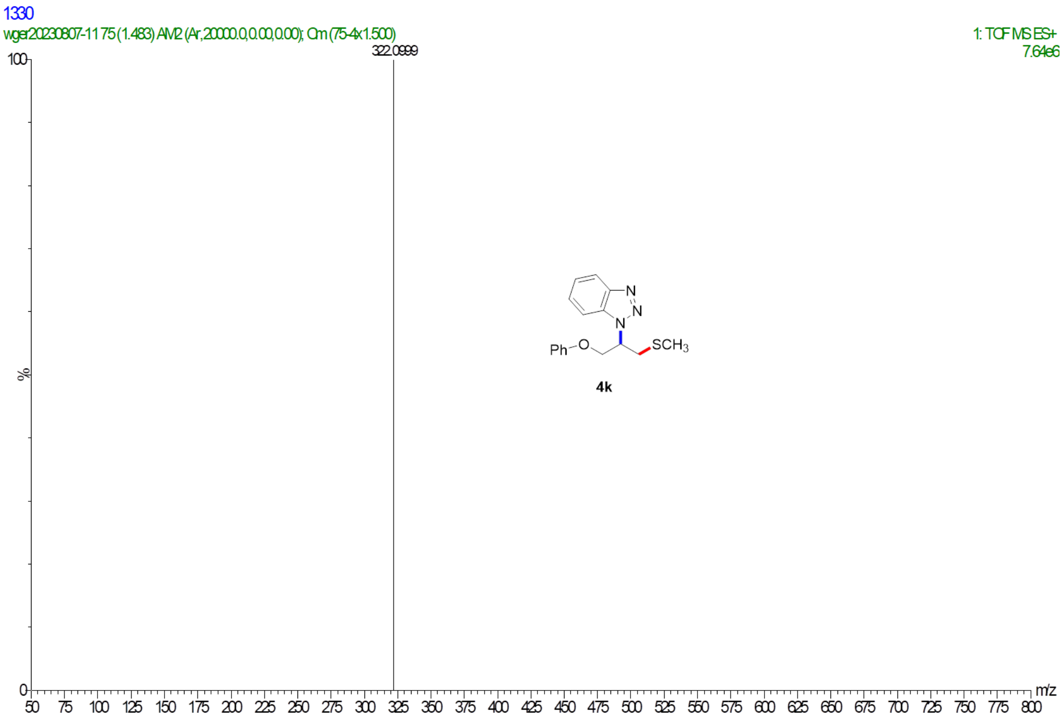
1330
wger20230807-11 75 (1.483) AM2 (Ar,20000.0,0.00,0.00); Cm (75-4x1.500)
1: TOF MS ES+
7.64e6
322.0999
Ph
O
N
N
N
SCH3
4k
%
m/z

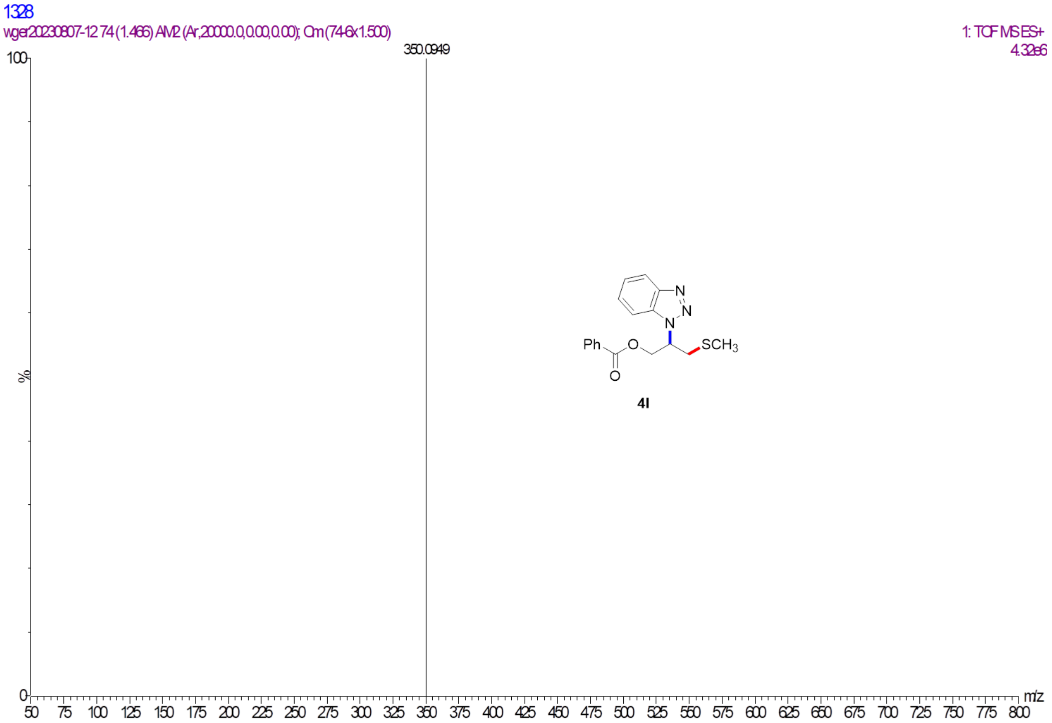
1328
wger20230807-12 74 (1.466) AM2 (Ar,20000.0,0.00,0.00); Cm (74-6x1.500)
1: TOF MS ES+
4.32e6
350.0949
Ph
O
O
N
N
N
SCH3
4l
%
m/z

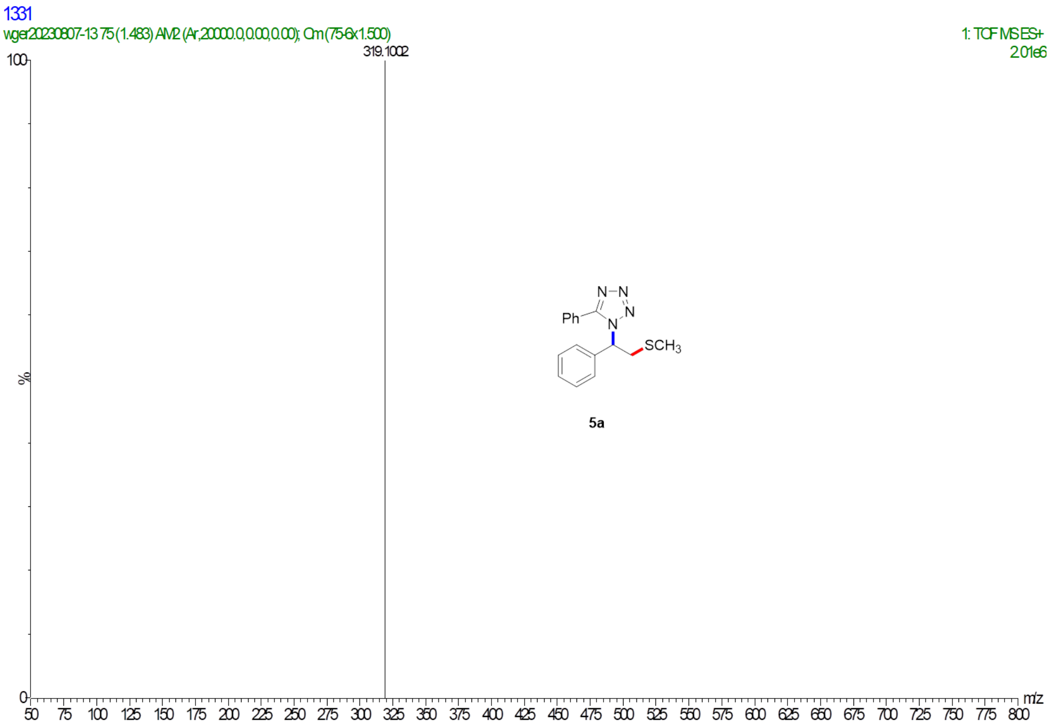
1331
wger20230807-13 75 (1.483) AM2 (Ar,20000.0,0.00,0.00); Cm (75-6x1.500)
1: TOF MS ES+
2.01e6
319.1002
100
%
0
m/z
50 75 100 125 150 175 200 225 250 275 300 325 350 375 400 425 450 475 500 525 550 575 600 625 650 675 700 725 750 775 800
Ph
N
SCH3
5a

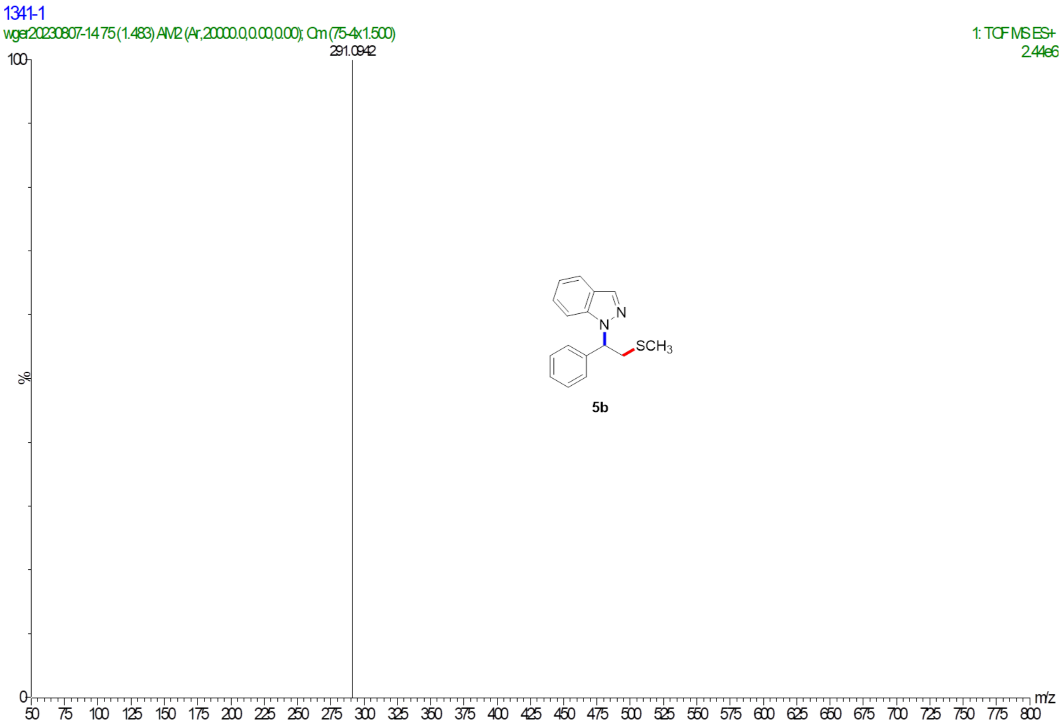
1341-1
wger20230807-14 75 (1.483) AM2 (Ar,20000.0,0.00,0.00); Cm (75-4x1.500)
1: TOF MS ES+
2.44e6
291.0942
100
%
0
m/z
50 75 100 125 150 175 200 225 250 275 300 325 350 375 400 425 450 475 500 525 550 575 600 625 650 675 700 725 750 775 800
N
SCH3
5b

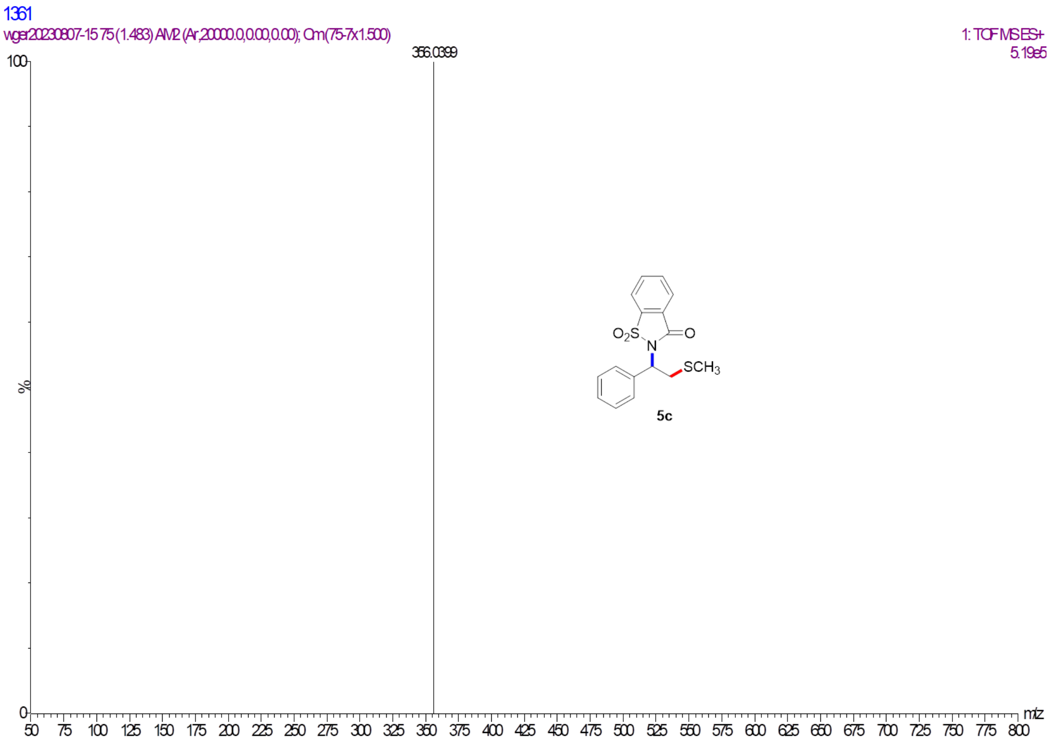
1361
wger20230807-15 75 (1.483) AM2 (Ar,20000.0,0.00,0.00); Cm (75-7x1.500)
1: TOF MS ES+
5.19e5
356.0399
100
%
0
m/z
O2S
N
O
SCH3
5c

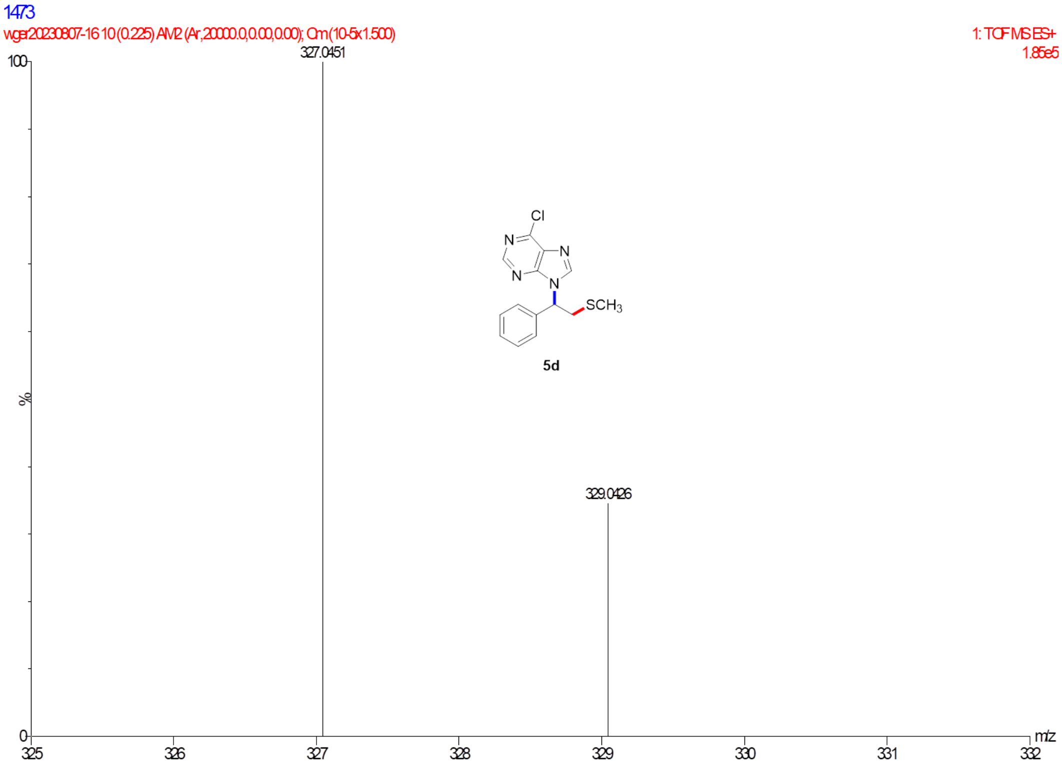
1473
wger20230807-16 10 (0.225) AM2 (Ar,20000.0,0.00,0.00); Cm (10-5x1.500)
1: TOF MS ES+
1.85e5
327.0451
329.0426
100
%
0
m/z
Cl
N
N
N
N
SCH3
5d

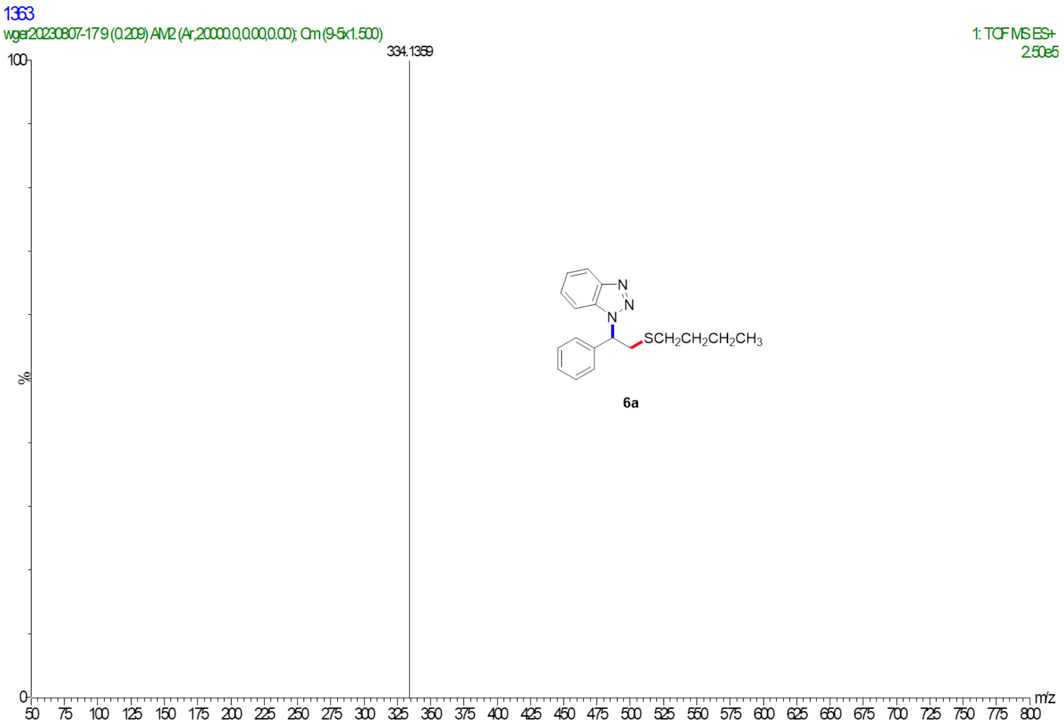
1363
wger20230807-17 9 (0.209) AM2 (Ar,20000.0,0.00,0.00); Cm (9-5x1.500)
1: TOF MS ES+
2.50e5
334.1359
100
%
0
m/z
50 75 100 125 150 175 200 225 250 275 300 325 350 375 400 425 450 475 500 525 550 575 600 625 650 675 700 725 750 775 800
N
N
N
$SCH_2CH_2CH_2CH_3$
6a

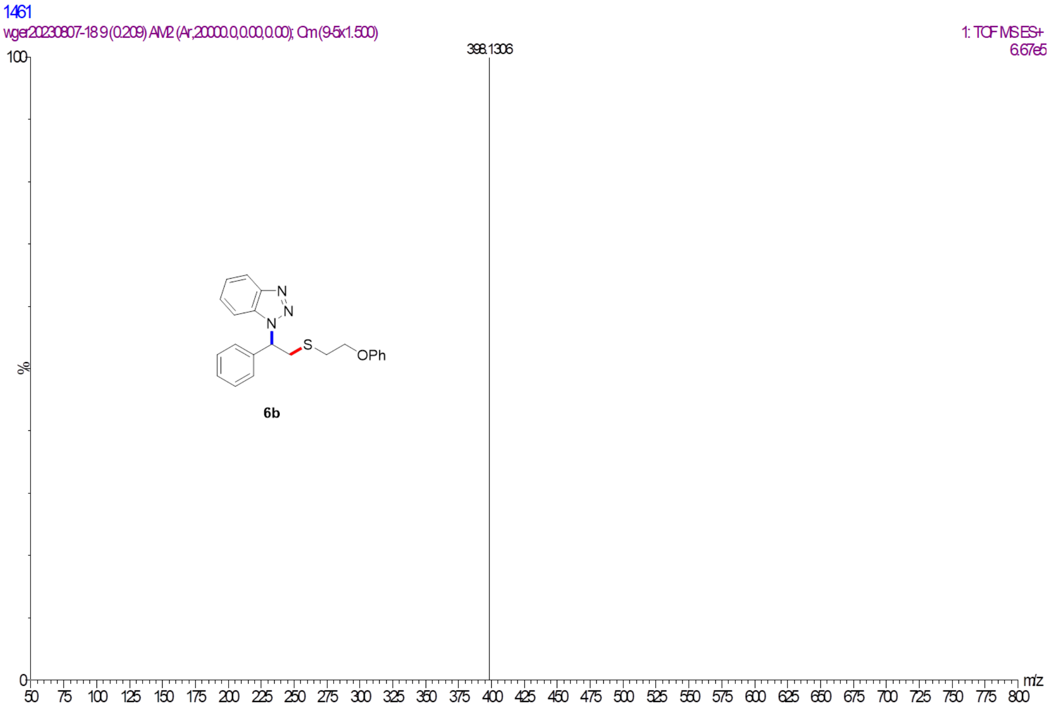
1461
wger20230807-18 9 (0.209) AM2 (Ar,20000.0,0.00,0.00); Cm (9-5x1.500)
1: TOF MS ES+
6.67e5
398.1306
100
%
0
m/z
50 75 100 125 150 175 200 225 250 275 300 325 350 375 400 425 450 475 500 525 550 575 600 625 650 675 700 725 750 775 800
N
N
N
S
OPh
6b

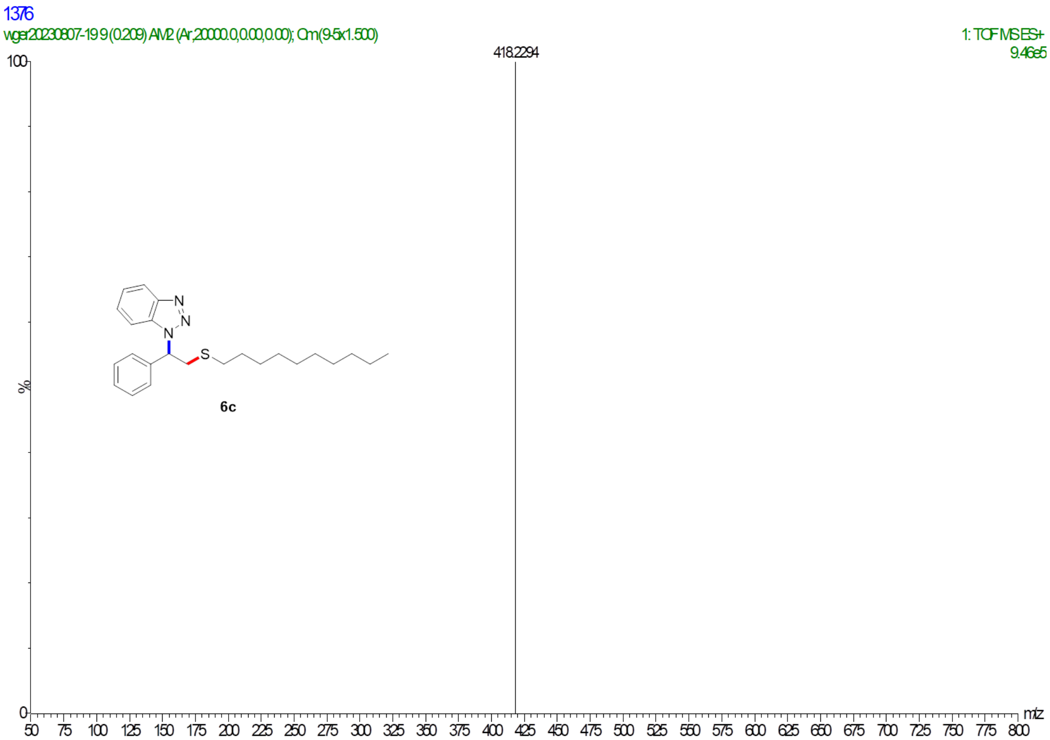
1376
wger20230807-19 9 (0.209) AM2 (Ar,20000.0,0.00,0.00); Cm (9-5x1.500)
1: TOF MS ES+
9.46e5
418.2294
6c
%
m/z

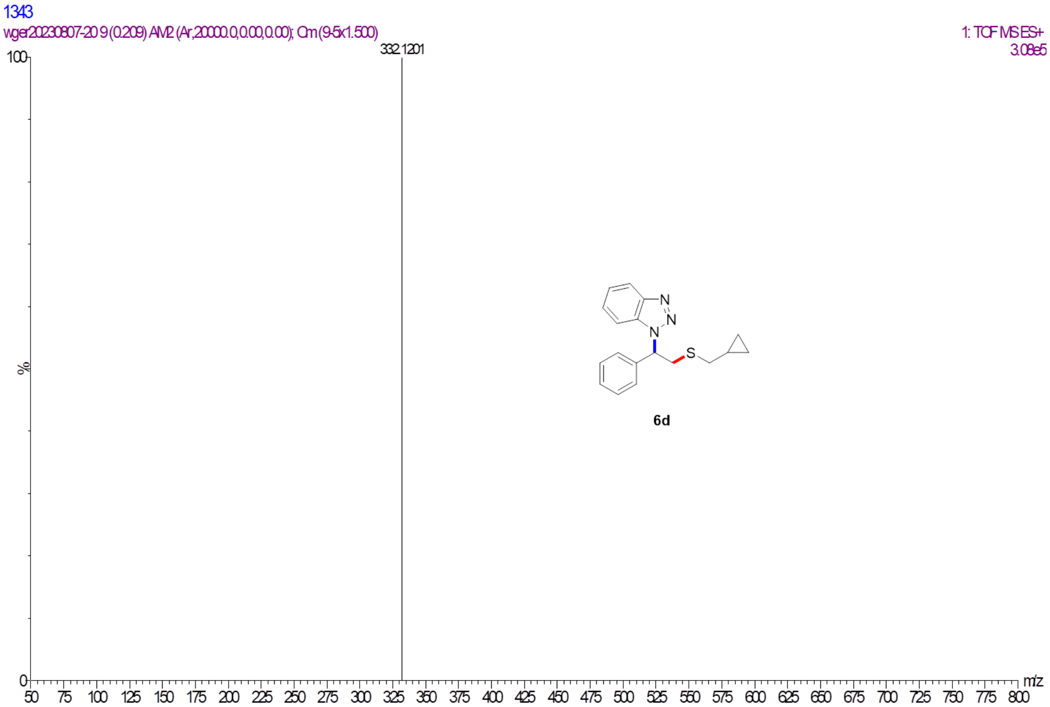
1343
wger20230807-20 9 (0.209) AM2 (Ar,20000.0,0.00,0.00); Cm (9-5x1.500)
1: TOF MS ES+
3.08e5
332.1201
6d
%
m/z

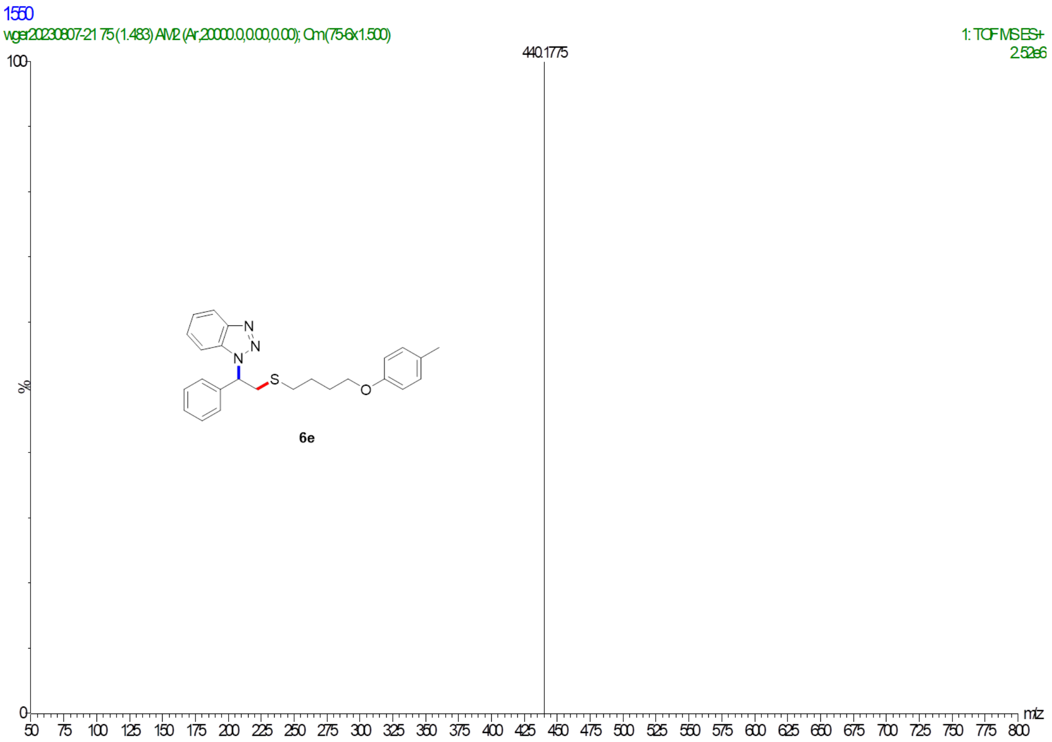
1550
wger20230807-21 75 (1.483) AM2 (Ar,20000.0,0.00,0.00); Cm (75-6x1.500)
1: TOF MS ES+
2.52e6
440.1775
100
%
0
m/z
50 75 100 125 150 175 200 225 250 275 300 325 350 375 400 425 450 475 500 525 550 575 600 625 650 675 700 725 750 775 800
N
N
N
S
O
6e

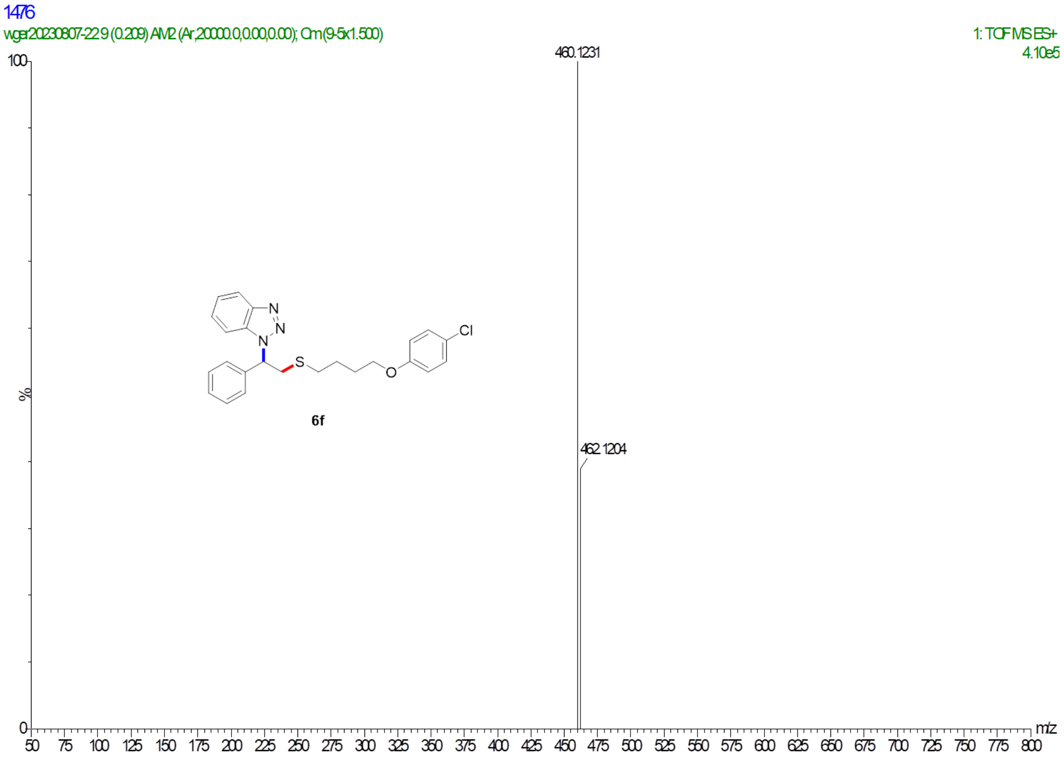
1476
wger20230807-22 9 (0.209) AM2 (Ar,20000.0,0.00,0.00); Cm (9-5x1.500)
1: TOF MS ES+
4.10e5
460.1231
462.1204
100
%
0
m/z
50 75 100 125 150 175 200 225 250 275 300 325 350 375 400 425 450 475 500 525 550 575 600 625 650 675 700 725 750 775 800
N
N
N
S
O
Cl
6f

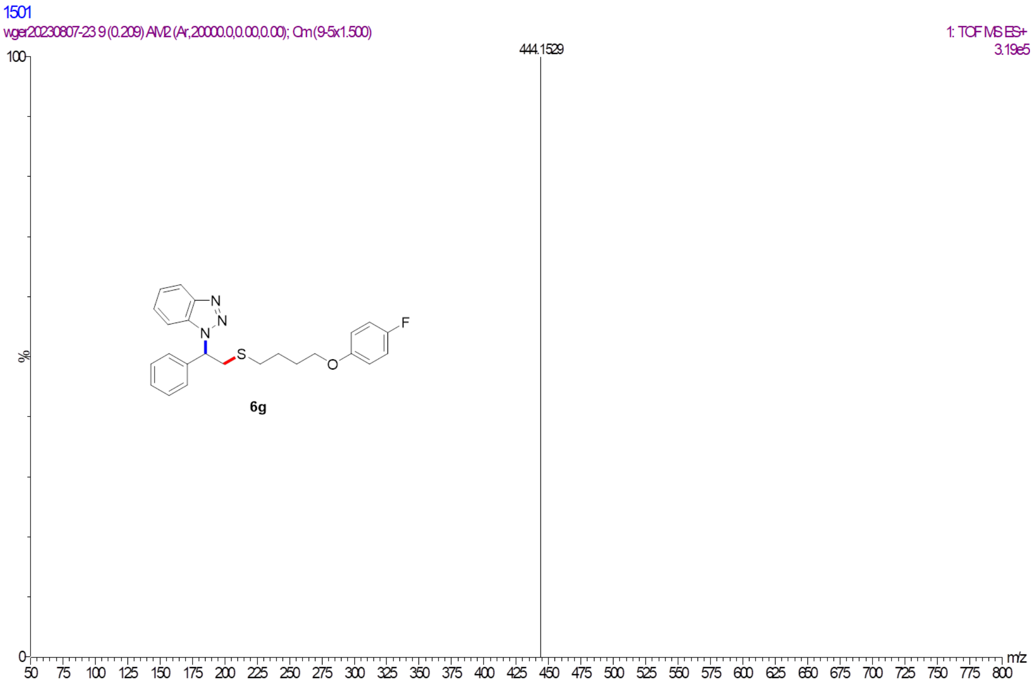
1501
wger20230807-23 9 (0.209) AM2 (Ar,20000.0,0.00,0.00); Cm (9-5x1.500)
1: TOF MS ES+
3.19e5
444.1529
%
m/z
6g

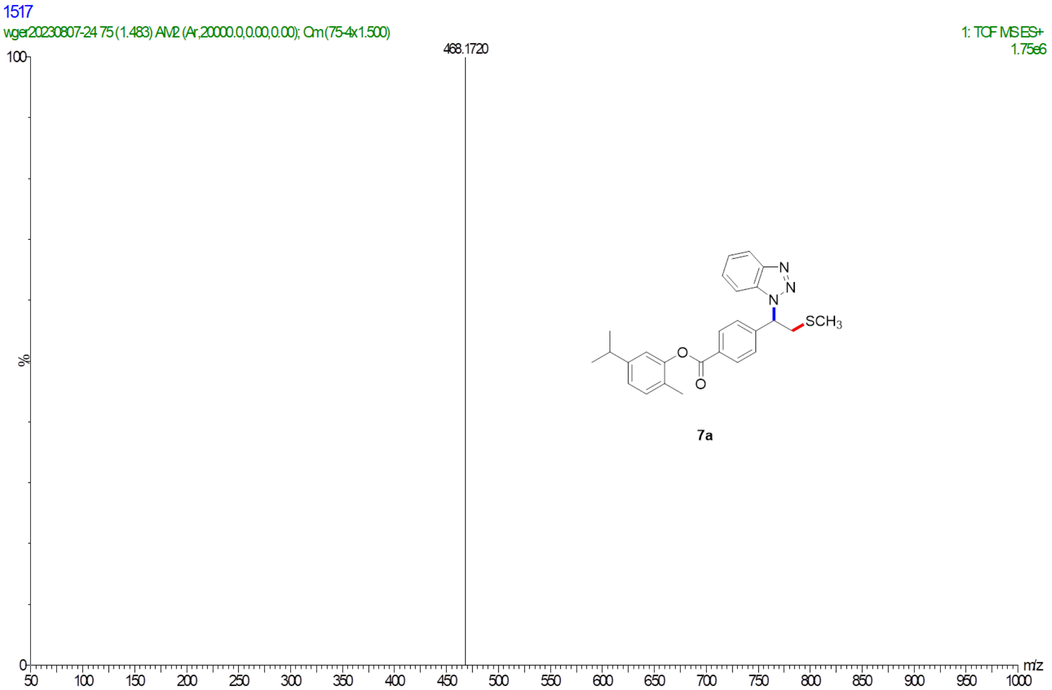
1517
wger20230807-24 75 (1.483) AM2 (Ar,20000.0,0.00,0.00); Cm (75-4x1.500)
1: TOF MS ES+
1.75e6
468.1720
%
m/z
SCH3
7a

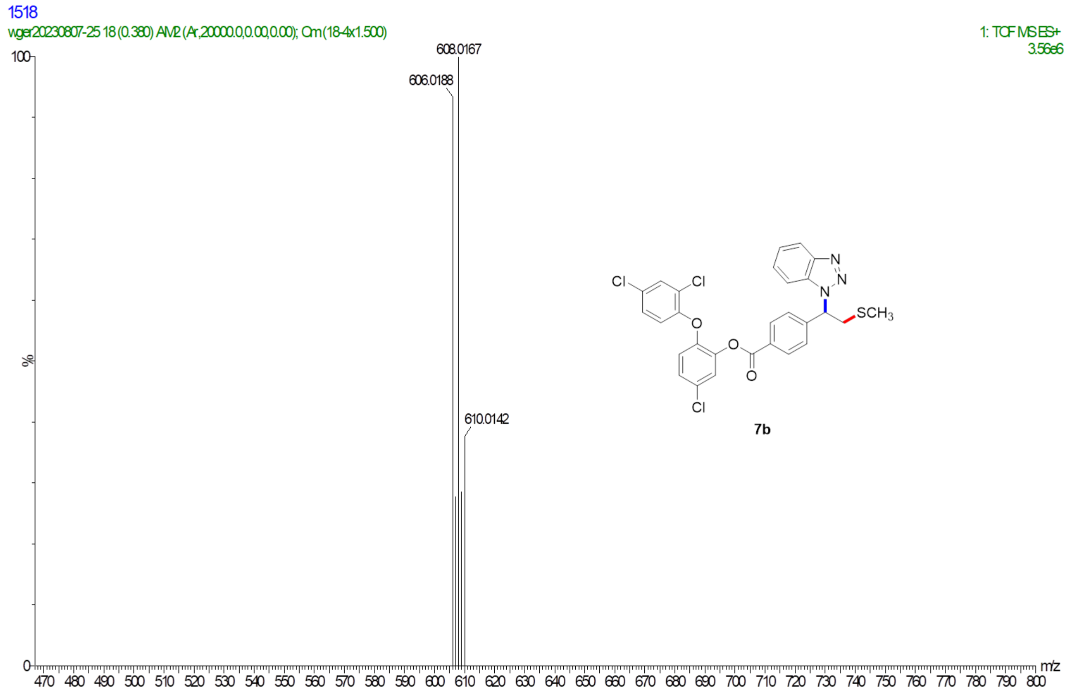
1518
wger20230807-25 18 (0.380) AM2 (Ar,20000.0,0.00,0.00); Cm (18-4x1.500)
1: TOF MS ES+
3.56e6
608.0167
606.0188
610.0142
%
m/z
Cl
Cl
O
O
O
Cl
N
N
N
SCH3
7b
